# Does repeated influenza vaccination attenuate effectiveness? A systematic review and meta-analysis

**DOI:** 10.1016/S2213-2600(22)00266-1

**Published:** 2023-01

**Authors:** Elenor Jones-Gray, Elizabeth J Robinson, Adam J Kucharski, Annette Fox, Sheena G Sullivan

**Affiliations:** a Department of Infectious Diseases, University of Melbourne, Melbourne, VIC, Australia; b WHO Collaborating Centre for Reference and Research on Influenza, Royal Melbourne Hospital, Peter Doherty Institute for Infection and Immunity, Melbourne, VIC, Australia; c Department of Epidemiology, University of California, Los Angeles, CA, USA; d Centre for the Mathematical Modelling of Infectious Diseases (CMMID), London School of Hygiene and Tropical Medicine, London, UK

## Abstract

**Background:**

Influenza vaccines require annual readministration; however, several reports have suggested that repeated vaccination might attenuate the vaccine's effectiveness. We aimed to estimate the reduction in vaccine effectiveness associated with repeated influenza vaccination.

**Methods:**

In this systematic review and meta-analysis, we searched MEDLINE, EMBASE, and CINAHL Complete databases for articles published from Jan 1, 2016, to June 13, 2022, and Web of Science for studies published from database inception to June 13, 2022. For studies published before Jan 1, 2016, we consulted published systematic reviews. Two reviewers (EJ-G and EJR) independently screened, extracted data using a data collection form, assessed studies' risk of bias using the Risk Of Bias In Non-Randomized Studies of Interventions (ROBINS-I) and evaluated the weight of evidence by Grading of Recommendations Assessment, Development, and Evaluation (GRADE). We included observational studies and randomised controlled trials that reported vaccine effectiveness against influenza A(H1N1)pdm09, influenza A(H3N2), or influenza B using four vaccination groups: current season; previous season; current and previous seasons; and neither season (reference). For each study, we calculated the absolute difference in vaccine effectiveness (ΔVE) for current season only and previous season only versus current and previous season vaccination to estimate attenuation associated with repeated vaccination. Pooled vaccine effectiveness and ∆VE were calculated by season, age group, and overall. This study is registered with PROSPERO, CRD42021260242.

**Findings:**

We identified 4979 publications, selected 681 for full review, and included 83 in the systematic review and 41 in meta-analyses. ΔVE for vaccination in both seasons compared with the current season was –9% (95% CI –16 to –1, *I*^2^=0%; low certainty) for influenza A(H1N1)pdm09, –18% (–26 to –11, *I*^2^=7%; low certainty) for influenza A(H3N2), and –7% (–14 to 0, *I*^2^=0%; low certainty) for influenza B, indicating lower protection with consecutive vaccination. However, for all types, A subtypes and B lineages, vaccination in both seasons afforded better protection than not being vaccinated.

**Interpretation:**

Our estimates suggest that, although vaccination in the previous year attenuates vaccine effectiveness, vaccination in two consecutive years provides better protection than does no vaccination. The estimated effects of vaccination in the previous year are concerning and warrant additional investigation, but are not consistent or severe enough to support an alternative vaccination regimen at this time.

**Funding:**

WHO and the US National Institutes of Health.

## Introduction

Influenza vaccines require annual readministration because circulating viruses, especially influenza A(H3N2) viruses,^1^ undergo rapid antigenic drift demanding reconfiguration of the vaccine and because vaccine-induced immunity against homologous strains might wane.^2^, ^3^ Annual seasonal influenza vaccination is currently recommended in some countries.^4^ However, vaccine effectiveness might attenuate with repeated administration.^5^

The first study to report reduced vaccine effectiveness in repeat vaccinees came from a 1970s vaccine trial in an English boarding school, which observed that infection rates were higher for boys vaccinated in the current and previous season than for boys receiving their first vaccination.^5^ A 1999 review of ensuing immunological studies identified that roughly half of published serological studies reported reduced post-vaccination antibody titres against A(H3N2) in people who had received multiple influenza vaccinations compared with those who had received a single influenza vaccination.^6^ Several subsequent studies have shown diminishing post-vaccination antibody responses^7^, ^8^, ^9^, ^10^, ^11^ and diminishing vaccine effectiveness^12^, ^13^, ^14^, ^15^, ^16^, ^17^, ^18^, ^19^ as the number of previous vaccines an individual has been given increases.

These findings indicate that the capacity of vaccination to update immunity against new influenza viruses might be limited by pre-existing immunity.^11^ Hoskins and colleagues^20^ proposed that, by preventing infection-acquired immunity, vaccination increases the risk of infection by an antigenically drifted strain. However, the effects of previous vaccination vary among studies^21^ and seasons,^22^, ^23^ leading to speculation that the effects of pre-existing immunity might depend on the degree of antigenic change between successively encountered strains. The antigenic distance hypothesis is a possible explanation for this phenomenon, including inconsistencies among studies.^24^ The hypothesis posits that, when two vaccine strains are antigenically similar, responses to epitopes in the first vaccine strain dominate, such that repeat vaccination impairs vaccine effectiveness if the circulating strain has changed from the second vaccine strain but enhances vaccine effectiveness if the circulating strain has not changed. In contrast, if the first and second vaccine strains are antigenically distant, repeat vaccination has little effect because responses to the second vaccine strain are not compromised.^16^, ^19^ These effects are not expected each year because of annual differences in the first and second vaccine strains and in the antigenic distance between the second vaccine strain and the circulating strain. However, these effects are seen more often for influenza A(H3N2) viruses,^21^ probably because of higher rates of antigenic drift.^25^

**Research in context**

**Evidence before this study**

We searched MEDLINE, Web of Science, CINAHL Complete, and EMBASE with the terms “influenza”, “vaccines”, “immunization”, “efficacy”, and “effectiveness” without language restriction from database inception to June 13, 2022, and the reference lists of previous systematic reviews on repeat influenza vaccine effectiveness. Studies of any design were included if they investigated the vaccine effectiveness of influenza vaccination in consecutive seasons. Two previous related meta-analyses have summarised influenza A subtypes and influenza B with minimal subgroup analysis and quality appraisal. Our literature search revealed substantially more articles for inclusion since these reviews were published.

**Added value of this study**

We identified 83 observational studies from 30 countries; 41 studies were included in meta-analysis with the earliest season estimates from 2007–08. Our meta-analysis provides up-to-date summary vaccine effectiveness estimates for influenza vaccination in two consecutive seasons for policy planning consideration. We included a critical appraisal of the body of evidence by providing a risk-of-bias assessment for studies included in the meta-analysis. Our protocol is available on Prospero for those wishing to repeat our process. We estimated that vaccine effectiveness against influenza A(H1N1)pdm09 and the influenza B viruses for people vaccinated in both the current and previous seasons were, on average, slightly attenuated compared with effectiveness in people vaccinated in the current season only. Vaccine effectiveness against influenza A(H3N2) was worse overall than for influenza B and displayed a greater loss in effectiveness with repeated vaccination. However, on average, vaccination in both the current and previous seasons afforded better protection than not being vaccinated in either season or vaccination in the previous season only for all types, A subtypes and B lineages examined.

**Implications of all the available evidence**

Annual seasonal influenza vaccination is currently recommended in several countries. Our study contributes to the growing body of evidence on repeated influenza vaccination. We used the Grading of Recommendations Assessment, Development, and Evaluation (GRADE) approach to assess the certainty of the body of evidence by type, A subtype and B lineage, which was low for influenza A(H1N1)pdm09 and influenza B, and very low for influenza A(H3N2). The currently available evidence does not warrant a change to policies that recommend annual vaccination. Our results support current season vaccination regardless of previous season vaccination and suggest that vaccination in any combination of current and previous seasons provided better protection than not being vaccinated.

Concepts regarding the underlying immunological mechanisms have evolved over many years. The initial concept of original antigenic sin suggests that a person's first influenza infection preferentially orients antibodies towards priming epitopes that remain in subsequent strains, often as subdominant epitopes.^26^ Furthermore, the concept of antigenic seniority suggests previous infections have cumulative negative effects on responses to later strains, resulting in higher antibody titres to strains encountered earlier in life.^17^ Immune boosting and interference might account for these concepts, with successive influenza exposures boosting antibody responses to more senior strains that dominate over responses to new epitopes, suggesting that memory responses are invoked.^27^, ^28^ Indeed, studies have shown preferential focusing of antibodies on a conserved epitope among successively encountered strains.^29^, ^30^ Consequently, a future opportunity cost might be created if conserved epitopes are subsequently altered in circulating strains.^31^ Thus, antibody focusing might be linked to antigenic drift and the antigenic distance hypothesis;^24^ for example, a series of similar vaccines containing a shared epitope might promote antibody focusing that would provide little protection if the circulating strain drifts, thereby reducing vaccine effectiveness.

Since many people vaccinated in any one season tend to be vaccinated in every season, any loss in effectiveness presents an important policy consideration since it will affect the majority of those vaccinated. To investigate whether vaccine effectiveness is reduced by repeated vaccination, we did a systematic review and meta-analysis of studies reporting vaccine effectiveness by the previous year's vaccination status.

## Methods

### Search strategy and selection criteria

We followed PRISMA guidelines (appendix pp 2–4) throughout this systematic review and meta-analysis. We searched MEDLINE, EMBASE, and CINAHL Complete databases for articles published from Jan 1, 2016, to June 13, 2022, and Web of Science for studies published in English from database inception to June 13, 2022**.** We also searched reference lists of reviews,^32^, ^33^ and we searched MEDLINE, EMBASE, and CINAHL Complete databases for non-English language studies published up to Dec 31, 2015. Search terms included variations of “influenza”, “vaccines”, “immunization”, “efficacy” and “effectiveness” (appendix p 5). We searched reference lists of eligible studies for additional inclusions and we contacted experts and asked whether they had any unpublished papers.

We included observational studies and randomised controlled trials that reported vaccine effectiveness against laboratory-confirmed influenza for four comparison groups: current season only, previous season only, current and previous seasons, and neither season (reference group). For studies not in English, we required an English language abstract.

Two reviewers (EJ-G and EJR) independently conducted screening, data extraction, and risk of bias analyses. Titles and abstracts were initially screened, followed by full-text screening according to predefined exclusion and inclusion criteria using Covidence. Conflicts between reviewers were resolved via consensus or consultation with a third reviewer (SGS). Non-English language studies were evaluated by individuals fluent in the relevant language.

### Data analysis

We extracted data using a standardised form after the removal of duplicates (appendix pp 6–7). Extracted data included study and patient characteristics, as well as vaccine effectiveness or odds ratio (OR; 95% CI) estimates for individual seasons by age group and influenza virus type. We contacted authors of publications that did not provide the numerical values required for meta-analysis. We preferentially recorded adjusted, rather than crude, estimates. If the publication reanalysed already published data, we extracted the most recent published estimate.

We assessed study quality using Risk of Bias in Non-Randomized Studies of Interventions (ROBINS-I).^34^ We graded the certainty of evidence presented in the meta-analysis using the Grading of Recommendations Assessment, Development, and Evaluation (GRADE) approach.^35^

The absolute difference in vaccine effectiveness (ΔVE) for people who were vaccinated in the current and previous (VE_CP_) seasons and those vaccinated in the current season only (VE_C_) was calculated as: ΔVE = VE_CP_– VE_C_. ΔVE>0 implies higher vaccine effectiveness if vaccinated in the current and previous seasons than in the current season alone. We calculated CIs for ΔVE by bootstrapping 1000 samples for VE_C_ and VE_CP_ (appendix pp 8–9).^36^ The absolute difference in vaccine effectiveness for people vaccinated in the current and previous season against the previous season only ΔVE_P_ was also calculated. This relationship might be less biased by confounding^37^ and might be more relevant to frequent vaccinees^32^ because it considers potential residual effects of vaccination in the preceding season.^38^

For the meta-analysis, we estimated pooled vaccine effectiveness with 95% CIs for influenza A(H1N1)pdm09, influenza A(H3N2), and influenza B by season and age group for each vaccination group (ie, current season only, previous season only, current and previous seasons). If studies did not report estimates for all four comparison groups, we used all available estimates. We did not incorporate studies with estimates or 95% CI values equal to 100% vaccine effectiveness**.**

The primary analysis for each type, A subtype or B lineage used vaccine effectiveness estimates for the broadest age group (eg, all ages or ≥9 years). Estimates from studies that only reported vaccine effectiveness for a particular age group (eg, children) were only included in age group estimates. Southern hemisphere estimates were grouped with northern hemisphere estimates with the same vaccine formulation. Inpatient and outpatient studies were pooled, as these have been found to be broadly consistent.^36^ We did age subgroup analyses considering three age groups: children, adults, and older adults (as defined in each study). For influenza B, we calculated separate pooled estimates for the infecting lineage and also calculated separate pooled estimates for the lineage included in trivalent influenza vaccines. We evaluated statistical heterogeneity using Cochran's Q and the *I*^2^ statistic. In addition, we calculated both random-effects and fixed-effect models as discrepancies could indicate instability in the pooled estimates.^39^ Publication bias was investigated by funnel plots and formally tested using Egger's test, in which at least ten estimates were available.^40^, ^41^

We did several sensitivity analyses to assess the robustness of pooled estimates to study design: inclusion of studies using non-PCR diagnostics tests; restriction of data to the northern hemisphere; restriction of data to outpatient studies; restriction of data to test-negative studies; and exclusion of studies with serious, critical, or no information risk of bias assessed by ROBINS-I.

All analyses were done in R (version 3.6.1) using the package metafor for meta-analyses and the robvis package for risk of bias visualisations.

This systematic review and meta-analysis was preregistered on PROSPERO (CRD42021260242).

### Role of the funding source

The WHO SAGE Working Group on Influenza defined the scope of the review and provided feedback on the final report. The US National Institutes of Health had no role in study design, data collection, analysis, or interpretation.

## Results

**Figure 1:**
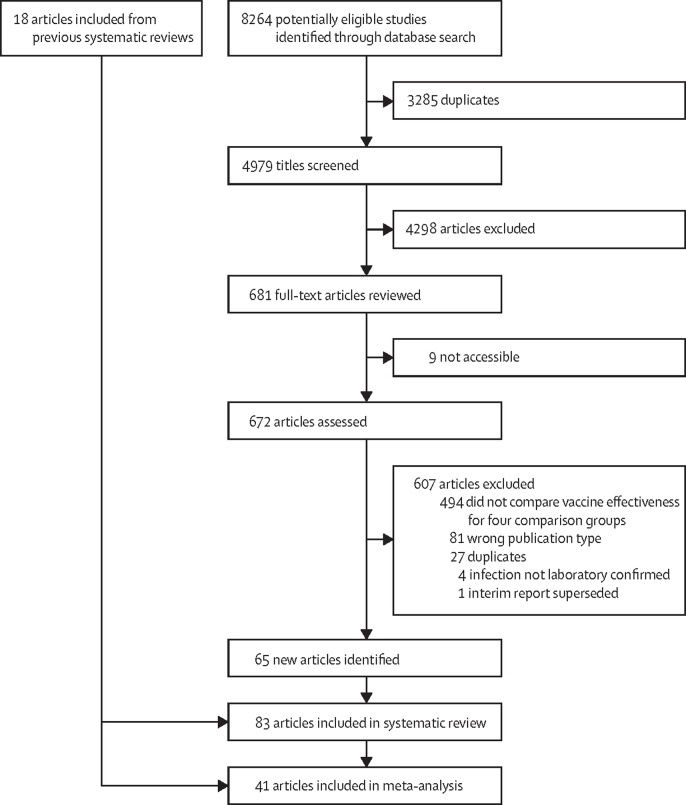
Study selection

We identified 4979 unduplicated publications and selected 681 for full-text review. 83 publications met the eligibility criteria (appendix pp 10–14; figure 1). The characteristics of the 83 studies are presented in table 1 and the appendix (pp 15–39). All included studies were observational, including five cohort studies, six case-control studies, and 72 test-negative studies. Most were done in Europe and North America. The earliest eligible study was published Feb 17, 2011, and the earliest season was 2007–08.

**Table 1:** Study characteristics of 83 articles that met eligibility criteria for assessment of current and previous season vaccine effectiveness

	**Country**	**Design**	**Setting**	**Age range**	**Influenza virus type**	**Current season studied**	**Number of years previous vaccination**
Boddington et al (2019)^42^	England	Test negative	Inpatient	2–16 years	Any	2015–16	1
Buchan et al (2017)^43^	Canada	Test negative	Inpatient	6–59 months	A(H1N1)pdm09, A(H3N2), and B	2010–11 to 2013–14	1
Buchan et al (2018)^44^	Canada	Test negative	Inpatient and outpatient	2–17 years	A(H1N1)pdm09, A(H3N2), and B	2012–13, 2013–14, 2014–15, and 2015–16	1
Casado et al (2016)^45^	Spain	Test negative	Inpatient	≥65 years	Any	2013–2014	1
Casado et al (2018)^46^	Spain	Case control	Inpatient	≥65 years	Any	2013–2014 and 2014–15	3
Castilla et al (2011)^47^	Spain	Nested case control	Inpatient and outpatient	All ages	Any	2010–11	1
Castilla et al (2016)^48^	Spain	Test negative	Inpatient and outpatient	≥6 months	A(H3N2) and B	2014–15	2
Castilla et al (2017)^49^	Spain	Test negative	Inpatient and outpatient	≥9 years	A(H3N2)	2016–17	4
Castilla et al (2018)^50^	Spain	Test negative	Inpatient and outpatient	≥9 years	A(H3N2) and B	2017–18	5
Castilla et al (2018)^50^	Spain	Test negative	Inpatient and outpatient	≥9 years	Any	2017–18	5
Castilla et al (2020)^51^	Spain	Test negative	Inpatient and outpatient	≥9 years	Any	2018–19	3
Cheng et al (2017)^52^	Australia	Test negative	Inpatient	>9 years	A(H1N1)pdm09, A(H3N2), and B	2011–15	1
Dominguez et al (2017)^53^	Spain	Case control	Inpatient	≥65 years	Any	2014–15	1
El Omeiri et al* (2018)^54^	Latin America	Test negative	Inpatient	≥60 years	A(H1N1)pdm09 and any	2013	1
Ferdinands et al (2019)^55^	USA	Test negative	Inpatient	≥18 years	Any	2015–16	1
Flannery et al (2019)^56^	USA	Test negative	Outpatient	≥9 years	A(H3N2) and B	2016–17	1
Fu et al† (2015)^57^	China	Case control	Outpatient	3–6 years	A(H1N1)pdm09	2012–13	1
Gaglani et al* (2016)^58^	USA	Test negative	Outpatient	≥50 years, 18–49 years, and 9–17 years	A(H1N1)pdm09	2013–14	1
Gaglani et al* (2016)^58^	USA	Test negative	Outpatient	≥9 years	A(H1N1)pdm09	2013–14	1 and 4
Gherasim et al† (2017)^59^	Spain	Test negative	Outpatient	≥9 years	A(H1N1)pdm09	2010–11, 2013–14, and 2015–16	1
Gherasim et al† (2017)^59^	Spain	Test negative	Outpatient	≥9 years	A(H3N2)	2011–12, 2013–14, and 2014–15	1
Gherasim et al† (2017)^59^	Spain	Test negative	Outpatient	≥9 years	B	2010–11, 2012–13, 2014–15, and 2015–16	1
Grijalva et al (2021)^60^	USA	Test negative	Inpatient	≥18 years	Any	2019–20	1
Jackson et al* (2017)^61^	USA	Test negative	Outpatient	≥9 years	A(H1N1)pdm09, and B/Victoria, and B/Yamagata	2015–16	1
Jiménez-Jorge et al (2012)^62^	Spain	Test negative	Outpatient	0–95 years	A(H1N1)pdm09	2010–11	1
Kim et al* (2021)^38^	USA	Test negative	Outpatient	≥9 years	A(H1N1)pdm09	2013–14 and 2015–16	1
Kim et al* (2021)^38^	USA	Test negative	Outpatient	≥9 years	A(H3N2)	2012–13, 2014–15, 2016–17, and 2017–18	1
Kim et al* (2021)^38^	USA	Test negative	Outpatient	≥9 years	B	2012–13, 2014–15, 2015–16, 2016–17, and 2017–18	1
Kissling et al* (2018)^63^	Europe	Test negative	Outpatient	≥9 years	A(H1N1)pdm09	2015–16	1
Kissling et al* (2018)^63^	Europe	Test negative	Outpatient	15–64 years	B	2015–16	1
Kissling et al* (2019)^64^	Europe	Test negative	Outpatient	≥9 years	A(H3N2)	2018–19	1
Kissling et al* (2019)^65^	Europe	Test negative	Outpatient	≥9 years	A(H1N1)pdm09	2017–18	1
Kissling et al* (2019)^65^	Europe	Test negative	Outpatient	≥9 years	A(H3N2)	2016–17 and 2017–18	1
Kwong et al (2020)^66^	Canada	Test negative	Inpatient and outpatient	>65 years	Any	2010–11 to 2015–16	1
Kwong et al (2020)^66^	Canada	Test negative	Inpatient and outpatient	≥70 years	Any	2010–11 to 2015–16	5
Kwong et al (2020)^66^	Canada	Test negative	Inpatient and outpatient	≥75 years	Any	2010–11 to 2015–16	10
Ma et al (2017)^67^	China	Test negative	Outpatient	≥6 months	Any	2014–15	1
Martinez-Baz et al (2013)^68^	Spain	Test negative	Inpatient and outpatient	≥6 months	A(H1N1)pdm09	2010–11	1
Martinez-Baz et al (2017)^69^	Spain	Test negative	Inpatient and outpatient	≥6 months	A(H1N1)pdm09	2010–11, 2012–13, 2013–14, and 2015–16	1
Martinez-Baz et al (2017)^69^	Spain	Test negative	Inpatient and outpatient	≥9 months	A(H1N1)pdm09	2010–11, 2012–13, 2013–14, and 2015–16	1–6
Martinez-Baz et al (2021)^70^	Spain	Test negative	Inpatient, and outpatient	≥9 years	A(H1N1)pdm09	2012–13, 2013–14, 2015–16, 2017–18, and 2018–19	1, 3, and 5
Martinez-Baz et al (2021)^70^	Spain	Test negative	Inpatient	≥9 years	A(H3N2)	2011–12, 2013–14, 2014–15, 2016–17, 2017–18, and 2018–19	1, 3, and 5
Martinez-Baz et al (2021)^70^	Spain	Test negative	Outpatient	≥9 years	A(H3N2)	2011–12, 2013–14, 2014–15, 2015–16, 2016–17, 2017–18, and 2018–19	1, 3, and 5
Martinez-Baz et al (2021)^70^	Spain	Test negative	Inpatient and outpatient	≥9 years	B	2011–12, 2012–13, 2014–15, 2015–16, and 2017–18	1, 3, and 5
Martinez-Baz et al (2021)^71^	Spain	Test negative	Inpatient	9–64 years	Any	2013–14 to 2018–19	1 and 5
Martinez-Baz et al (2021)^71^	Spain	Test negative	Inpatient	≥64 years	Any	2013–14 to 2018–19	1 and 5
McLean et al* (2014)^22^	USA	Test negative	Outpatient	≥9 years	A(H3N2)	2004–05 to 2007–08 and 2010–11 to 2012–13	5
McLean et al* (2014)^22^	USA	Test negative	Outpatient	≥9 years	A(H3N2)	2004–05 to 2007–08, 2010–11 to 2012–13, 2007–08, and 2012–13	1
McLean et al* (2014)^22^	USA	Test negative	Outpatient	≥9 years	B	2004–05 to 2008–09 and 2010–11 to 2012–13	1 and 5
McLean et al* (2015)^72^	USA	Test negative	Outpatient	≥9 years	A(H3N2), B/Yamagata	2012–13	1
McLean et al* (2017)^73^	USA	Test negative	Outpatient	2–17 years	A(H3N2)	2014–15	1
McLean et al† (2018)^74^	USA	Test negative	Outpatient	2–17 years	A(H1N1)pdm09	2013–14 and 2015–16	1, 2, and 3
McLean et al† (2018)^74^	USA	Test negative	Outpatient	2–17 years	A(H3N2)	2014–15	1, 2, and 3
McLean et al† (2018)^74^	USA	Test negative	Outpatient	2–17 years	B/Victoria	2014–15 and 2015–16	1
McLean et al† (2018)^74^	USA	Test negative	Outpatient	2–17 years	B/Yamagata	2013–14, 2014–15, and 2015–16	1
McLean et al† (2018)^74^	USA	Test negative	Outpatient	2–17 years	B	2013–14, 2014–15, and 2015–16	1, 2, and 3
Mira-Iglesias et al (2018)^75^	Spain	Test negative	Inpatient	≥60 years	Any	2016–17	2
Mira-Iglesias et al (2019)^76^	Spain	Test negative	Inpatient	≥60 years	A(H1N1)pdm09, A(H3N2), and B/Yamagata	2017–18	2
Nichols et al† (2019)^77^	Canada	Test negative	Inpatient	≥16 years	A(H1N1)pdm09	2011–2012, 2012–13, and 2013–14	1
Nichols et al† (2019)^77^	Canada	Test negative	Inpatient	≥16 years	A(H3N2) and B	2011–12, 2012–13, 2013–14, and 2014–15	1
Ohmit et al* (2014)^13^	USA	Test negative	Outpatient	≥9 years	A(H3N2)	2011–12	1
Ohmit et al (2015)^78^	USA	Prospective cohort	Community	≥9 years	Any	2012–13	1
Ohmit et al† (2016)^79^	USA	Prospective cohort	Community	<9 years and ≥9 years	A(H1N1)pdm09	2013–14	1
Ortqvist et al (2018)^80^	Sweden	Retrospective cohort	Inpatient and outpatient	≥66 years	Any	2015–16 and 2016–17	1
Ortqvist et al (2018)^80^	Sweden	Retrospective cohort	Inpatient and outpatient	≥70 years	Any	2015–16 and 2016–17	4 and 5
Pebody et al* (2013)^81^	UK	Test negative	Outpatient	All	A(H1N1)pdm09 and B	2010–11	1
Pebody et al† (2017)^82^	UK	Test negative	Outpatient	≥18 years and 2–17 years	A(H3N2)	2016–17	1
Pebody et al* (2019)^83^	UK	Test negative	Outpatient	≥18 years and 2–17 years	A(H3N2), B	2017–18	1
Pebody et al* (2020)^84^	England	Test negative	Inpatient	2–17 years	A(H1N1)pdm09 and A(H1N1)	2018–19	1
Pebody et al* (2020)^85^	England	Test negative	Inpatient	≥65 years	A(H1N1)pdm09 and A(H1N1)	2018–19	1
Pebody et al (2020)^86^	UK	Test negative	Outpatient	All ages	Any	2018–19	1
Petrie et al* (2016)^87^	USA	Test negative	Inpatient	≥18 years	A(H3N2)	2014–15	1
Petrie et al* (2017)^88^	USA	Prospective cohort	Community	≥9 years and 3–8 years	A(H3N2) and B/Yamagata	2014–15	1 and 2
Powell et al (2020)^89^	USA	Test negative	Inpatient and outpatient	6 months to 18 years	Any	2017–18	1
Rao et al (2021)^90^	USA	Test negative	Outpatient	6 months to 8 years	Any	2016–17 and 2017–18	1
Rondy et al* (2015)^91^	Europe	Test negative	Inpatient	≥18 years	A(H1N1)pdm09, A(H3N2), and B	2012–13	1
Rondy et al* (2017)^92^	Europe	Test negative	Inpatient	≥65 years	A(H3N2)	2016–17	1
Rondy et al (2017)^93^	Europe	Test negative	Inpatient	≥65 years	A(H1N1)pdm09	2012–13, 2013–14, and 2015–16	2
Rondy et al (2017)^93^	Europe	Test negative	Inpatient	≥65 years	A(H3N2)	2011–12 and 2013–14	2
Rondy et al (2017)^93^	Europe	Test negative	Inpatient	≥65 years	B	2012–13 and 2015–16	2
Rose et al (2020)^94^	Europe	Test negative	Inpatient	≥65 years	A(H3N2) and B	2017–18	2
Saito et al (2017)^95^	Japan	Test negative	Outpatient	≥2 years	A	2009–10, 2010–11, and 2011–12	1
Saito et al (2018)^96^	Japan	Test negative	Outpatient	9–18 years	A and B	2011–12, 2012–13, and 2013–14	1 and 3
Shinjoh et al (2018)^97^	Japan	Test negative	Outpatient	2–15 years	Any and B	2016–17	1
Simpson et al (2015)^98^	Scotland	Test negative	Outpatient	All ages	Any	2000–01 to 2008–09	1
Skowronski et al* (2012)^99^	Canada	Test negative	Outpatient	≥2 years	A(H1N1)pdm09	2010–11	1
Skowronski et al* (2014)^100^	Canada	Test negative	Outpatient	≥2 years	A(H3N2), B, B/Victoria, and B/Yamagata	2012–13	1
Skowronski et al* (2014)^101^	Canada	Test negative	Outpatient	≥2 years	A(H1N1)pdm09, A(H3N2), B, B/Victoria, and B/Yamagata	2011–12	1
Skowronski et al* (2015)^14^	Canada	Test negative	Outpatient	≥2 years	A(H1N1)pdm09, B/Yamagata, and B	2013–14	1
Skowronski et al* (2016)^18^	Canada	Test negative	Outpatient	≥2 years and 20–64 years	A(H3N2), B, and B/Yamagata	2014–15	1
Skowronski et al* (2016)^18^	Canada	Test negative	Outpatient	≥3 years	A(H3N2), B, and B/Yamagata	2014–15	2
Skowronski et al† (2017)^16^	Canada	Test negative	Outpatient	≥9 years	A(H3N2)	2010–11, 2012–13, and 2014–15	1 and 2
Skowronski et al* (2017)^17^	Canada	Test negative	Outpatient	≥9 years	A(H1N1)pdm09, B, and B/Victoria	2015–16	1 and 2
Skowronski et al† (2019)^102^	Canada	Test negative	Outpatient	≥9 years	B/Yamagata	2011–12, 2014–15, and 2017–18	1
Skowronski et al* (2019)^103^	Canada	Test negative	Outpatient	≥9 years	A(H3N2)	2018–19	1
Skowronski et al* (2020)^104^	Canada	Test negative	Outpatient	≥9 years	A(H3N2)	2016–17 and 2017–18	1
Smithgall et al (2016)^105^	USA	Test negative	Community	>6 months	Any	2013–14	1
Song et al* (2020)^106^	China	Test negative	Inpatient and outpatient	≥65 years	B	2012–13, 2013–14, and 2014–15	1
Sullivan et al (2013)^15^	Australia	Test negative	Outpatient	≥9 years	Any	2011, 2012	1
Sullivan et al† (2017)^19^	Australia	Test negative	Outpatient	All ages	A(H3N2) and B	2017	1
Syrjänen et al (2014)^107^	Finland	Prospective cohort	Community	18–75 years	A(H1N1)pdm09	2010–11	1
Thompson et al (2014)^108^	USA	Test negative	Community	Not specified	A(H1N1)pdm09, A(H3N2), and B	2010–11 and 2011–12	1
Thompson et al* (2016)^109^	USA	Test negative	Outpatient	2–8 years	A(H3N2)	2011–12 and 2012–13	1
Thompson et al* (2016)^109^	USA	Test negative	Outpatient	2–8 years	B	2012–13	1
Valenciano et al (2016)^110^	Europe	Test negative	Outpatient	≥18 years	A(H1N1)pdm09, A(H3N2), and B	2014–15	1
Valenciano et al* (2018)^23^	Europe	Test negative	Outpatient	≥9 years	A(H1N1)pdm09	2012–13, 2013–14, 2014–15, and 2015–16	1
Valenciano et al* (2018)^23^	Europe	Test negative	Outpatient	≥9 years	A(H3N2)	2011–12, 2013–14, 2014–15, and 2016–17	1
Valenciano et al* (2018)^23^	Europe	Test negative	Outpatient	≥9 years	B	2012–13, 2014–15, and 2015–16	1
Zhang et al (2017)^111^	China	Case control	Community	6–18 years	A	2014–15	1
Zhang et al* (2018)^112^	China	Test negative	Outpatient	≥2 years	A(H1N1)pdm09 and A(H3N2)	2015–16	1
Zhang et al* (2020)^113^	China	Case control	Community	6–19 years	A(H1N1)pdm09 and A(H3N2)	2016–17	1
Zimmerman et al* (2016)^114^	USA	Test negative	Outpatient	≥9 years	A(H3N2) and B/Yamagata	2014–15	1

ROBINS-I=Risk of Bias in Non-Randomized Studies of Interventions.

* Study was included in meta-analysis and was assessed as being at moderate risk of bias by the ROBINS-I.

† Study was included in meta-analysis and was assessed as being at serious risk of bias by the ROBINS-I.

Influenza infection was identified by RT-PCR in 79 (95%) studies and by a rapid diagnostic test in four (5%) studies.^89^, ^95^, ^96^, ^97^ 43 (51%) of 83 studies were done in outpatient settings, 19 (23%) in inpatient settings,^42^, ^43^, ^45^, ^46^, ^52^, ^53^, ^54^, ^55^, ^60^, ^71^, ^76^, ^77^, ^84^, ^85^, ^87^, ^91^, ^92^, ^93^, ^94^ 13 (16%) in outpatient and inpatient settings,^44^, ^47^, ^48^, ^49^, ^50^, ^51^, ^66^, ^68^, ^69^, ^70^, ^80^, ^89^, ^106^ and eight (10%) in community settings.^8^, ^79^, ^88^, ^105^, ^107^, ^108^, ^111^, ^113^

Influenza vaccination was confirmed by medical record or registry in 41 (49%) studies,^13^, ^22^, ^42^, ^43^, ^44^, ^45^, ^46^, ^47^, ^48^, ^49^, ^50^, ^51^, ^54^, ^57^, ^58^, ^60^, ^66^, ^67^, ^68^, ^69^, ^70^, ^71^, ^72^, ^73^, ^74^, ^76^, ^78^, ^79^, ^80^, ^81^, ^84^, ^89^, ^95^, ^96^, ^97^, ^98^, ^106^, ^109^, ^110^, ^111^, ^112^, ^113^ by self-report in 13 (17%),^14^, ^15^, ^16^, ^17^, ^18^, ^59^, ^63^, ^65^, ^99^, ^100^, ^101^, ^103^, ^104^ a mixture of medical record or registry and self-report in 26 (31%),^15^, ^16^, ^17^, ^18^, ^44^, ^56^, ^58^, ^61^, ^63^, ^64^, ^74^, ^76^, ^77^, ^82^, ^83^, ^86^, ^90^, ^99^, ^100^, ^101^, ^102^, ^103^, ^104^, ^109^, ^115^ and the method of confirmation was not specified in three (4.8%).^53^, ^62^, ^94^ Patients were classified as vaccinated if they were vaccinated at least 14 days before symptom onset in all studies. Trivalent inactivated vaccines were used in 50 (60%) of 83 studies; however, many studies did not provide information about the specific type of vaccines administered or available.

Among the 83 studies included in the systematic review, 20 (24%) studies provided a total of 27 vaccine effectiveness estimates against generalised influenza for one previous season.^15^, ^16^, ^17^, ^18^, ^41^, ^43^, ^44^, ^45^, ^47^, ^51^, ^54^, ^57^, ^58^, ^63^, ^65^, ^66^, ^75^, ^76^, ^78^, ^79^, ^80^, ^81^, ^82^, ^83^, ^84^, ^85^, ^86^, ^87^, ^88^, ^89^, ^90^, ^99^, ^100^, ^108^, ^109^ 14 estimates^15^, ^66^, ^67^, ^78^, ^86^, ^95^, ^96^, ^97^, ^98^, ^105^, ^111^ showed better vaccine effectiveness for vaccination in the current season only, 12^15^, ^64^, ^68^, ^72^, ^84^, ^87^, ^97^, ^98^, ^104^, ^108^, ^112^ favoured vaccinations in the current and previous seasons, and one^42^ favoured the previous season only.

Four (5%) of 83 studies^45^, ^46^, ^60^, ^71^ compared vaccine effectiveness for various severe outcomes: intensive care unit admission,^45^, ^46^, ^60^ death in hospital^60^ or within 30 days of admission,^45^, ^46^ influenza-associated severe acute respiratory infection,^60^ and hospitalisation of people with diabetes.^71^ The majority of estimates indicated a large increase in vaccine effectiveness for people vaccinated in the current and previous seasons compared with those vaccinated in the current season only.

Ten (12%) of 83 studies^16^, ^17^, ^18^, ^48^, ^74^, ^75^, ^76^, ^88^, ^93^, ^94^ provided vaccine effectiveness estimates with a history covering two or more previous seasons, seven of which^16^, ^17^, ^18^, ^48^, ^74^, ^88^, ^94^ provided 15 estimates for one and two previous vaccinations (appendix p 40). Overall, there appeared to be improved vaccine effectiveness for people vaccinated in the current and one previous season and decreased vaccine effectiveness for those vaccinated in the current and two previous seasons, with 12 (80%) of 15 estimates showing lower VE point estimates in the group vaccinated in two prior seasons. However, results were inconsistent, and increased vaccine effectiveness with an increased number of previous vaccinations was observed for three (20%) of 15 estimates, even for influenza A(H3N2). Multiple previous season vaccine effectiveness was also studied across three,^46^, ^51^, ^70^, ^74^, ^96^ four,^49^, ^58^ five,^22^, ^50^, ^66^, ^70^, ^71^, ^80^ six,^69^ and ten previous seasons.^66^ Generally, we identified no consensus trend of vaccine effectiveness with increasing vaccinations in previous seasons.

Two (2%) of 83 studies^95^, ^96^ reported vaccine effectiveness among patients with and without medically-attended influenza A infection in the previous season. In one study, vaccine effectiveness for the groups vaccinated in the current season and both the current and previous seasons was higher for the group with documented influenza A in the previous season compared with the group with no documented influenza A infection in the previous season.^95^ The other study conducted a sensitivity analysis comparing their main findings with estimates obtained when the sample was restricted to patients with no documented influenza infection in the previous season. They observed a drop in vaccine effectiveness for the group vaccinated in the current and one previous season when the analysis was restricted to patients with no documented influenza A in the previous season.^96^ For influenza B, however, estimates were comparable with their main findings when the analysis was restricted to those patients with no documented influenza B infection in the previous season.^96^

**Figure 2:**
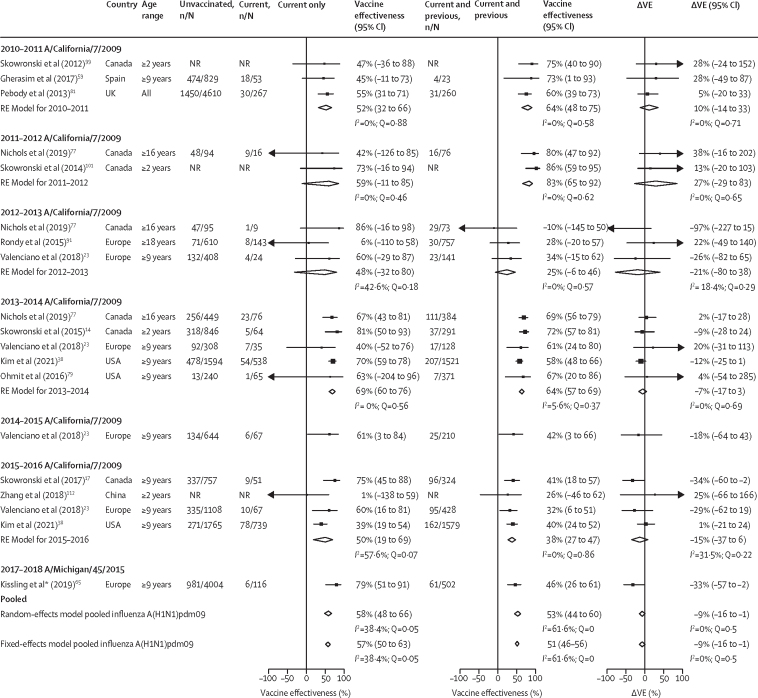
Pooled vaccine effectiveness estimates against influenza A(H1N1)pdm09 for people vaccinated in the current season only, and current and previous seasons, and the difference in these estimates The reference group is people vaccinated in neither season. The previous season is defined as the influenza season immediately before the current season. The absolute difference in vaccine effectiveness (ΔVE) for people who were vaccinated in the current and previous (VE_CP_) seasons and those vaccinated in the current season only (VE_C_) was calculated as: ΔVE = VE_CP_– VE_C_. Random-effect models for each vaccination group are presented pooled by current season and across all seasons; see appendix (p 42) for fixed-effect estimates. Fixed-effect models are only presented for pooled estimates across all seasons; for season-specific fixed-effect estimates see appendix (p 42). NR=not reported. RE model=random-effect model estimate. ΔVE=change in vaccine effectiveness.

**Figure 3:**
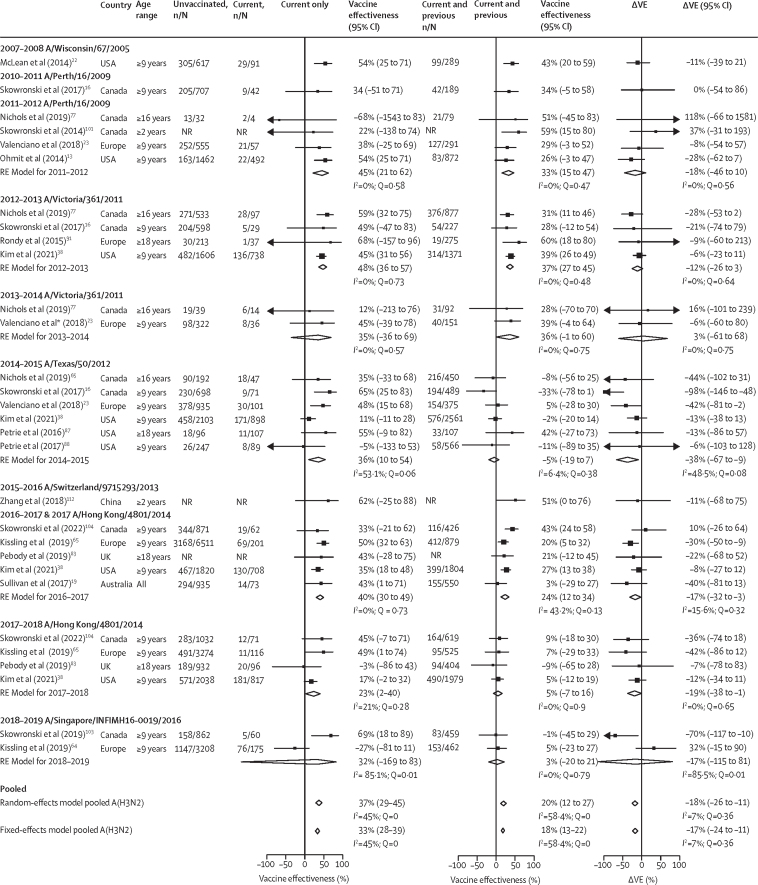
Pooled vaccine effectiveness estimates against influenza A(H3N2) for people vaccinated in the current season only, and current and previous seasons, and the difference in these estimates The reference group is people vaccinated in neither season. The previous season is defined as the influenza season immediately before the current season. The absolute difference in vaccine effectiveness (ΔVE) for people who were vaccinated in the current and previous (VE_CP_) seasons and for those vaccinated in the current season only (VE_C_) was calculated as: ΔVE = VE_CP_– VE_C_. Random-effect models for each vaccination group are presented pooled by current season and across all seasons. Fixed-effect models are only presented for pooled estimates across all seasons; for season-specific fixed-effect estimates see appendix (p 46). NR=not reported. RE model=random-effect model estimate. ΔVE=change in vaccine effectiveness. *Unadjusted vaccine effectiveness estimates are only presented in study.

**Figure 4:**
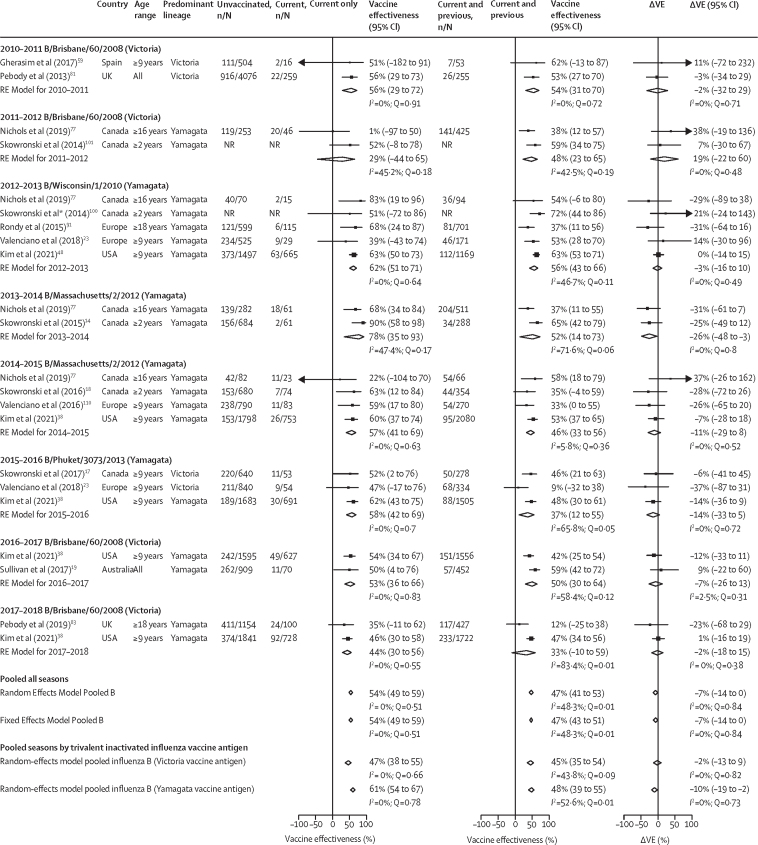
Pooled vaccine effectiveness estimates against influenza B of any lineage for people vaccinated in the current season only, and current and previous seasons, and the difference in these estimates The reference group is people vaccinated in neither season. The previous season is defined as the influenza season immediately before the current season. The absolute difference in vaccine effectiveness (ΔVE) for people who were vaccinated in the current and previous (VE_CP_) seasons and for those vaccinated in the current season only (VE_C_) was calculated as: ΔVE = VE_CP_– VE_C_. Random-effect models for each vaccination group are presented pooled by current season, across all seasons, and by seasons by influenza B antigen included in the trivalent influenza vaccines. Fixed-effect models are presented for pooled estimates across all seasons; for season-specific fixed-effect estimates see appendix (p 50). NR=not reported. RE model=random-effect model estimate. ΔVE=change in vaccine effectiveness.

41 (49%) of 83 studies were eligible for inclusion in the meta-analysis (appendix pp 10–12). These studies reported a total of 85 type-specific or subtype-specific all-age estimates: 19 (23%) for influenza A(H1N1)pdm09, 30 (36%) for influenza A(H3N2), 22 (26%) for influenza B (lineage not specified), five (4%) for influenza B/Victoria, and nine (11%) for influenza B/Yamagata (Figure 2, Figure 3, Figure 4 and table 2). Additionally, 28 estimates were age specific for children, adults, or older adults, in a ratio of 5:1:3 for influenza A(H1N1)pdm09, 7:1:2 for influenza A(H3N2), 2:2:3 for influenza B, and 1:1:0 for influenza B/Yamagata. No age-specific estimates were identified for influenza B/Victoria.

**Table 2:** Summary of meta-analyses* of vaccine effectiveness for two consecutive seasons' vaccination history and influenza type or subtype

	**Current season**	**Previous season**	**Current and previous season**	**ΔVE_c_**†	**ΔVE_p_**†
A(H1N1)pdm09	58% (48 to 66)	33% (21 to 43)	53% (44 to 60)	−9% (−16 to −1)	21% (11 to 30)
A(H3N2)	37% (29 to 45%)	9% (−3 to 19)	20% (12 to 27)	−18% (−26 to −11)	7% (−4 to 18)
B	54% (49 to 59)	21% (12 to 29)	47% (41 to 53)	−7% (−14 to 0)	25% (16 to 34)
B infection and B/Victoria in TIV	47% (38 to 55)	19% (6 to 31)	45% (35 to 54)	−2% (−13 to 9)	26% (11 to 40)
B infection and B/Yamagata in TIV	61% (54 to 67)	23% (11 to 34)	48% (39 to 55)	−10% (−19 to −2)	24% (12 to 37)
B-Victoria infection	61% (43 to 73)	31% (0 to 53)	52% (38 to 63)	−10% (−31 to 12)	15% (−10 to 41)
B-Yamagata infection	56% (39 to 68)	38% (25 to 49)	52% (42 to 60)	−5% (−17 to 6)	14 (0 to 28)

Data are vaccine effectiveness estimates (95% CI). TIV=trivalent influenza vaccine. VE=vaccine effectiveness.

* Random effect model results shown; for fixed effect estimates refer to the appendix (pp 41–55).

† ΔVE>0 implies higher vaccine effective estimate when vaccinated in current and previous seasons compared with the current (VE_C_) or previous (VE_P_) season only.

Estimates for influenza A(H1N1)pdm09 were reported for seven seasons (figure 2 and appendix pp 42–43). Across seasons, vaccine effectiveness was lower for people vaccinated in both the current and previous season (overall ΔVE –9% [95% CI –16 to –1]) than for those vaccinated for the current season only; however, in 2010–11 and 2011–12, ∆VE was positive (10% [–14 to 33] and 27% [–29 to 83]). Few age group-specific vaccine effectiveness estimates were available. ΔVE was calculable for children and older adults and was close to the null for both these age groups (children ΔVE 1% [–18 to 21]; older adults ΔVE 6% [–14 to 26]; appendix pp 44–45).

Vaccine effectiveness estimates for influenza A(H3N2) were available for ten seasons but estimates could only be pooled for 7 seasons (figure 3 and appendix pp 46–47). Pooled vaccine effectiveness estimates for influenza A(H3N2) were low compared with other types, A subtypes or B lineages. Pooled vaccine effectiveness was 37% (95% CI 29–45) for the current season only group, 20% (12–27; ∆VE –18% [–26 to –11]) for the current and previous season group, and 9% (–3 to 19) for the previous season only group. Season-specific pooled vaccine effectiveness estimates for the current and previous season group were lower than for the current season only group (ΔVE >0%), except in 2013–14. The lower season-specific pooled vaccine effectiveness estimates for the current and previous season group were most pronounced in 2014–15 (ΔVE –38% [–67 to –9]). A small number of age-specific estimates were available to pool (appendix pp 48–49), which suggested vaccine effectiveness was better for children vaccinated in the current and previous seasons than for those vaccinated in the current season only, but worse for older adults. However, these estimates should be interpreted with caution as heterogeneity was moderate to high, random-effects and fixed-effects estimates yielded divergent results (see the appendix p 48), and 95% CIs were wide and crossed the null, indicating inconsistent effects.

Most studies providing estimates for influenza B did not estimate vaccine effectiveness by lineage. Across eight seasons, irrespective of the infecting lineage and the lineage included in the vaccine, vaccine effectiveness was minimally reduced for people vaccinated in consecutive seasons compared with those vaccinated in the current season only (ΔVE –7% [95% CI –14 to 0]; figure 4; appendix pp 50–51). In seasons when trivalent vaccines included an influenza B/Yamagata lineage antigen, there was a greater drop in vaccine effectiveness for the current and previous season vaccination group (–10% [–19 to –2) compared with when an influenza B/Victoria lineage antigen was in the vaccine (–2% [–13 to 9]). However, the infecting virus was often not lineage-matched to the vaccine. Season-specific pooled ΔVE estimates favoured current and previous season vaccination for the 2011–12 seasons when the influenza B(Brisbane/60/2008)/Victoria lineage was the vaccine antigen but influenza B/Yamagata viruses dominated. Few estimates were available for age group analysis and pooled age-stratified estimates suggested no differences in effect between children, adults, and older adults (ΔVE near the null; appendix pp 54–55).

Nine North American studies provided estimates against influenza B/Victoria and influenza B/Yamagata infection (appendix pp 52–53). Pooled estimates for each lineage suggested that the net reduction in vaccine effectiveness among those vaccinated in the current and previous seasons compared with those vaccinated in the current season only was greater for influenza B/Victoria viruses (ΔVE –10% [95% CI –31 to 12]) than for influenza B/Yamagata viruses (–5% [–17 to 6]). However, the circulating lineage was mismatched in many seasons, with inconsistent trends across seasons.

Most studies included in the meta-analysis were judged to be at moderate risk of bias (all-age analyses: 23 [77%] studies; age-specific analyses: 12 [75%] studies; Table 1, appendix pp 56). Seven (23%) studies included in the all-age analyses^16^, ^19^, ^77^, ^93^, ^79^, ^88^, ^102^ and four (25%) studies in age-group analyses^57^, ^74^, ^79^, ^88^ were judged to be at a serious risk of bias. The main sources of bias were potential confounding, bias in the classification of interventions due to self-reported vaccination status, and bias due to missing data (appendix p 56).

Within seasons, heterogeneity was generally small. However, there were some exceptions when estimates from the different studies within a season provided divergent results leading to high *I*^2^ and discrepancies in random-effect and fixed-effect estimates (appendix p 42, 46). Across seasons, there was generally moderate heterogeneity for all types, A subtypes and B lineages, highlighting seasonal variations in vaccine effectiveness as vaccination formulation and circulating strains change. For influenza A(H3N2), there was also evidence of heterogeneity when summarising across age groups (appendix pp 48–49). Evidence of publication bias was apparent for influenza A(H3N2) estimates for the current and previous season vaccination group (p=0·03; appendix pp 57–58).

None of the sensitivity analyses revealed a concerning change in estimates (appendix pp 59–63).

Using GRADE analysis, we assessed the certainty of evidence at baseline as low, owing to the observational study designs. The certainty for attenuation of vaccine effectiveness for influenza A(H1N1)pdm09 and influenza B remained low with subsequent vaccinations; that is, repeat vaccination might reduce vaccine effectiveness (appendix pp 64–68). For influenza A(H3N2), the certainty of evidence was downgraded to very low due to concerns about imprecision (ie, repeat vaccination might have reduced vaccine effectiveness, but the certainty of evidence was very low).

## Discussion

This systematic review of 83 studies did not identify sufficient evidence to warrant a change in annual influenza vaccination recommendations. The meta-analysis of 41 studies observed an average reduction in vaccine effectiveness for people vaccinated in the current and previous seasons compared with the current only season (ΔVE) of –9% (95% CI –16 to –1) for influenza A(H1N1)pdm09, –18% (–26 to –11) for influenza A(H3N2), and –7 (–14 to 0) for influenza B. These estimates suggest that there might be some attenuation of vaccine effectiveness with successive revaccination. However, in most seasons, vaccine effectiveness for the group vaccinated in the current and previous season was positive (vaccine effectiveness >0%) and was higher than vaccine effectiveness for the people vaccinated in the previous season only, indicating that vaccination in successive seasons offers better protection against influenza illness than no vaccination.

Estimates for the effects of repeated vaccination for influenza A(H1N1)pdm09 immediately after its pandemic appeared to indicate an additive benefit of consecutive vaccination for these viruses and lingering benefits to vaccination in a previous season, an observation shared with previous reviews.^32^, ^33^ “In the two seasons immediately following the influenza A(H1N1)pdm09 pandemic, a higher proportion of patients would have been recently infected (infected within the previous two seasons) than during seasons not immediately following a pandemic. Vaccination might have boosted infection-acquired antibodies and conferred a high degree of protection in people who were vaccinated. This idea was supported by observations that vaccine effectiveness, even in people who had repeated vaccination, was higher in those with recent infection than in those without recent infection.^11^, ^95^, ^96^ Moreover, influenza pandemic vaccine formulations in 2009–10 were adjuvanted and might have stimulated a broader immune response that continued to provide protection during the viruses' initial evolution. They were also initially monovalent so only stimulated antibody responses to one antigen, rather than three. Finally, the vaccination history in these seasons soon after the pandemic represent a cleaner vaccination history as they could not have received vaccination prior to 2009.

Low vaccine effectiveness against influenza A(H1N1)pdm09 in the 2013–14 season has been linked to a change in an epitope conserved between older seasonal influenza A(H1N1) and influenza A(H1N1)pdm09 viruses, on which antibodies were focused.^29^, ^30^, ^116^ Some evidence suggests these effects are most pronounced in adult individuals born between 1965 and 1979.^116^ However, in our study there were insufficient estimates available to assess whether the effects of repeated vaccination might vary with age. Since 2013–14, influenza A(H1N1)pdm09 viruses have continued to evolve and have diversified into cocirculating antigenically-distinct groups. This diversity increases the possibility that emerging circulating viruses will be antigenically distinct from the selected vaccine antigen. The degree to which ∆VE continues to decrease for influenza A(H1N1)pdm09 might not become evident for several more years.

As expected, vaccine effectiveness against influenza A(H3N2) viruses appeared to be reduced most by repeated vaccination. ΔVE estimates for influenza A(H3N2) viruses were the largest observed among types, A subtypes and B lineages, compounded by overall low vaccine effectiveness (mostly <40%). Nevertheless, in most seasons, vaccination in consecutive seasons afforded some protection and this protection exceeded the protection afforded by vaccination in the previous season only. In seasons when repeated vaccination appeared to be helpful, data were sparse and imprecise and the influence of those seasons on overall estimates was limited.

Multiple factors might contribute to poor vaccine effectiveness against influenza A(H3N2). First, there is greater heterogeneity among circulating influenza A(H3N2) viruses than other influenza viruses, making it difficult to select antigens capable of eliciting a broad antibody response.^117^ Second, influenza viruses might acquire adaptations that enable growth in eggs but alter antigenicity.^31^ Most vaccination studies involve inactivated egg-grown virus vaccines, the effectiveness of which has been compromised in seasons when adaptations in the egg have affected key antigenic or glycosylation sites.^100^ Inactivated cell-grown and recombinant haemagglutinin might be more immunogenic^118^, ^119^, ^120^ and effective than egg-grown vaccines.^121^, ^122^, ^123^ However, there were insufficient data available in our systematic review and meta-analysis to assess whether the growth substrate can overcome or alleviate the attenuating effects of repeated vaccination.

Immunological studies have shown that antibody responses to influenza A(H3N2) can be blunted with each additional vaccination received.^8^, ^10^ How this decrease in antibody response translates to vaccine effectiveness is unclear and the studies providing such information observed no consistent losses in vaccine effectiveness with successive years of vaccination, consistent with our expectations that these effects vary annually.^24^ Antibody responses might increase when encountering an antigenically distinct vaccine antigen,^11^ but infection appears to be more immunogenic than vaccination^124^ and might mitigate the negative effects of previous vaccination.^11^, ^95^ However, permitting infection to improve vaccine responses is counterintuitive. Similarly, biannual vaccination has been proposed as a potential measure to mitigate repeat vaccination effects, and one modelling study suggests this would provide comparable protection to annual vaccination in repeat vaccinees.^125^ However, such a programme might be logistically challenging. Continued and bolstered support for surveillance systems and research is needed to investigate these scenarios of successive vaccination, interrupted vaccination, and infection versus vaccination to help guide policy decision making.

Our analysis of influenza A(H3N2) vaccine effectiveness by age group was also hampered by insufficient data. Importantly, the data available represented a small number of seasons and should be interpreted with caution because of strong seasonal effects and heterogeneity among seasons. Although attenuation of vaccine effectiveness might be more pronounced in older age groups than in younger age groups, we cannot be certain of this on the basis of the available data. Some of the reduced vaccine effectiveness in older age groups might be associated with immunosenescence. However, modelling studies have suggested that poor vaccine effectiveness in older age groups is better explained by repeated vaccination than with age-associated immunosenescence.^126^ More data are needed before drawing any conclusions about the age-specific effects of repeated influenza A(H3N2) vaccination.

For influenza B, the reduction in vaccine effectiveness associated with repeated vaccination was small. Understanding these effects for influenza B is complicated by lineage-mismatched trivalent inactivated influenza vaccine and interseasonal variations in the dominant lineage. Disentangling the effects of repeated vaccination for influenza B lineages will be enabled by increased use of quadrivalent vaccines. However, circulation of influenza B/Yamagata lineage viruses has not been confirmed since early 2020,^127^ so ongoing monitoring of this lineage might become irrelevant.

A key observation for all types, A subtypes or B lineages of influenza was evidence of heterogeneity between, but not necessarily within, seasons. In some seasons, the *I*^2^ statistic might not have been a reliable indicator of heterogeneity because most estimates were imprecise and consistency of effect might be more important for interpretation. Nevertheless, interseasonal variability in estimates reinforces hypotheses that the effects of repeated vaccination are not expected to be evident every year and are influenced by interseasonal variations in vaccine formulations, dominant circulating viruses, emergence of antigenically drifted variants, antigenic similarity between vaccine and circulating antigens, and the overall population susceptibility. Pooling data across seasons hides some of these problems but does not indicate the absence of a problem.

Our systematic review and meta-analysis followed PRISMA guidelines (see appendix (pp 2-4) for PRISMA checklist) and registered our protocol with Prospero. We incorporated a risk of bias analysis and used GRADE to evaluate the weight of the available information. We conducted extensive sensitivity analyses to assess the robustness of our conclusions and have highlighted where the data provide inconclusive evidence. Nevertheless, our study was hampered by low availability of estimates for subgroup assessments, especially for age group. Given the importance of repeated vaccine effectiveness for vaccination policy, it is recommended that studies attempting to estimate seasonal influenza vaccine effectiveness routinely collect information on previous season's vaccination status and be sufficiently powered for subgroup estimation. In addition, many studies had few patients in the current season vaccination only and previous season vaccination only groups, leading to estimates with high uncertainty and, in some cases, vaccine effectiveness could not be estimated. In these cases, we elected to include all available estimates even if some vaccine group estimates were unavailable for a particular study, preferring to use all available information than to exclude a study. Bolstered support for vaccine effectiveness studies to ensure adequate numbers of patients in all vaccination groups is necessary to improve the quality of estimates to inform vaccine policy decision making.

In conclusion, the data available currently suggest the vaccine effectiveness for people vaccinated in both the current and previous season is, on average, comparable with vaccine effectiveness for people vaccinated in the current season only and better than vaccine effectiveness for people vaccinated in the previous season only. Vaccine effectiveness against influenza A(H3N2) is worse overall and there is a greater loss of effectiveness with repeated vaccination. Quantifying the relative vaccine effectiveness for people vaccinated in consecutive seasons is difficult if vaccine effectiveness is low and points to the general need for better vaccines against these viruses in particular and against influenza in general.^128^ Although there will be seasons when the effects of repeated vaccination are more pronounced than others, the current evidence does not suggest there is a consistent and severe enough attenuation to recommend any changes to annual vaccination recommendations.

## Data sharing

Extracted data are available from the corresponding author on request.

## Declaration of interests

SGS and AF report the following funding for influenza vaccination and infection studies: US National Institutes of Health (SGS and AF); US Centers for Disease Control (SGS and AF); the National Health and Medical Research Council of Australia (AF); and OptumLabs research credits (SGS). SGS and AF are employed by the WHO Collaborating Centre for Reference and Research on Influenza, which receives funding from the International Federation of Pharmaceutical Manufacturers and Associations and Seqiris for the development of influenza vaccines. SGS has served in an unpaid capacity on advisory boards for Sanofi and Seqiris. From 2017–21, SGS was a member of the WHO Strategic Advisory Group of Experts (SAGE) on Immunization Working Group on Influenza. SGS serves on the Australian National Influenza Surveillance Committee. EJ-G, EJR, and AJK declare no competing interests.
