# Supplementary material for: Does repeated influenza vaccination attenuate effectiveness? A systematic review and meta-analysis
Source: Lancet Respir Med. 2023 Jan;11(1):27–44. doi: 10.1016/S2213-2600(22)00266-1 (PMC9780123; doi:10.1016/S2213-2600(22)00266-1)
Supplement: Supplementary appendix [file mmc1.pdf]

THE LANCET
Respiratory Medicine

Supplementary appendix

This appendix formed part of the original submission and has been peer reviewed. We post it as supplied by the authors.

Supplement to: Jones-Gray E, Robinson EJ, Kucharski AJ, Fox A, Sullivan SG. Does repeated influenza vaccination attenuate effectiveness? A systematic review and meta-analysis. *Lancet Respir Med* 2022; published online Sept 21. https://doi.org/10.1016/S2213-2600(22)00266-1.

Does repeated influenza vaccination attenuate effectiveness? A systematic review and meta-analysis

Supplementary material

Jones-Gray E, Robinson EJ, Kucharski AJ, Fox A, Sullivan SG

September 13, 2022

Contents

1 PRISMA checklist 2

2 Database search terms 5

3 Data collection form 6

4 R script for calculating Delta VE 8

5 Study list and inclusion documentation 10

6 Summary of study designs 15

7 Multiple prior year history 40

8 Meta-analysis - additional data 41

9 Risk of bias 56

10 Publication bias 57

11 Sensitivity analyses 59

12 GRADE evaluation 64

13 References 69

1 PRISMA checklist

Section and Topic	Item #	Checklist item	Location where item is reported
**TITLE**			
Title	1	Identify the report as a systematic review.	P1
**ABSTRACT**			
Abstract	2	See the PRISMA 2020 for Abstracts checklist.	P1
**INTRODUCTION**			
Rationale	3	Describe the rationale for the review in the context of existing knowledge.	P1-2
Objectives	4	Provide an explicit statement of the objective(s) or question(s) the review addresses.	P2
**METHODS**			
Eligibility criteria	5	Specify the inclusion and exclusion criteria for the review and how studies were grouped for the syntheses.	Supp Part 2&5
Information sources	6	Specify all databases, registers, websites, organisations, reference lists and other sources searched or consulted to identify studies. Specify the date when each source was last searched or consulted.	P3, Supp Part 2
Search strategy	7	Present the full search strategies for all databases, registers and websites, including any filters and limits used.	P8, Supp Part 2
Selection process	8	Specify the methods used to decide whether a study met the inclusion criteria of the review, including how many reviewers screened each record and each report retrieved, whether they worked independently, and if applicable, details of automation tools used in the process.	P3
Data collection process	9	Specify the methods used to collect data from reports, including how many reviewers collected data from each report, whether they worked independently, any processes for obtaining or confirming data from study investigators, and if applicable, details of automation tools used in the process.	P3
Data items	10a	List and define all outcomes for which data were sought. Specify whether all results that were compatible with each outcome domain in each study were sought (e.g. for all measures, time points, analyses), and if not, the methods used to decide which results to collect.	P3, Supp Part 3
	10b	List and define all other variables for which data were sought (e.g. participant and intervention characteristics, funding sources). Describe any assumptions made about any missing or unclear information.	P3, Supp Part 3
Study risk of bias assessment	11	Specify the methods used to assess risk of bias in the included studies, including details of the tool(s) used, how many reviewers assessed each study and whether they worked independently, and if applicable, details of automation tools used in the process.	P3, Supp Part 9
Effect measures	12	Specify for each outcome the effect measure(s) (e.g. risk ratio, mean difference) used in the synthesis or presentation of results.	P3, Supp Part 3
Synthesis methods	13a	Describe the processes used to decide which studies were eligible for each synthesis (e.g. tabulating the study intervention characteristics and comparing against the planned groups for each synthesis (item #5)).	P3, Supp Part 5
	13b	Describe any methods required to prepare the data for presentation or synthesis, such as handling of missing summary statistics, or data conversions.	P3, Supp Part 5
	13c	Describe any methods used to tabulate or visually display results of individual studies and syntheses.	P3
	13d	Describe any methods used to synthesize results and provide a rationale for the choice(s). If meta-analysis was performed, describe the model(s), method(s) to identify the presence and extent of statistical heterogeneity, and software package(s) used.	P3
	13e	Describe any methods used to explore possible causes of heterogeneity among study results (e.g. subgroup analysis, meta-regression).	P3
	13f	Describe any sensitivity analyses conducted to assess robustness of the synthesized results.	P3, Supp Part

Section and Topic	Item #	Checklist item	Location where item is reported
			10
Reporting bias assessment	14	Describe any methods used to assess risk of bias due to missing results in a synthesis (arising from reporting biases).	P14, Supp Part 9
Certainty assessment	15	Describe any methods used to assess certainty (or confidence) in the body of evidence for an outcome.	P9, Supp Part 11
**RESULTS**			
Study selection	16a	Describe the results of the search and selection process, from the number of records identified in the search to the number of studies included in the review, ideally using a flow diagram.	P10, Fig 1, Supp Part 4
	16b	Cite studies that might appear to meet the inclusion criteria, but which were excluded, and explain why they were excluded.	Table 1, Supp Part 4
Study characteristics	17	Cite each included study and present its characteristics.	P1—11, Table 1, Supp Part 5
Risk of bias in studies	18	Present assessments of risk of bias for each included study.	P13, Table 1, Supp Part 8
Results of individual studies	19	For all outcomes, present, for each study: (a) summary statistics for each group (where appropriate) and (b) an effect estimate and its precision (e.g. confidence/credible interval), ideally using structured tables or plots.	P12-13, Table 2, Figs 2-5; Supp Part 7
Results of syntheses	20a	For each synthesis, briefly summarise the characteristics and risk of bias among contributing studies.	P13, Supp Part 8
	20b	Present results of all statistical syntheses conducted. If meta-analysis was done, present for each the summary estimate and its precision (e.g. confidence/credible interval) and measures of statistical heterogeneity. If comparing groups, describe the direction of the effect.	P12-13, Table 2, Figs 2-5; Supp Part 7
	20c	Present results of all investigations of possible causes of heterogeneity among study results.	Figs 2-5, Supp Part 7
	20d	Present results of all sensitivity analyses conducted to assess the robustness of the synthesized results.	P14, Supp Part 10
Reporting biases	21	Present assessments of risk of bias due to missing results (arising from reporting biases) for each synthesis assessed.	Supp Part 10
Certainty of evidence	22	Present assessments of certainty (or confidence) in the body of evidence for each outcome assessed.	P14, Supp Part 11
**DISCUSSION**			
Discussion	23a	Provide a general interpretation of the results in the context of other evidence.	P14-17
	23b	Discuss any limitations of the evidence included in the review.	P14-17
	23c	Discuss any limitations of the review processes used.	P17
	23d	Discuss implications of the results for practice, policy, and future research.	P17
**OTHER INFORMATION**			

Section and Topic	Item #	Checklist item	Location where item is reported
Registration and protocol	24a	Provide registration information for the review, including register name and registration number, or state that the review was not registered.	P7
	24b	Indicate where the review protocol can be accessed, or state that a protocol was not prepared.	P7
	24c	Describe and explain any amendments to information provided at registration or in the protocol.	NA
Support	25	Describe sources of financial or non-financial support for the review, and the role of the funders or sponsors in the review.	P10, 19, Author declaration form
Competing interests	26	Declare any competing interests of review authors.	P18, COI forms
Availability of data, code and other materials	27	Report which of the following are publicly available and where they can be found: template data collection forms; data extracted from included studies; data used for all analyses; analytic code; any other materials used in the review.	P18, Supp Parts 3 & 6

From: Page MJ, McKenzie JE, Bossuyt PM, Boutron I, Hoffmann TC, Mulrow CD, et al. The PRISMA 2020 statement: an updated guideline for reporting systematic reviews. BMJ 2021;372:n71. doi: 10.1136/bmj.n71

For more information, visit: http://www.prisma-statement.org/

2 Database search terms

Database search terms used in Medline, EMBASE, CINAHL Complete, Web of Science.

2.1 Ovid – Medline, EMBASE

Inclusion terms Influenza, Human/ or exp Influenza A Virus/ or exp Influenza B virus/ or Influenza Vaccines/ or ((flu or influenza).ti,kw,kf. and ("in data review" or in process or "pubmed not medline").st.)

Vaccines/ or Viral Vaccines/ or Vaccines, Attenuated/ or Vaccines, Inactivated/ or Vaccination/ or Mass Vaccination/ or Immunization/ or Immunization, Secondary/ or Immunization Programs/ or Influenza Vaccines/ or ((vaccin* or revaccinat* or immunis* or immuniz* or reimmunis* or reimmuniz*).ti,kw,kf. and ("in data review" or in process or "pubmed not medline").st.)

Treatment outcome/ or (effectiveness or impact or efficac* or protection or protective or performance).ti,kw,kf. or ((vaccin* or revaccinat* or immunis* or immuniz* or reimmunis* or reimmuniz*) adj3 (effectiveness or impact or efficac* or protection or protective or performance)).ab. or "vaccine effectiveness".mp.

Exclusion terms "cost effectiveness".tw. or (exp Animals/ not Humans/) or Animals/ or (("non human" or primate* or mouse or mice or macaque* or ferret* or animal* or bird* or poultry or chicken* or swine or pig* or duck*).ti. and ("in data review" or in process or "pubmed not medline").st.) or ("meta analysis").pt.

Re-run of search on 13 June 2022 with the following additional exclusion terms (SARS-CoV* or covid* or "coronavirus").tw.

2.2 CINAHL Complete

(Influenza or flu) and (vaccines or vaccinations or immunizations or immunisation) and (effectiveness or efficacy or effective) not ("cost effectiveness" or "meta-analysis")TI not (animal* or mouse or mice or macaque* or ferret* or bird* or poultry or chicken* or swine or pig* or duck*)TI

Re-run of search on 13 June 2022 with the following additional exclusion terms (covid-19 or coronavirus or 2019-ncov or sars-cov-2 or cov-19)TI

2.3 Web of Science

TI=(Influenza OR flu) AND TI=(vaccines or vaccinations or immunizations or immunisation) AND TI=(effectiveness or efficacy or effective) AND AB=(Influenza OR flu) AND AB = (vaccines or vaccinations or immunizations or immunisation) AND AB=(effectiveness or efficacy or effective) NOT TI = ("cost effectiveness" or "meta-analysis") NOT AB=(animal* or mouse or mice or macaque* or ferret* or bird* or poultry or chicken* or swine or pig* or duck*) OR TI=(animal* or mouse or mice or macaque* or ferret* or bird* or poultry or chicken* or swine or pig* or duck*)

3 Data collection form

**Identifiers**	
Reference number	
First author, year	
Author contact details	
Study title	
Supplemental material	☐01 Yes ☐02 No
Errata	☐01 Yes ☐02 No

**Eligibility**	
Case definition	☐01 Case = laboratory confirmed influenza<br>Additional information:<br><br>☐02 Control = laboratory result negative for influenza<br>Additional information:<br><br>☐03 Vaccinated = vaccination ≥14 days before sample collection / symptom onset *(circle one)*
Publication language	☐01 English ☐02 Other: _______________

**Study Characteristics**	
Study design	☐01 Test negative ☐02 Case-control ☐03 Prospective cohort<br>☐04 Retrospective cohort ☐05 RCT ☐07 Other: ________________
Setting	☐01 Inpatient ☐02 Outpatient ☐03 Community ☐04 Other: ________
# of sites	
Participant recruitment	
Eligibility & Inclusion criteria	
Exclusion criteria	
Hemisphere	☐01 Northern ☐02 Southern
Country	
Seasons covered	
Season start, end	
Current season year	
Prior season/s year/s	
Age demographic	☐01 Children ☐02 Adults ☐03 Elderly ☐04 All ☐05 Other: ___________
Age range	
Type of statistical analysis	☐01 Logistic regression ☐02 Multivariable LR ☐03 Conditional LR<br>☐05 Other: __________________
Vacc. status source	☐01 Self report ☐02 Official record ☐03 Other: _________________
Vaccine type/s	☐01 LAIV ☐02 IIV ☐03 TIV ☐04 QIV ☐05 N/S ☐06 Other: ___________

Influenza subtypes studied	☐01 A overall ☐02 A(H1N1) ☐03 A(H3N2) ☐04 A(un-subtyped)<br>☐05 B overall ☐06 B/_________ ☐07 B/_________ ☐08 Co-infection
Laboratory confirmation	☐01 RT-PCR ☐02 Viral culture ☐03 Antigenic characterisation<br>☐04 Genetic sequencing ☐05 Other: _________________

**Outcomes**	Subtype: _________ Season: __________ Age range: ________			
**VE estimates:** ☐01 VE ☐02 OR				
	# Cases	# Controls	Crude (95% CI)	Adjusted (95% CI)
Current and prior				
Current only				
Prior only				
Unvaccinated both				
Current season vaccine strain				
Prior season vaccine strain				
Main circulating strain				
Vaccine/circulating strain match	☐01 Matched ☐02 Mis-matched ☐03 Other:_______________			
Adjustment variables	☐01 Age ☐02 Sex ☐03 Co-morbidities ☐04 Interval from illness onset to specimen collection ☐05 Season ☐06 Calendar month ☐07 Past season influenza diagnosis ☐08 Site ☐09 Region ☐10 Ethnicity ☐11 Risk group<br>☐12 Economic deprivation<br>☐13 Other: ___________			
Other restrictions				

**Include in meta-analysis**	☐01 Yes<br>☐02 No (give reason):<br>☐02.1 Prior season information is not immediately prior alone<br>☐02.2 Generalized influenza only<br>☐02.3 Subset of severity outcomes only

**Missing data (for RoB)**				
	# Cases	# Controls	Total	Excluded?
Vaccination status				☐01 Y ☐02 N
Vaccination date				☐01 Y ☐02 N
Vaccination type				☐01 Y ☐02 N
Covariates				☐01 Y ☐02 N
Other: __________				☐01 Y ☐02 N
Other: __________				☐01 Y ☐02 N
Other: __________				☐01 Y ☐02 N
Other: __________				☐01 Y ☐02 N

Notes:

4 R script for calculating Delta VE

The R script below shows the calculation of $\Delta VE_{current}$

```
# ============ Set-up ============
library(metafor)
library(readxl)

ma.dat <- read_excel()  # load dataset

# Clean the dataset to include studies included in meta-analysis
# Dataset includes columns:
# Author.PublicationYear
# Country.short
# Age.range
# Current.season
# Virus
# c.ve: current season vaccination only VE
# c.ll: current season vaccination only 95% lower CI
# c.ul: current season vaccination only 95% upper CI
# b.ve: both current and prior season vaccination VE
# b.ll: both current and prior season vaccination 95% lower CI
# b.ul: both current and prior season vaccination 95% upper CI

# ============ Calculate odds ratios (OR) and SE based on VE ============

# Functions to move to log odds scale
## Current season only
logscale.c <- function(x){
  logscale.m <- x # results will be added to the original dataframe
  logscale.m$c.or <- log((100-x$c.ve)/100,base=exp(1))
  logscale.m$c.or.lb <- log((100-x$c.ul)/100,base=exp(1))
  logscale.m$c.or.ub <- log((100-x$c.ll)/100,base=exp(1))
  return(logscale.m)
}
## Current and prior season
logscale.b <- function(x){
  logscale.o <- x # results will be added to the original dataframe
  logscale.o$b.or <- log((100-x$b.ve)/100,base=exp(1))
  logscale.o$b.or.lb <- log((100-x$b.ul)/100,base=exp(1))
  logscale.o$b.or.ub <- log((100-x$b.ll)/100,base=exp(1))
  return(logscale.o)
}
# Functions to convert confidence intervals to standard error
## Current season only
stderror.c <- function(x){
  c.se <- x
  c.se$c.se <- (c.se$c.or.ub - c.se$c.or.lb)/3.92
  return(c.se)
}
## Current and prior seasons
stderror.b <- function(x){
  b.se <- x
  b.se$b.se <- (b.se$b.or.ub - b.se$b.or.lb)/3.92
  return(b.se)
}

# Get odds ratio and standard error using functions (adds to dataframe)
ma.dat <- logscale.c(ma.dat)
ma.dat <- stderror.c(ma.dat)
ma.dat <- logscale.b(ma.dat)
```

```
ma.dat <- stderror.b(ma.dat)

# ============ Calculate deltaVE and CIs ============

ma.dat$delta.ve <- round(ma.dat$b.ve - ma.dat$c.ve, 0)
## deltaVE: VEcurrent and prior - VEcurrent only

set.seed(0725)
n.sample <- 1000
delta.ve.ll <- delta.ve.ul <- rep(NA, n.sample)
# calculate mean and SE for each ??VE for meta-analysis
meta.delta.ve.mean <- meta.delta.ve.se <- rep(NA, n.sample)

# Bootstrap
for(i in 1:nrow(ma.dat)){
  for(j in 1:n.sample){
    b.ve <- (1-exp(rnorm(1000, ma.dat$b.or[i], ma.dat$b.se[i])))*100
    c.ve <- (1-exp(rnorm(1000, ma.dat$c.or[i], ma.dat$c.se[i])))*100
    delta.ve.ll[j] <- quantile(b.ve - c.ve, 0.025, na.rm=TRUE)
    delta.ve.ul[j] <- quantile(b.ve - c.ve, 0.975, na.rm=TRUE)
    meta.delta.ve.mean[j] <- mean(b.ve - c.ve)
    meta.delta.ve.se[j] <- sd(b.ve - c.ve) }
  ma.dat$delta.ve.ll[i] <- round(mean(delta.ve.ll), 0)
  ma.dat$delta.ve.ul[i] <- round(mean(delta.ve.ul), 0)
  ma.dat$meta.delta.ve.mean[i] <- round(mean(meta.delta.ve.mean), 0)
  ma.dat$meta.delta.ve.se[i] <- round(mean(meta.delta.ve.se), 3)
}       # end of loop

madat$delta.ve.ci <- paste(ma.dat$delta.ve, "% (", ma.dat$delta.ve.ll, "%,
                               ", ma.dat$delta.ve.ul, "%)", sep="")
ma.dat$delta.ve.ci

# ============ Meta-analysis ============

# Split by 'Virus' for separate forest plots
# split to list of dataframes
Virus <- split(ma.dat, f = ma.dat$Virus)
# convert list of dataframes to dataframes in environment
# [A(H1N1)pdm09; A(H3N2); B/Overall; B/Victoria; B/Yamagata]
list2env(Virus, envir = .GlobalEnv)

# Meta-analysis model [shown for A(H1N1)pdm09]

Meta.h1 <- list()
Meta.h1$Model <- rma(yi = meta.delta.ve.mean, sei = meta.delta.ve.se,
                     data = `A(H1N1)pdm09`, method="FE")
Meta.h1$delta.ve <- round(c(Meta.h1$Model$b), 0)
Meta.h1$delta.ve.se <- Meta.h1$Model$se
Meta.h1$delta.ve.ll <- round(Meta.h1$delta.ve+qnorm(0.025)*Meta.h1$delta.ve.se,0)
Meta.h1$delta.ve.ul <- round(Meta.h1$delta.ve+qnorm(0.975)*Meta.h1$delta.ve.se,0)
Meta.h1$delta.ve.ci <- paste(Meta.h1$delta.ve, "% (", Meta.h1$delta.ve.ll, "%, ",
                             Meta.h1$delta.ve.ul, "%)", sep="")
Meta.h1$delta.ve.ci

# end
```

5 Study list and inclusion documentation

Supplementary Figure 1: Number of studies by year of publication for 83 studies included in this review

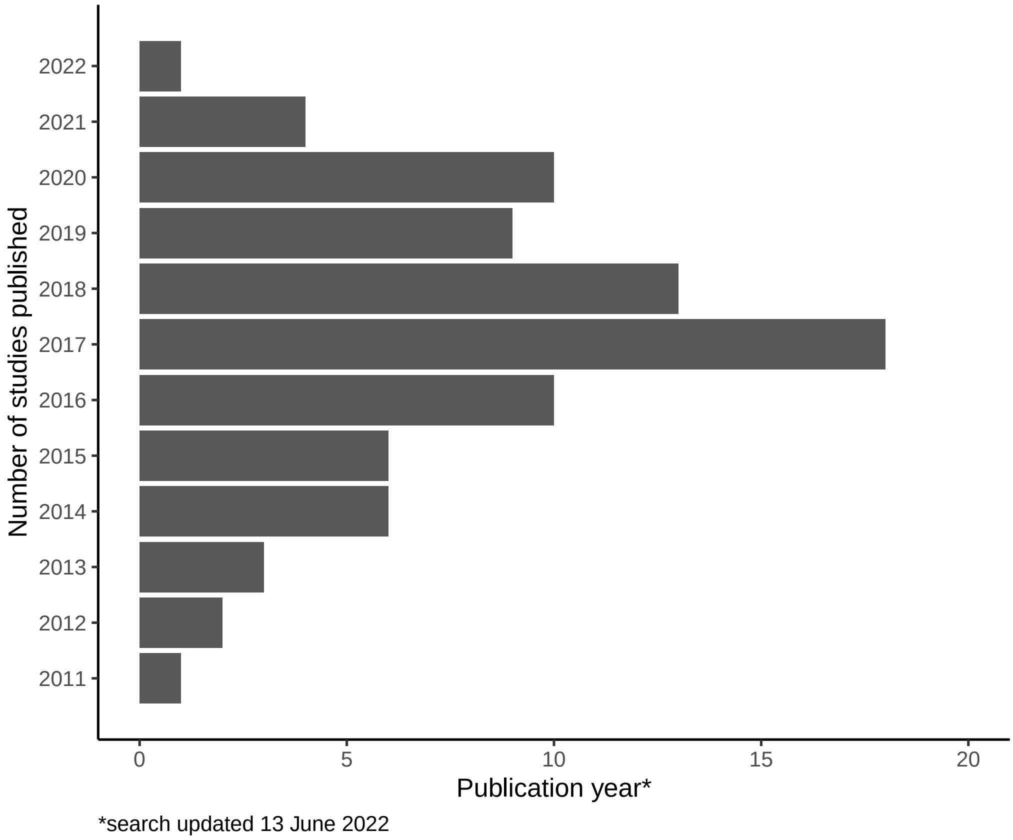

Supplementary Table 1: Study list and inclusion documentation

Covidence No.	Author, year	Status
1720	Boddington, 2019 [1]	Narrative synthesis of generalised influenza
1432	Buchan, 2017 [2]	Narrative synthesis of pooled seasons. Confidence intervals not shown and authors were unable to provide
1049	Buchan, 2018 [3]	Narrative synthesis of pooled seasons and three prior seasons
67	Casado, 2016 [4]	Narrative synthesis of generalised influenza and severe/non-severe comparison
1599	Casado, 2018 [5]	Narrative synthesis of three prior seasons
6935	Castilla, 2011 [6]	Narrative synthesis of generalised influenza
87	Castilla, 2016 [7]	Narrative synthesis of two prior seasons
529	Castilla, 2017 [8]	Narrative synthesis of four prior seasons
843	Castilla, 2018 [9]	Narrative synthesis of inpatient/outpatient comparison and five prior seasons
2119	Castilla, 2020 [10]	Narrative synthesis of three prior seasons
545	Cheng, 2017 [11]	Narrative synthesis of pooled seasons. Confidence intervals not shown and authors did not respond
737	Dominguez, 2017 [12]	Narrative synthesis of generalised influenza only
1490	El Omeiri, 2018 [13]	Included in meta-analysis of H1N1 elderly. Narrative synthesis of generalised influenza
1282	Ferdinands, 2019 [14]	Narrative synthesis of generalised influenza only
1226	Flannery, 2019 [15]	Eligible for meta-analysis but not included due reanalysed data. Narrative synthesis
6933	Fu, 2015 [16]	Included in meta-analysis of H1N1 children

Covidence No.	Author, year	Status
73	Gaglani, 2016 [17]	Included in meta-analysis of H1N1 children, adults and elderly. Narrative synthesis of four prior seasons
5707	Gherasim, 2017 [18]	Included in meta-analysis of H1N1, B
7889	Grijalva, 2021 [19]	Narrative synthesis of estimates against generalised influenza from participants with severe outcomes only
705	Jackson, 2017 [20]	Included in meta-analysis of B/Yam, B/Vic
6932	Jiménez-Jorge, 2012 [21]	Eligible for meta-analysis but not included due reanalysed data. Narrative synthesis
3376	Kim, 2021 [22]	Included in meta-analysis of H3N2, H1N1, B
4834	Kissling, 2018 [23]	Included in meta-analysis of B adults
1840	Kissling, 2019a [24]	Included in meta-analysis of H3N2, and H3N2 adults and older adults
416	Kissling, 2019b [25]	Included in meta-analysis of H3N2, H1N1
1879	Kwong, 2020 [26]	Narrative synthesis of generalised influenza, five and ten prior seasons
735	Ma, 2017 [27]	Narrative synthesis of generalised influenza
6931	Martinez-Baz, 2013 [28]	Eligible for meta-analysis but not included due reanalysed data. Narrative synthesis
603	Martinez-Baz, 2017 [29]	Narrative synthesis of 1-6 prior seasons
7577	Martinez-Baz, 2021a [30]	Narrative synthesis of pooled seasons, three and five prior seasons
8197	Martinez-Baz, 2021b [31]	Narrative synthesis of pooled seasons
6929	McLean, 2014 [32]	Included in meta-analysis of H3N2. Narrative synthesis of pooled seasons and five prior seasons
6930	McLean, 2015 [33]	Included in meta-analysis of B/Yam
591	McLean, 2017 [34]	Eligible for age specific meta-analysis but not included due reanalysed data. Narrative synthesis
1163	McLean, 2018 [35]	Included in meta-analysis of H3N2 children. Narrative synthesis of pooled seasons, two and three prior seasons
850	Mira-Iglesias, 2018 [36]	Narrative synthesis of two prior seasons
1730	Mira-Iglesias, 2019 [37]	Narrative synthesis of two prior seasons
1261	Nichols, 2019 [38]	Included in meta-analysis of H3N2, H1N1, B
6927	Ohmit, 2014 [39]	Included in meta-analysis of H3N2
6928	Ohmit, 2015 [40]	Narrative synthesis of generalised influenza
50	Ohmit, 2016 [41]	Included in meta-analysis of H1N1 and H1N1 children
1058	Ortqvist, 2018 [42]	Narrative synthesis of generalised influenza, four and five prior seasons
6926	Pebody, 2013 [43]	Included in meta-analysis of H1N1, B
1419	Pebody, 2017 [44]	Included in meta-analysis of H3N2, and H3N2 children
1732	Pebody, 2019 [45]	Included in meta-analysis of H3N2, B and H3N2, B children
2056	Pebody, 2020a [46]	Included in meta-analysis of H1N1, H3N2 children
2058	Pebody, 2020b [47]	Included in meta-analysis of H1N1, H3N2 elderly
2067	Pebody, 2020c [48]	Narrative synthesis of generalised influenza
189	Petrie, 2016 [49]	Included in meta-analysis of H3N2
776	Petrie, 2017 [50]	Included in meta-analysis of H3N2, B/Yam, and H3N2, B/Yam children. Narrative synthesis of two prior seasons.
271	Powell, 2020 [51]	Narrative synthesis of generalised influenza
2542	Rao, 2021 [52]	Narrative synthesis of generalised influenza
6925	Rondy, 2015 [53]	Included in meta-analysis of H3N2, H1N1, B
1400	Rondy, 2017a [54]	Included in meta-analysis of H3N2 elderly
676	Rondy, 2017b [55]	Narrative synthesis of 2 prior seasons
2079	Rose, 2020[56]	Narrative synthesis of 2 prior seasons
466	Saito, 2017 [57]	Narrative synthesis of generalised influenza
863	Saito, 2018 [58]	Narrative synthesis of generalised influenza and three prior seasons
1073	Shinjoh, 2018[59]	Narrative synthesis of generalised influenza
6924	Simpson, 2015[60]	Narrative synthesis of generalised influenza
6921	Skowronski, 2012[61]	Included in meta-analysis of H1N1
6922	Skowronski, 2014a [62]	Included in meta-analysis of B, B/Yam, B/Vic.
6920	Skowronski, 2014b [63]	Included in meta-analysis of H3N2, H1N1, B, B/Vic.

Covidence No.	Author, year	Status
6923	Skowronski, 2015 [64]	Included in meta-analysis of H1N1, B, B/Yam.
120	Skowronski, 2016 [65]	Included in meta-analysis of B and H3N2, B, B/Yam adults. Narrative synthesis of two prior seasons.
505	Skowronski, 2017a [66]	Included in meta-analysis of H3N2. Narrative synthesis of two prior seasons
781	Skowronski, 2017b [67]	Included in meta-analysis of H1N1, B, B/Vic. Narrative synthesis of two prior seasons
1236	Skowronski, 2018 [68]	Included in meta-analysis of B/Yam
1836	Skowronski, 2019 [69]	Included in meta-analysis of H3N2
3372	Skowronski, 2022[70]	Included in meta-analysis of H3N2
31	Smithgall, 2016 [71]	Narrative synthesis of generalised influenza
2060	Song, 2020 [72]	Included in meta-analysis of B elderly
6919	Sullivan, 2013 [73]	Narrative synthesis of generalised influenza
1409	Sullivan, 2017 [74]	Included in meta-analysis of H3N2, B
6918	Syrjänen, 2014 [75]	Narrative synthesis as unable to convert estimates to odds ratios
6917	Thompson, 2014 [76]	Narrative synthesis of pooled seasons
58	Thompson, 2016 [77]	Included in meta-analysis of B children. Narrative synthesis of pooled seasons
98	Valenciano, 2016 [78]	Eligible for meta-analysis but not included due reanalysed data. Narrative synthesis.
1190	Valenciano, 2018 [79]	Included in meta-analysis of H3N2, H1N1, B
452	Zhang, 2017 [80]	Narrative synthesis of generalised influenza
1056	Zhang, 2018 [81]	Included in meta-analysis of H3N2, H1N1
2569	Zhang, 2020 [82]	Included in meta-analysis of H1N1, H3N2 children
232	Zimmerman, 2016 [83]	Included in meta-analysis of B/Yam

5.1 A(H1N1)pdm09

2010-2011

Gherasim 2017, Jiménez-Jorge 2012, Martinez-Baz 2013, Martinez-Baz 2017

- Keep Gherasim 2017 based on preference of age range $\geq$9 years to reduce heterogeneity in age, drop all others due to same cohort

2012-2013

Rondy 2015, Valenciano 2018, Martinez-Baz 2017

- Rondy 2015 uses different study setting to Martinez-Baz and Valenciano papers
- Drop Martinez-Baz 2017, Valenciano includes the Martinez-Baz study cohort with additional study site

2013-2014

Valenciano 2018, Gherasim 2017, Martinez-Baz 2017

- Keep Valenciano, drop Gherasim 2017 and Martinez-Baz 2017, Valenciano includes the same study cohort with additional study sites

Kim 2021, Gaglani 2016

- Keep Kim 2021 as most recent publish estimate is used due to reanalysed data across both papers

2014-2015

Valenciano 2016, Valenciano 2018

- Keep Valenciano 2018 based on preference of age range $\geq$9 years to reduce heterogeneity in age, same study population otherwise

2015-2016

Kissling 2018, Valenciano 2018, Gherasim 2017, Martinez-Baz 2017

- Drop Gherasim 2017 and Martinez-Baz 2017, Valenciano and Kissling include the same study cohort with additional study sites
- Drop Kissling 2018, as most recent publish estimate is used due to reanalysed data across both papers

Kim 2021, Jackson 2017

- Keep Kim 2021 as most recent publish estimate is used due to reanalysed data across both papers

5.2 A(H3N2)

2011-2012

Nichols 2019, Skowronski 2014b

- Keep both, different study populations

Valenciano 2018, Gherasim 2017

- Drop Gherasim 2017, Valenciano includes the Gherasim study cohort with additional study sites

2012-2013

Nichols 2019, Skowronski 2017a, Skowronski 2014a

- Nichols 2019 uses different study setting to Skowronski papers
- Drop Skowronski 2014a, based on preference of age range ≥9 years to reduce heterogeneity in age

Kim 2021, McLean 2015, McLean 2014

- Keep Kim 2021 only, most recent publish estimate is used due to reanalysed data across all three papers

2013-2014

Valenciano 2018, Gherasim 2017

- Drop Gherasim 2017, Valenciano includes the Gherasim study cohort with additional study sites

2014-2015

Nichols 2019, Skowronski 2016, Skowronski 2017a

- Nichols 2019 uses different study setting to Skowronski papers

2010-2011

Gherasim 2017, Jiménez-Jorge 2012, Martinez-Baz 2013, Martinez-Baz 2017

- Keep Gherasim 2017 based on preference of age range ≥9 years to reduce heterogeneity in age, drop all others due to same cohort

2012-2013

Rondy 2015, Valenciano 2018, Martinez-Baz 2017

- Rondy 2015 uses different study setting to Martinez-Baz and Valenciano papers
- Drop Martinez-Baz 2017, Valenciano includes the Martinez-Baz study cohort with additional study sites

2013-2014

Valenciano 2018, Gherasim 2017, Martinez-Baz 2017

- Keep Valenciano, drop Gherasim 2017 and Martinez-Baz 2017, Valenciano includes the same study cohort with additional study sites

Kim 2021, Gaglani 2016

- Keep Kim 2021 as most recent publish estimate is used due to reanalysed data across both papers

2014-2015 Valenciano 2016, Valenciano 2018

- Keep Valenciano 2018 based on preference of age range ≥9 years to reduce heterogeneity in age, same study population otherwise

2015-2016

Kissling 2018, Valenciano 2018, Gherasim 2017, Martinez-Baz 2017

- Drop Gherasim 2017 and Martinez-Baz 2017, Valenciano and Kissling include the same study cohort with additional study sites
- Drop Kissling 2018, as most recent publish estimate is used due to reanalysed data across both papers

Kim 2021, Jackson 2017

- Keep Kim 2021 as most recent publish estimate is used due to reanalysed data across both papers

Skowronski 2016, Skowronski 2018

- Drop Skowronski 2016, based on preference of age range ≥9 years to reduce heterogeneity in age

Valenciano 2016, Valenciano 2018, Gherasim 2017

- Drop Gherasim 2017, Valenciano includes the Gherasim study cohort with additional study sites
- Drop Valenciano 2016, based on preference of age range ≥9 years to reduce heterogeneity in age

Kim 2021, Petrie 2016, Petrie 2017, Zimmerman 2016, McLean 2017, McLean 2018

- Keep Petrie 2016 and Petrie 2017 as each use different study setting to all other papers, keep Kim 2021 as most recent publish estimate is used due to reanalysed data across all three papers
- Drop Zimmerman and McLean studies as same study cohort (US Flu VE Network) or shared study sites and likely cohort cross over with Kim 2021, plus preference of age range ≥9 years to reduce heterogeneity in age

2016-2017

Kissling 2019b, Rondy 2017, Valenciano 2018

- Rondy 2017 uses different study setting to Kissling and Valenciano papers
- Keep Kissling 2019b as most recent publish estimate is used due to reanalysed data across both papers

Flannery 2019, Kim 2021

- Keep Kim 2021 as most recent publish estimate is used due to reanalysed data across both papers

Upon update of our search on 13 June 2022, H3N2 estimates based on reanalysed data were identified in Skowronski 2022 spanning seasons 2016-2017 and 2017-2018. We chose to retain our originally included estimates in this case.

5.3 Influenza B

2012-2013
Rondy 2015, Valenciano 2018, Gherasim 2017

- Rondy 2015 uses different study setting to Gherasim and Valenciano papers; drop Gherasim 2017, Valenciano includes the Gherasim study cohort with additional study sites

2014-2015
Nichols 2019, Skowronski 2016

- Keep both as each use different study setting

Valenciano 2016, Valenciano 2018, Gherasim 2017

- Drop Gherasim 2017, Valenciano includes the Gherasim study cohort with additional study sites; drop Valenciano 2016, keep Valenciano 2018 based on preference of age range ≥9 years to reduce heterogeneity in age

2015-2016
Kissling 2018, Valenciano 2018, Gherasim 2017

- Drop Gherasim 2017, Valenciano and Kissling include the same study cohort with additional study sites; drop Kissling 2018, keep Valenciano 2018 based on preference of age range ≥9 years to reduce heterogeneity in age

2016-2017
Flannery 2019, Kim 2021

- Keep Kim 2021 as most recent publish estimate is used due to reanalysed data across both papers

5.4 B/Yamagata

2011-2012
Skowronski 2014b, Skowronski 2018

- Drop Skowronski 2014b, keep Skowronski 2018 based on preference of age range ≥9 years to reduce heterogeneity in age

2014-2015
Skowronski 2016, Skowronski 2018

- Drop Skowronski 2016, keep Skowronski 2018 based on preference of age range ≥9 years to reduce heterogeneity in age

5.5 Multiple prior seasons

2014-2015 Skowronski 2017a, Skowronski 2016

- Drop Skowronski 2017a, keep Skowronski 2016 based on preference of broader age group

6 Summary of study designs

Supplementary Table 2: Methods of vaccine status ascertainment, recruitment, eligibility and inclusion criteria, and adjustment variables of articles included in the meta-analysis and/or qualitative synthesis.

**Author, publication year**	**Vaccine status ascertainment**	**Participant recruitment method**	**Eligibility, inclusion criteria and case definition**	**Adjustment variables**
Boddington, 2019 [1]	Official record	Identified from the Respiratory DataMart System (national sentinel laboratory surveillance system).	Specimen date in 2015–2016 influenza season between week 40 of 2015 and week 20 of 2016, aged 2-16 years old (on 31st August 2015), resident in England.	Age group, sex, IMD, ethnicity, region, month, and risk group
Buchan, 2017 [2]	Official record	Canadian Institute of Health Information Discharge Abstract Database	Specimen collected $\geq$3 days of admission, 1 hospitalisation with specimen per individual per season (first hospitalization if multiple)	Age (in months), season, and time within season (month relative to peak)
Buchan, 2018 [3]	Official record	Identified hospitalisations using Discharge Abstract Database, emergency department visits using Ambulatory Care Database, office visits using physician billing claims data (Supplemental Enhanced Service Event system).	Children aged 2-17 years who received medical attention and were tested for influenza during the 2012-2013 to 2015-2016 influenza seasons in Alberta in hospitals, emergency departments, and physician offices.	Age, influenza season, presence of any co- morbidity, and calendar month within influenza season (relative to the peak month of influenza activity)
Casado, 2016 [4]	Official record	Patients hospitalized with ILI or acute respiratory diseases were routinely swabbed for influenza testing.	Patients aged $\geq$65 years admitted to any participating hospitals for >24 hours with influenza infection, residence in any of the participating regions, provision of signed informed consent.	Sex, age (65–79 and $\geq$80 years), Barthel index, corticoid treatment, pneumonia in the previous 2 years, smoking, major chronic conditions, (0, 1, >1), antiviral treatment, and region
Casado, 2018 [5]	Official record	Patients admitted with ILI or acute respiratory disease in participant hospitals were routinely swabbed regardless of disease severity or vaccination status.	Aged 65 years or older, admitted to hospital for more than 24 hours with laboratory-confirmed influenza.	Sex, age, hospital site, influenza season, number of chronic conditions, Barthel Index score, number of visits to primary care and hospital in the previous year, pneumococcal vaccination, diagnosis of pneumonia in the previous 2 years, and treatment with corticosteroids administered orally in the previous month

Author, publication year	Vaccine status ascertainment	Participant recruitment method	Eligibility, inclusion criteria and case definition	Adjustment variables
Castilla, 2011 [6]	Official record	Electronic records of physicians and laboratories and a nested case–control analysis of swabbed patients in the region of Navarre, Spain. Influenza surveillance based on automatic reporting of cases from all primary healthcare centres.	Non-institutionalised persons covered by the Regional Health Service (95% of the population of the region) with known pre-existing major chronic conditions (heart disease, lung disease, renal disease, cancer, diabetes, cirrhosis, dementia, stroke, immunodeficiency, and body mass index of 40 or greater). Cases of MA-ILI defined according to the International Classification of Primary Care version 2 (code R80). All hospitalised patients with ILI or other acute respiratory diseases were swabbed for influenza virus testing. In addition, through a sentinel network composed of a representative sample of primary healthcare physicians covering 16% of the population, nasopharyngeal and pharyngeal swabs were taken from all patients with MA-ILI, after obtaining verbal informed consent.	Sex, age (1-14; 15-59; ≥60 years), children in the household, urban/rural residence, healthcare setting (primary healthcare, emergency room, hospitalisation) and date (Week 43–49 2010; Week 50 2010–Week 1 2011; Week 2–3 2011)
Castilla, 2016 [7]	Official record	Influenza surveillance based on automatic reporting of cases of MA-ILI from all primary healthcare centres and hospitals. A sentinel network composed of a representative sample of primary healthcare physicians, covering 18% of the population collected swabs from all ILI patients. The protocol for influenza cases in hospitals establishes early detection and nasopharyngeal and pharyngeal swabbing of all hospitalized patients with ILI.	Patients in primary health care or hospitals with ILI (considered to be the sudden onset of any general symptom (fever or feverishness, malaise, headache, or myalgia) in addition to any respiratory symptom (cough, sore throat or shortness of breath)). Symptoms begun <5 days.	Sex, age group (<5, 5–14, 15–44, 45–64, 65–84, and ≥85 years), major chronic conditions, three-week periods, and healthcare setting (primary healthcare and hospital)

Author, publication year	Vaccine status ascertainment	Participant recruitment method	Eligibility, inclusion criteria and case definition	Adjustment variables
Castilla, 2017 [8]	Official record	Influenza Surveillance System in Spain, a sentinel network of primary healthcare physicians. Influenza surveillance was based on automatic reporting of cases of ILI from all primary healthcare physicians and hospitals.	Patients diagnosed with ILI, whose symptoms had begun <5 days previously. In hospitals, the protocol specified early detection and swabbing of all hospitalised patients with ILI. Persons covered by the Navarre Health Service since 2012, swabbed between 1 December 2016 (beginning of continued detection of influenza virus) and 31 January 2017.	Age groups (9–24, 25–44, 45–64, 65–84 and ≥85 years), sex, major chronic conditions (body mass index ≥40 kg/m2, cancer, liver cirrhosis, dementia, diabetes mellitus, immunodeficiency, heart disease, renal disease, respiratory disease, rheumatic disease and stroke), healthcare setting (primary healthcare and hospital), and month of swabbing
Castilla, 2018 [9]	Official record	Influenza epidemiological and virological surveillance in primary healthcare and hospitals. Influenza surveillance relied on all primary healthcare physicians and hospitals automatically reporting ILI cases. In hospitals, early detection and swabbing of all hospitalised patients with ILI was specified by the protocol.	Study population included individuals covered by the Navarre Health Service since 2012. All ILI patients who were swabbed in December 2017 and January 2018 were considered. Symptoms had appeared less than five days before.	Age groups (9–24, 25–44, 45–64, 65–84 and ≥85 years), sex, major chronic conditions, healthcare setting (primary healthcare and hospital), and month of swabbing
Castilla, 2020 [10]	Official record	Influenza surveillance in primary healthcare and hospitals. A sentinel network of primary healthcare physicians, covering 16% of the Navarre population, collected nasopharyngeal and pharyngeal swabs from their outpatients diagnosed with ILI.	ILI defined by sudden onset of any general symptom (fever or feverishness, malaise, headache or myalgia) in addition to any respiratory symptom (cough, sore throat or shortness of breath). Symptoms appeared <5 days before. Protocol for hospitals in the region specified early detection and swabbing of all hospitalised patients with ILI.	Age groups (9–44, 45–64 and ≥65 years), major chronic conditions, healthcare setting (primary healthcare and hospital), and month of swabbing
Cheng, 2017 [11]	Self-report and official record	FluCAN hospital sentinel surveillance program.	Presentation at participating sentinel sites, admitted with ARI, >9 years with test performed.	Age, sex, pregnancy, Indigenous ethnicity, any comorbidities; homelessness, residence in long-term-care facility, and current smoking, year, site
Dominguez, 2017 [12]		Admissions to participating hospitals between December 2013 and March 2015.	Aged ≥65 years hospitalised for at least 24 hours.	Propensity score (PS) analysis was used. The PS was created using a logistic regression model with influenza vaccination status as the outcome and demographic variables, medical conditions, and functional status as independent variables

Author, publication year	Vaccine status ascertainment	Participant recruitment method	Eligibility, inclusion criteria and case definition	Adjustment variables
El Omeiri, 2018 [13]	Official record	REVELAC-i multicentre VE evaluation in nine Latin American countries. Surveillance staff at sentinel hospitals identified SARI patients. Hospitals aimed to collect specimens from all SARI patients in Argentina, Brazil, Chile, Costa-Rica, Honduras and Paraguay and from a convenience sample of five weekly SARI patients in Colombia, El Salvador and Panama. The study start date for each country was (1) after the start of the country's 2013 national influenza vaccination campaign, (2) after confirmation of the start of local influenza circulation by study leads, and (3) after the identification of the first SARI patient with RT-PCR confirmed influenza. The study period ended on the last day of local influenza circulation in 2013 as determined by study leads.	SARI patients defined as persons presenting with fever (i.e., measured temperature $\geq$38°C or parental- or self-reported history of fever), cough, and difficulty breathing who were hospitalised.	Month of illness onset, presence of at least one pre-existing condition, and age (years)
Ferdinands, 2019 [14]	Self-report and official record	US Hospitalized Adult Influenza Vaccine Effectiveness Network (HAIVEN). Study staff reviewed daily admissions to identify eligible patients using a broad range of qualifying symptoms or syndromes consistent with ARI. Recruitment began when there was laboratory evidence of increasing local influenza activity.	Patients $\geq$18 years of age, respiratory specimen collected $\leq$10 days from illness onset, $\leq$72 hours from hospital admission. Patients were eligible if they had a respiratory condition accompanied by evidence of acute infection based on review of chief complaints, admitting diagnoses, and summary of the initial clinical evaluation e.g. influenza, ILI, pneumonia (with or without radiographic evidence), upper respiratory infection, cough, bronchitis, shortness of breath, nasal congestion, chest congestion, sore throat, exacerbations of cystic fibrosis, congestive heart failure, asthma or chronic obstructive pulmonary disease accompanied by at least 1 systemic sign or symptom of infection, and altered mental status accompanied by new onset of a respiratory symptom.	Age, site, sex, race, days between onset and respiratory specimen collection, date of illness onset, history of immunosuppressive conditions, number of prior-year respiratory hospitalizations, and history of respiratory disorders other than COPD

**Author, publication year**	**Vaccine status ascertainment**	**Participant recruitment method**	**Eligibility, inclusion criteria and case definition**	**Adjustment variables**
Flannery, 2019 [15]	Self-report and official record	Participating healthcare facilities in US Flu VE Network.	Aged ≥6 months seeking outpatient care for acute respiratory illness with a cough of 7 or fewer days' duration at the time of the medical visit.	Study site, patient age in months, presence of any high-risk health condition and calendar time (two-week intervals)
Fu, 2015 [16]	Official record	Sentinel surveillance hospitals in Guangzhou and administrative databases of local ministry of health.	No additional information	No additional adjustments
Gaglani, 2016 [17]	Self-report and official record	US Flu VE Network sites in Michigan, Pennsylvania, Texas, Washington, and Wisconsin.	MAARI, including cough, and onset of illness ≤7 days before enrolment; eligible subjects were born before 1 March 2013 and based on age, were eligible for vaccination by 1 September 2013.	Site, age, calendar time, any high-risk condition, sex, race/ethnicity, general health status, and interval from illness onset to enrolment
Gherasim, 2017 [18]	Self-report	cycEVA study conducted within the framework of the Spanish Influenza Sentinel Surveillance System. Systematic swabbing of patients below 65 years old (the first two patients with ILI who had consulted a sentinel physician each week) and all patients above 64 years old.	Sentinel practitioners reported cases of ILI on a weekly basis according to a definition that is based on the European Commission ILI case definition. ILI patients swabbed <8 days since the onset of symptoms.	Age-groups (9–14; 15–44; 45–64; >64), sex, sentinel network and week of swabbing and season

Author, publication year	Vaccine status ascertainment	Participant recruitment method	Eligibility, inclusion criteria and case definition	Adjustment variables
Grijalva, 2021 [19]	Official record	Hospitalised adults from 10 hospitals in 9 US states with prioritised enrolment of ICU admissions.	Hospitalized, age ≥18 years old, Clinically-obtained influenza test completed within 72 hours of hospital presentation; and if they met the following criteria for SARI - ≥1 sign of acute infection (feverishness, chills, measured temperature ≥38.0°C or ≤35°C, white blood cell count ≥11 or ≤4 thousand cells/μl, c-reactive protein ≥25 mg/L, procalcitonin ≥0.25 ng/ml, or altered mental status); ≥1 sign of acute respiratory illness (cough, upper respiratory congestion, sore throat, shortness of breath, chest pain, new invasive or non-invasive mechanical ventilation, supplemental oxygen ≥2 litres/minute over baseline, or pulmonary infiltrate on chest imaging). Study patients with SARI who tested positive for influenza by either a clinically obtained RT-PCR or central laboratory RT-PCR test were classified as cases.	Study site, age, sex, race/ethnicity, calendar time (categorized as tertiles generated based on site-specific influenza activity using disease-onset dates of influenza cases), insurance status, enrolment location (ICU vs non-ICU), days from illness onset to specimen collection for influenza testing, chronic medical conditions (including cardiovascular and pulmonary diseases; kidney and gastrointestinal diseases; neurological, psychiatric, and gastrointestinal diseases; malignancies; and haematological, autoimmune, and other immunosuppressive conditions), and frailty (assessed using a questionnaire derived from Fried and colleagues)
Jackson, 2017 [20]	Self-report and official record	Influenza Vaccine Effectiveness Network (study sites in Michigan, Pennsylvania, Texas, Washington, and Wisconsin).	Patients 6 months of age or older who presented to ambulatory care clinics for ARI with a cough of ≤7 days in duration at the time of the medical visit.	Network site, age (with the use of linear tail- restricted cubic splines), presence of high-risk medical conditions, and calendar time (in 2-week intervals)
Jiménez-Jorge, 2012 [21]	Not specified	cycEVA study conducted within the framework of the Spanish Influenza Sentinel Surveillance System. Sentinel practitioners systematically swabbed the first two patients consulting for ILI in the week in less than 65 years old and all patients aged 65 years old and over.	The European Commission case definition was recommended for ILI case swabbing as follows: sudden onset of symptoms, and at least one out of these four systemic symptoms (fever or feverishness, malaise, headache, myalgia), and at least one out of these three respiratory symptoms (cough, sore throat, shortness of breath), in the absence of other suspected clinical diagnosis.	Age, week of swabbing
Kim, 2021 [22]	Self-report and official record	US Flu VE Network sites in Michigan, Pennsylvania, Texas, Washington, and Wisconsin.	Ambulatory patients aged ≥6 months presenting within 7 days of onset of acute respiratory illness with cough.	Study site, patient age, presence of ≥1 high-risk medical condition, calendar time, and season

Author, publication year	Vaccine status ascertainment	Participant recruitment method	Eligibility, inclusion criteria and case definition	Adjustment variables
Kissling, 2018 [23]	Self-report	I-MOVE+ protocol. Participating practitioners collected nasopharyngeal or combined naso- and oropharyngeal specimens from a systematic sample of consenting patients seeking medical attention for ILI.	In Hungary, only patients aged 18 years and older and in Croatia only patients aged 65 years and older were eligible. Included were patients meeting the European Union ILI case definition, swabbed ≤7 days of symptom onset, and who had not received antivirals in the 14 days prior to swabbing.	Age (restricted cubic spline or age group), onset date (restricted cubic spline), sex, chronic condition, and study site
Kissling, 2019a [24]	Self-report and official record	Influenza Monitoring Vaccine Effectiveness in Europe (I-MOVE) primary care multi- centre case control study.	GP or paediatrician presentation with ILI or ARI. Patients meeting the European Union ILI case definition were swabbed ≤7 days of symptom onset.	Age, sex, symptom onset time, presence of chronic condition, study site
Kissling, 2019b [25]	Self-report	I-MOVE/I-MOVE+ (Influenza Monitoring Vaccine Effectiveness in Europe) primary care multicentre case control study (MCCS).	Patients meeting the European Union ILI case definition, swabbed ≤7 days of symptom onset, and who had not received antivirals in the 14 days prior to swabbing.	Symptom onset date, age, sex, and presence of at least one chronic disease or other risk conditions such as pregnancy and obesity (where available)
Kwong, 2020 [26]	Official record	Canadian Institute for Health Information's Discharge Abstract Database (CIHI-DAD), the National Ambulatory Care Reporting System (NACRS) database, and the Ontario Health Insurance Plan (OHIP) database. Specimens were submitted at the discretion of clinicians as part of routine clinical care.	Community-dwelling adults aged >65 years in Ontario tested for influenza during inpatient or outpatient healthcare encounters 1 September 2010 - 31 August 2016. For participants tested multiple times in the same season, we included their earliest testing episode positive for influenza (or their earliest testing episode if all specimens tested negative for influenza). Individuals tested in multiple seasons contributed one testing episode per season, which were treated as separate units in the analysis. Patients had to be eligible for health insurance in Ontario during the previous seasons investigated.	Age, sex, census area-level neighbourhood income quintile, number of hospitalisations in the past 3 years, number of outpatient visits in the past year, receipt of home care services in the past year, number of prescription medications in the past year, comorbidities that increase the risk of influenza complications (anaemia, cancer, cardiovascular disease, dementia, diabetes, frailty, immunodeficiency due to underlying disease and/or therapy, as well as renal disease and respiratory disease), calendar time, and influenza season

Author, publication year	Vaccine status ascertainment	Participant recruitment method	Eligibility, inclusion criteria and case definition	Adjustment variables
Ma, 2017 [27]	Official record	Patients with MA-ILI were monitored by influenza virological surveillance in Beijing. Doctors at the ambulatory care clinics of sentinel hospitals screened and enrolled ILI patients. Convenience sampling was used aiming to enrol a weekly sample size of 20 (national sentinel hospital) and 15 (municipal sentinel hospital) patients.	ILI (defined as temperature $\geq$38°C with either cough or sore throat), samples collected 3 days of symptom onset, informed consent, aged $\geq$6 months, complete surveillance documentation. In Beijing, ILI patients traditionally seek medical attention at hospitals rather than at private clinics, so patients with ILI include both mild and severe cases.	Sex, age group, chronic diseases, and calendar week
Martinez-Baz, 2013 [28]	Official record	General practitioner sentinel network for influenza surveillance in Navarre.	All cases of ILI from primary healthcare centres and hospitals. ILI defined by sudden onset of any general symptom (fever or feverishness, malaise, headache or myalgia) in addition to any respiratory symptom (cough, sore throat or shortness of breath). Preferably swabbed $\leq$5 days of symptom onset. $>$6 months old.	Sex, age, major chronic conditions, outpatient visits in the previous year, swabbing within 4 days of symptom onset, health care setting, period
Martinez-Baz, 2017 [29]	Official record	Sentinel network of primary health care physicians.	Present to sentinel site and diagnosed with ILI. Symptoms beginning $<$5 days. Resident in Navarra region since 2009 and covered by Navarra health service.	Age groups ($<$5, 5–24, 25–44, 45–64, 65–84, and $\geq$85 years), sex, major chronic conditions, functional dependence, hospitalization in the previous 12 months, healthcare setting (primary healthcare and hospital), and season and month of sample collection
Martinez-Baz, 2021 [30]	Official record	Influenza surveillance was based on automatic reporting of cases of MA-ILI from all primary healthcare centres and hospitals. Sentinel network composed of a representative sample of primary healthcare physicians.	MA-ILI defined by sudden onset of any general symptom (fever, malaise, headache or myalgia) in addition to any respiratory symptom (cough, sore throat or dyspnoea). Swabbed after verbal informed consent. Symptoms had begun $<$5 days before the patient consultation. Continued residence in the region during the previous 5 years.	Age groups (9-44, 45-64, 65-84 and $\geq$85 years), major chronic conditions, and month-season of sample collection

Author, publication year	Vaccine status ascertainment	Participant recruitment method	Eligibility, inclusion criteria and case definition	Adjustment variables
Martinez-Baz, 2021b [31]	Official record	Influenza surveillance was based on automatic reporting of cases of medically attended influenza-like illness (ILI) from all primary healthcare centres and hospitals in Navarre.	ILI defined as the sudden onset of any general symptom (fever or feverishness, malaise, headache, or myalgia) in addition to any respiratory symptom (cough, sore throat, or shortness of breath). Swabbed after verbal informed consent, from all patients diagnosed with ILI and whose symptoms had begun within the previous 5 days. Included only patients with continuous residence in the region during the 5 years before the analysed influenza season. Children younger than 9 years, healthcare workers, and nursing homes residents were excluded. Cases were diabetic patients who were hospitalized for ILI and confirmed for influenza virus by RT-PCR.	Age group (9–64, 65–84, and ≥85 years), other major chronic conditions, and month–season of sample collection
McLean, 2014 [32]	Official record	Active recruitment during clinical encounter for ARI.	Community-dwelling residents of a 14-zip-code area around Marshfield, Wisconsin, with ≥12 months of continuous residency. From 2004–2005 through 2006–2007, cohort was restricted to individuals for whom vaccination was recommended based on age or the presence of a high-risk medical condition. In 2007–2008 and all subsequent seasons, the cohort included all individuals aged ≥6 months living in the community. Analysis included either the first enrolment (if all were negative) or the first enrolment associated with a positive influenza test.	Age, gender, high-risk conditions, interval (days) from onset to sample collection, and influenza diagnosis code in prior seasons, or in multiple prior years Age, sex, high-risk conditions, season, interval (days) from onset to sample collection, and influenza diagnosis code in prior seasons
McLean, 2015 [33]	Official record	US Flu VE Network sites in Marshfield, Wisconsin; south-eastern Michigan (Ann Arbor and Detroit); Temple-Belton, Texas; Seattle, Washington; and Pittsburgh, Pennsylvania.	Aged ≥6 months seeking outpatient medical care for an ARI with cough, duration of illness was ≤7 days, no receipt of antiviral medication prior to enrolment.	Network site, subject age, presence of high-risk health conditions, and calendar time (2-week intervals)

Author, publication year	Vaccine status ascertainment	Participant recruitment method	Eligibility, inclusion criteria and case definition	Adjustment variables
McLean, 2017 [34]	Official record	Patients seeking outpatient medical care for febrile ARI.	Community-dwelling children aged 2–17 years seeking outpatient medical care for febrile ARI. Children were eligible if they presented with an ARI with fever (oral temperature $\geq$ 100.0°F at study visit, history of fever reported by parent, or use of antipyretic medication before study visit), with symptom duration of $<$5 days, and without receipt of antiviral medication before enrolment.	Age, calendar time (modelled as a series of dichotomous variables representing 4-week intervals), site, high risk health status, number of outpatient visits in the past year
McLean, 2018 [35]	Official record	Patients seeking outpatient medical care for ARI.	Community-dwelling children aged 2 to 17 years who sought outpatient medical care for ARI with fever (oral temperature $\geq$100.0°F at study visit, history of fever reported by parent, or use of antipyretic medication before study visit), with symptom duration $<$5 days, without receipt of antiviral medication before enrolment, eligible when influenza circulated locally.	LAIV estimates adjusted for age, site, and peak influenza period. LAIV models for influenza A(H1N1)pdm09 and influenza B also included season and number of outpatient visits in the past year. IIV estimates adjusted for age, site, peak influenza period, and number of outpatient visits in the past year. IIV models for influenza A(H1N1)pdm09 and influenza B also included season
Mira-Iglesias, 2018 [36]	Self-report and official record	Valencia Hospital Surveillance Network for the Study of Influenza and Respiratory Viruses Disease (VAHNSI). Study staff screened consecutive hospital admissions through the emergency department.	Written informed consent, resident in the hospital catchment area, non-institutionalised, no previous hospital discharge in the last 30 days, and reported symptoms of ILI, defined as reported fever or feverishness, malaise, myalgia or headache and shortness of breath, sore throat or cough, within 7 days of admission.	Age, sex, number of underlying chronic conditions, previous hospital admissions in the last 12 months, general practitioner consultations in the last 3 months, smoking habits, socioeconomic class, days from onset of symptoms to swabbing, and hospital as fixed effect, and epidemiological week at admission included as a random effect

Author, publication year	Vaccine status ascertainment	Participant recruitment method	Eligibility, inclusion criteria and case definition	Adjustment variables
Mira-Iglesias, 2019 [37]	Official record	Valencia Hospital Network for the Study of Influenza (VAHNSI). Prospective active-surveillance hospital-based study in the Valencia Region in Spain. Study staff screened consecutive hospitalised patients who had been discharged from the emergency department to be further admitted as inpatients.	≥60 years old, admitted in hospital through the emergency department with a diagnosis possibly related to influenza, resident in one of the participating hospitals' catchment areas. Signed written informed consent and reported symptoms of ILI (defined as per the European Union ILI-case definition, as fever or feverishness, malaise, myalgia or headache and shortness of breath, sore throat, or cough), which had occurred ≤7 days prior to admission to the emergency department, recruited during influenza season.	Age, number of chronic conditions, sex, smoking habits, and epidemiological week at admission, or in additional analyses Age, number of chronic conditions, sex, socioeconomic status (occupation), admission in the last 12months, number of GP visits in the last 3 months, smoking habits, obesity status, days between symptoms onset and swab, hospital, and epidemiological week at admission, or Age, sex, and epidemiological week at admission
Nichols, 2019 [38]	Self-report and official record	Serious Outcomes Surveillance (SOS) Network in 5-7 provinces: an active surveillance for influenza hospitalizations by reviewing all daily admissions of adult patients (≥16 years of age) to medical wards and medical and coronary intensive care units to identify patients eligible for enrolment.	Patients (≥16 years of age) to medical wards and medical and coronary intensive care units. Tested for influenza <7 days of hospital admission; patients were only eligible to become test-negative controls if they were tested within 7 days of onset of symptoms.	All; age, antiviral use prior to admission, and frailty (in patients ≥65 years of age)<br><br>Additional all ages adjustments<br><br>2011–2012; smoking, number of medications, and admission from a long-term care facility. 2012–2013; pregnancy, smoking, and number of medications. 2013–2014; pregnancy. 2014–2015; pregnancy, and smoking Additional <65 years adjustments<br><br>2011-2012, 2013-2014 and 2014-2015; pregnancy. 2012-2013; smoking, and pregnancy<br><br>Additional ≥65 years adjustments<br><br>2012-2013; sex, smoking, and number of medications. 2011-2012, 2013-2014; no additional. 2014-2015; smoking

Author, publication year	Vaccine status ascertainment	Participant recruitment method	Eligibility, inclusion criteria and case definition	Adjustment variables
Ohmit, 2014 [39]	Official record	US Flu VE Network: patients presenting to ambulatory care facilities, including urgent care clinics, affiliated with the Group Health Cooperative, Seattle, Washington; the Marshfield Clinic Research Foundation, Marshfield, Wisconsin; the University of Michigan School of Public Health partnered with the University of Michigan, Ann Arbor, and Henry Ford, Detroit, Health Systems, Michigan; the University of Pittsburgh Schools of Health Sciences partnered with the University of Pittsburgh Medical Center, Pittsburgh, Pennsylvania; and Scott & White Healthcare, Texas A&M Health Science Center College of Medicine, Temple, Texas.	Patients with ARI seeking medical care at study sites aged $\geq$6 months on 1 September 2011 and thus eligible for influenza vaccination, illness characterized by cough and or fever/feverishness of $<$7 days' duration.	Network centre, subject age in months, sex, race/ethnicity categories, presence of high-risk health conditions, self-rated health status, time (days) between illness onset and specimen collection, and calendar time
Ohmit, 2015 [40]	Official record	Households were derived from persons who had selected a primary healthcare provider from the University of Michigan Health System in Ann Arbor. Households were instructed at enrolment and via weekly email reminders to report all acute respiratory illnesses.	Eligible households (shared residence) comprised at least 4 participating members, at least 2 of whom were children aged $<$18 years. ARIs were defined as $\geq$2 of the following symptoms: cough, fever or feverishness, nasal congestion, chills, headache, body aches, and/or sore throat. Subjects with eligible illnesses had a combined throat and nasal swab specimen (or, for children aged $<$3 years, a nasal swab specimen only) collected at an illness visit within 7 days of illness onset.	Age in months (natural cubic spline) and documentation (present or absent) of high-risk health status

Author, publication year	Vaccine status ascertainment	Participant recruitment method	Eligibility, inclusion criteria and case definition	Adjustment variables
Ohmit, 2016 [41]	Official record	Households were derived from persons who had selected a primary healthcare provider from the University of Michigan Health System in Ann Arbor.	Eligible households (shared residence) were composed of at least 4 participating members, of whom at least 2 were children (aged <18 years). Households were instructed at enrolment and via weekly email reminders to report all ARIs in which ≥2 of the following symptoms were present: cough, fever or feverishness, nasal congestion, chills, headache, body aches, and/or sore throat. Swabs were collected at an illness visit ≤7 days of illness onset. Age in months (natural cubic spline) and medical record–documented high-risk health status (present/absent)	
Ortqvist, 2018 [42]	Official record	Annual closed cohorts registered in Stockholm at the start of each season. SmiNet is the national electronic surveillance system for the reporting of communicable diseases. Since December 1, 2015, it is mandatory for all Swedish laboratories to report findings of influenza to SmiNet.	≥66 years of age, living in Stockholm County.	Age, sex, socio-economic status, co-morbidity, and Pandemrix® vaccination
Pebody, 2013 [43]	Official record	Data was derived from five primary-care influenza sentinel surveillance schemes in England, Northern Ireland, Scotland, and Wales. Details of the Royal College of General Practitioners, Health Protection Agency Regional Microbiology Network, Public Health Wales and Health Protection Scotland.	Persons presenting during the study period in a participating practice with an acute ILI who were swabbed and then tested for influenza.	Age group, gender, time period and surveillance scheme
Pebody, 2017 [44]	Self-report & Official record	Registered population of five sentinel general practice surveillance networks across the UK: the Royal College of General Practitioners Research and Surveillance Centre network, the Public Health England Specialist Microbiology Network and the national sentinel schemes of Northern Ireland, Scotland, and Wales.	Patients presenting to their general practitioner during the study period with an acute ILI who the GP obtained consent from and swabbed during the consultation. A case of ILI was defined as an individual who presented with an acute respiratory illness with physician-diagnosed fever or complaint of feverishness in the previous 7 days.	Age group, sex, month, pilot area and surveillance scheme

**Author, publication year**	**Vaccine status ascertainment**	**Participant recruitment method**	**Eligibility, inclusion criteria and case definition**	**Adjustment variables**
Pebody, 2019 [45]	Self-report & Official record	Registered population of five sentinel general practice surveillance networks across the UK: the Royal College of General Practitioners Research and Surveillance Centre network, the Public Health England Specialist Microbiology Network and the national sentinel schemes of Northern Ireland, Scotland, and Wales.	Patients presenting to their general practitioner during the study period with an acute ILI who the GP obtained consent from and swabbed during the consultation. A case of ILI was defined as an individual who presented with an acute respiratory illness with physician- diagnosed fever or complaint of feverishness in the previous 7 days.	Age group, risk-group, sex, month, pilot area and surveillance scheme
Pebody, 2020a [46]	Official record	Respiratory DataMart Surveillance system (RDS).	Residents in England 2– 17 years of age (on August 31st, 2018) who were admitted to hospital and who had a respiratory swab taken between week 40 2018 and week 20 2019 which was tested for influenza with RT-PCR by one of the RDS laboratories.	Age group, month, region, Index of Multiple Deprivation (IMD), risk group
Pebody, 2020b [47]	Official record	Respiratory DataMart Surveillance system (RDS).	Residents in England $\geq$65 years of age (on August 31st, 2018) who were admitted to hospital and who had a respiratory swab taken between week 40 2018 and week 20 2019 which was tested for influenza with RT-PCR by one of the RDS laboratories.	Age-group, gender, month, region, and risk group
Pebody, 2020c [48]	Self-report & Official record	Registered populations of five sentinel general practice surveillance networks across the UK, all of which undertake respiratory swabbing of eligible patients. The five schemes are: the Royal College of General Practitioners Research and Surveillance Centre network, the Public Health England Specialist Microbiology Network and the national sentinel schemes of Northern Ireland, Scotland, and Wales.	Patients presenting to their general practitioner with an acute ILI, who the GP consented verbally and swabbed during the consultation. A case of ILI was defined as an individual who presented with an ARI with physician-diagnosed fever or complaint of feverishness in the previous seven days. The combination of acute onset, cough, and systemic symptoms (fever, headache, myalgia etc.) was recommended as a guide to diagnosis.	Age (by $<$2, 2–11, 12–17, 18–44, 45–64 and $\geq$65 years), month of onset of symptoms, surveillance scheme (two England schemes, Wales, Scotland, and Northern Ireland), risk-group, sex, and residence in an area where all primary school children were offered LAIV4

Author, publication year	Vaccine status ascertainment	Participant recruitment method	Eligibility, inclusion criteria and case definition	Adjustment variables
Petrie, 2016 [49]	Self-report & Official record	Adults (aged ≥18 years) hospitalized for treatment of ARIs at the University of Michigan Hospital in Ann Arbor and the Henry Ford Hospital in Detroit were prospectively enrolled. Each weekday, trained study staff at both hospitals reviewed health system electronic medical records to identify newly admitted (≤48 hours) patients with diagnoses of interest. Enrolment began after circulation of laboratory-confirmed influenza was identified through local surveillance.	ARIs were broadly defined based on admission diagnoses and included ILIs (influenza, respiratory infection, cough, bronchitis), pneumonias, and exacerbations of asthma or chronic obstructive pulmonary disease. Patients with other diagnoses, including respiratory distress, shortness of breath, and acute exacerbations of other chronic respiratory conditions (e.g., congestive heart failure), were also eligible if evidence of an ARI (e.g., new or worsening cough) was included in the admission note. Onset of ARI ≤ 10 days prior to enrolment.	Hospital site, natural cubic spline functions of age (in months), sex, frailty score, Charlson comorbidity index (CCI), days between illness onset and specimen collection, calendar time of illness onset (categorized as 2-week intervals)
Petrie, 2017 [50]	Self-report & Official record	Recruited based on selection of a primary health care provider from within the University of Michigan Health System, targeted by direct mail.	Eligible households with ≥3 members, including ≥2 children <18 years, were identified, recruited, and enrolled from June- September 2014. All ARI at illness onset defined by symptoms tailored to those ≥3 years (≥2 of cough, fever/feverishness, nasal congestion, chills, headache, body aches, or sore throat) and, separately, children <3 years (≥2 of cough, fever/feverishness, runny nose/congestion, difficulty breathing, fussiness/ irritability, fatigue or loss of appetite). Subjects with eligible illnesses had combined throat and nasal swab specimens (children <3 years: nasal swab only) collected by study staff ≤7 days from illness onset.	Results are presented from unadjusted models because of sparse data; estimates from models adjusted for age in months (natural cubic spline) and medical record documented high-risk health status (present/absent) were not substantially different
Powell, 2020 [51]	Official record	End of season hospital laboratory records.	<18 years old, at least 6 months of age by September 1, 2017 (i.e., born before March 1, 2017), and seen with acute (i.e., ≤7 days' duration) symptoms of predominant respiratory infection (with or without fever) during the period when influenza was circulating.	Unadjusted

Author, publication year	Vaccine status ascertainment	Participant recruitment method	Eligibility, inclusion criteria and case definition	Adjustment variables
Rao, 2021 [52]	Self-report & Official record	Children with ILI evaluated in an emergency department or urgent care setting.	Children aged 6 months - 8 years of age with ILI defined by a temperature of $\geq$37.8 °C and at least 1 of the following: cough, sore throat, runny nose, or nasal congestion.	Age, presence of a high-risk medical condition, race, insurance status, and month and year of illness onset
Rondy, 2015 [53]	Self-report & Official record	Active screening of all admissions for potentially influenza-related conditions, including acute myocardial infarction or acute coronary syndrome; heart failure; pneumonia and influenza; chronic pulmonary obstructive disease; myalgia; altered consciousness, convulsions, febrile convulsions; respiratory abnormality; shortness of breath; respiratory or chest symptoms; acute cerebrovascular disease; sepsis; and systemic inflammatory response syndrome. Invited patients with an onset of ILI symptoms (one systemic and one respiratory symptom) within the past seven days.	Community-dwelling adults (18 years of age or older), belonging to the target groups for vaccination as defined locally, admitted to one of the participating hospitals with no contraindication for influenza vaccination, swabbed within 7 days of illness onset.	All analyses adjusted for study site and month of symptom onset. Adjusted models adjusted for study site, month of symptom onset, age, and comorbidities
Rondy, 2017a [54]	Self-report & Official record	Hospital teams identified and swabbed patients aged 65 years and above, hospitalised with signs compatible with a SARI defined as at least one systemic and one respiratory sign or symptom.	Hospitalisation with SARI, $\geq$65 years, no contraindication for vaccination, onset of SARI in previous 7 days.	Study site, age, and onset date (modelled as a restricted cubic spline with 3 and 4 knots, respectively)

Author, publication year	Vaccine status ascertainment	Participant recruitment method	Eligibility, inclusion criteria and case definition	Adjustment variables
Rondy, 2017b [55]	Self-report & Official record	Two European networks of hospitals (InNHOVE 2011–14 & I-MOVE plus since 2015). In the participating services of each hospital, patients admitted for clinical conditions that could be related to influenza were screened for eligibility.	Community-dwelling individuals ≥65 years admitted as inpatients with influenza related illness, and who had no contraindication for influenza vaccination or previous laboratory confirmed influenza in the season. Study periods for each influenza season, study site and influenza (sub)type lasted from the week of the first to the week of the last laboratory confirmed case. Hospitalised patients who had in the past seven days at least one systemic (fever or feverishness, malaise, headache, myalgia) and at least one respiratory symptoms (cough, sore throat or shortness of breath).	Study site, or in multiple prior years Study site, month of onset, age, presence of chronic conditions and season
Rose, 2020 [56]		I-MOVE hospital network.	All consenting, community-dwelling elderly (≥65 years) admissions to participating hospitals and diagnosed with SARI (i.e., with at least one systemic and one respiratory sign or symptom) within the 7 days prior to swabbing.	Age/time model, sex and number of chronic diseases (none, one, two or more)
Saito, 2017 [57]	Official record	Patients who visited Kamigoto Hospital (KH) with an ILI between December 2008 and April 2012.	All outpatients with ILI who attended the hospital, rapid diagnostic test used to diagnose influenza A/B infection. ILI defined as a sudden onset of fever and at least one sign of coughing, a runny nose, sore throat, headache, myalgia, or fatigue. When a patient had multiple ILI episodes within the same season, all episodes included as a cluster. Multiple ILI episodes that occurred within 7 days treated as a sole ILI episode.	Sex, age group, presence of any comorbidity, type of health insurance, visiting period of the season, and MA-fluA status in the prior season. 2009-2010 season also adjusts for whether the presentation was before or after the vaccination campaign during the 2009–2010 season

Author, publication year	Vaccine status ascertainment	Participant recruitment method	Eligibility, inclusion criteria and case definition	Adjustment variables
Saito, 2018 [58]	Official record	Kamigoto Hospital (KH) has an ILI registry system that electronically records the relevant medical information and rapid diagnostic test (RDT) results of all patients with ILI symptoms. Influenza RDTs were routinely performed for all patients with ILI symptoms.	Episodes of ILI in schoolchildren aged 9 years (third- or fourth-grade elementary) to 18 years (third-grade high school) attending KH.	Age, sex, the presence of chronic conditions, duration of symptoms, season/year of visit, phase of the season and the history of rapid diagnostic test–confirmed MA-fluB during the past 3 influenza seasons, or Age, sex, the presence of chronic conditions, duration of symptoms, season/year of visit, phase of the season, and history of rapid diagnostic test confirmed MA-fluA during the past 3 influenza seasons
Shinjoh, 2018 [59]	Self-report & Official record	Databases of 21 hospitals, paediatric outpatient clinics.	Children aged 6 months - 15 years with a fever of 38°C or over and who had received an RIDT in outpatient clinics of 21 hospitals mainly located in the Greater Tokyo Metropolitan area 1 November 2016 (44th week) - 31 March 2017 (13th week).	Comorbidity, area (north, central, or south area of the Kanto region), month of onset, and age (0–15 y/o)
Simpson, 2015 [60]	Official record	A 5% representative sample of Scottish healthcare practices. Patient-level data extracted and linked to the Health Protection Scotland virology dataset. General practices in the Health Protection Scotland sentinel-swabbing scheme are requested to submit five swab samples per week to the West of Scotland Specialist Virology Centre. Also included results from swabbing carried out in primary and secondary care for routine diagnostic purposes in symptomatic patients outside the sentinel scheme.	Unclear	Unadjusted
Skowronski, 2012 [61]	Self-report	Sentinel sites offering influenza testing.	All patients presenting to participating sentinel sites within 7 days of onset of ILI, defined by acute onset of fever and cough and >1 of the following symptoms: sore throat, arthralgia, myalgia, or prostration were eligible.	Unclear

Author, publication year	Vaccine status ascertainment	Participant recruitment method	Eligibility, inclusion criteria and case definition	Adjustment variables
Skowronski, 2014a [62]	Self-report	Canadian Sentinel Practitioner Surveillance Network (SPSN). Community-based practitioners.	Patients presenting within 7 days of ILI onset. ILI defined by acute fever and cough illness with one or more of sore throat, arthralgia, myalgia, or prostration. Fever is not required for elderly patients aged ≥65 years.	Age (2-8, 9-19, 20-49, 50-64, ≥65 years), comorbidity, province, interval, week
Skowronski, 2014b [63]	Self-report	Canadian Sentinel Practitioner Surveillance Network (SPSN). Community-based practitioners.	Patients presenting within 7 days of ILI onset. ILI defined by acute fever and cough illness with one or more of sore throat, arthralgia, myalgia, or prostration. For 2011-2012, fever not required for patients aged ≥65 years.	Age (2–8, 9–19, 20–49, 50–64, ≥65 years), comorbidity, province, interval, week
Skowronski, 2015 [64]	Self-report	Canadian Sentinel Practitioner Surveillance Network (SPSN). Community-based practitioners.	Presented within 7 days of ILI onset, ≥2 years old in 2013-2014 with valid data for TIV in 2012-2013 and 2013-2014.	Age group, comorbidity, province, interval, and week (spline)
Skowronski, 2016 [65]	Self-report	Canadian Sentinel Practitioner Surveillance Network (SPSN). Community-based practitioners.	Patients presented to a sentinel site within 7 days of ILI onset. ILI defined by ARI with fever and cough and at least 1 of: sore throat, arthralgia, myalgia, or prostration. Fever not required for patients aged ≥65 years. Age ≥1 year at specimen collection.	Age group (<9, 9–19, 20–49, 50–64, ≥65 years), sex, comorbidity, province, collection interval, and calendar time (spline), or in multiple prior years
			Age ≥2 for repeat vaccination effect.	Age group (20-49, 50-64 years), sex, comorbidity, province, collection interval and calendar time (spline)
			Age ≥3 for 3 prior season repeat vaccination effect. Those with complete data for 2012–2013, 2013–2014, and 2014–2015 influenza vaccine receipt.	Age group (<9, 9–19, 20–49, 50–64, ≥65 years), sex, comorbidity, province, collection interval, and calendar time (spline)
Skowronski, 2017a [66]	Self-report	Canadian Sentinel Practitioner Surveillance Network (SPSN).	Patients presenting within 7 days of ILI onset to outpatient sentinel clinics in participating provinces (Alberta, British Columbia, Ontario, Quebec) were eligible. ILI defined by ARI requiring fever and cough and at least 1 of sore throat, arthralgia, myalgia, or prostration. Fever was not a requirement in patients aged ≥65 years.	Age group, sex, comorbidity, province, collection interval, and week of specimen collection (cubic B-spline functions with 3 equal knots)

Author, publication year	Vaccine status ascertainment	Participant recruitment method	Eligibility, inclusion criteria and case definition	Adjustment variables
Skowronski, 2017b [67]	Self-report	Canadian Sentinel Practitioner Surveillance Network (SPSN).	Presentation to sentinel sites within 7 days of ILI onset, defined as fever, cough, and ≥1 of the following: sore throat, myalgia, arthralgia, or prostration. Fever was not required for older adults ≥65 years old.	Age group, sex, comorbidity, province, collection interval, and calendar time (week of specimen collection was modelled using cubic B spline functions with 3 equally spaced knots)
Skowronski, 2019a [68]	Self-report & Official record	Canadian Sentinel Practitioner Surveillance Network (SPSN).	Patients ≥1 year old presenting within 7 days of ILI onset to sentinel-practitioners in the provinces of Alberta, British Columbia, Ontario, and Quebec. Analyses were restricted to specimens collected in January–April.	Age group, sex, comorbidity, province, specimen collection interval, and week of specimen collection
Skowronski, 2019b [69]	Self-report	Canadian Sentinel Practitioner Surveillance Network (SPSN). Includes sentinel outpatient sites in the four most populous provinces of Canada: Alberta, British Columbia, Ontario, and Quebec.	Patients presenting to a sentinel site between 1 November and 30 April were eligible for inclusion in VE analysis if ≥1-year-old and attending within 7 days of onset of ILI, defined as self-reported fever and cough and at least one other symptom of sore throat, myalgia, arthralgia, or prostration; fever not required for older adults ≥65 years.	Age group (9-19, 20-49, 50-64, ≥ 65 years), province (Alberta, British Columbia, Ontario, Quebec), specimen collection interval (≤4 days; 5-7 days) and calendar time (week of specimen collection modelled using natural cubic spline function with 3 equally spaced knots)
Skowronski, 2022 [70]	Self-report	Canadian Sentinel Practitioner Surveillance Network (SPSN) in British Columbia, Alberta, Ontario, and Quebec.	≥1 year old presenting between November and April and within 7 days of onset of ILI, defined by self-reported fever and cough and at least 1 other symptom of sore throat, myalgia, arthralgia, or prostration; fever not required for ≥65 years.	Age group (9–19, 20–49, 50–64, ≥65 years), province (Alberta, British Columbia, Ontario, Quebec), specimen collection interval (≤4 days; 5–7 days), and calendar time (week of specimen collection modelled using natural cubic spline function with 3 equally spaced knots)

Author, publication year	Vaccine status ascertainment	Participant recruitment method	Eligibility, inclusion criteria and case definition	Adjustment variables
Smithgall, 2016 [71]	Official record	5-year community-based surveillance study for ARIs in a low-income northern Manhattan neighbourhood. Followed 275 households during 1 November 2013 to 1 June 2014 to estimate VE for symptomatic, laboratory-confirmed influenza; 27 households left the study before 1 June 2014, and 27 households were enrolled after 1 November 2013.	Household reporters were queried twice weekly and at monthly visits, for ARI symptoms (rhinorrhoea/congestion, pharyngitis, cough, body aches, or feverishness) among household members. Nasal swabs were obtained at home visits from participants with ≥2 ARI symptoms, within 24 hours of symptom report, whenever possible. A child was included in the study if he/she had a vaccination history in the NYP registry or the CIR. An adult was included if he/she was a current NYP patient (defined as having ≥1 visit (e.g., primary care, obstetrics/gynaecology, family planning) or hospitalization between 1 October 2013 and 31 May 2014).	Age, sex, and chronic respiratory conditions
Song, 2020 [72]	Official record	Hospital-based influenza surveillance system (Hospital-based Influenza Morbidity and Mortality, HIMM) of South Korea.	Aged ≥65 years presenting within seven days of ILI were tested for influenza using a rapid diagnostic test at 10 university hospitals. ILI defined by sudden onset of fever (≥38°C) accompanied by ≥1 respiratory symptom, including cough, sore throat or nasal stuffiness. Nasopharyngeal swab samples were transported to the central HIMM laboratory for subtyping by multiplex respiratory viral PCR.	Age, sex, underlying medical conditions, body mass index (BMI), calendar time of illness (month) and interval (days) from ILI onset
Sullivan, 2013 [73]	Self-report	General practice sentinel surveillance scheme.	Swabbed within 8 days of symptom onset.	Age group (<18, 18–49, ≥50 years; <9 subjects not included due to too few data), high-risk health status, week, and time between onset and polymerase chain reaction request
Sullivan, 2017 [74]	Self-report & Official record	ASPREN and VicSPIN Sentinel Influenza General Practice Networks.	Presentation to sentinel practitioners with ILI and swab for PCR.	Age, calendar time (cubic spline function with 4 knots)

Author, publication year	Vaccine status ascertainment	Participant recruitment method	Eligibility, inclusion criteria and case definition	Adjustment variables
Syrjänen, 2014 [75]	Self-report & Official record	Invitation letters were sent home to addresses retrieved from the Population Register Centre, distributed to pregnant women at maternity clinics, to healthcare professionals at work and announcements were published in local newspapers. All subjects who complied with follow-up in the study 2009–10 and still living in Tampere were invited to participate in the second phase of the study through letters sent to their home addresses.	Residents of Tampere city, 18–75 years of age, community-dwelling, with full legal competence and able to communicate fluently in Finnish or Swedish. ILI defined by sudden onset of measured fever (≥38°C) and at least one sign or symptom of acute respiratory infection. Pneumonia diagnosed by a physician was also regarded as an ILI. Specimens were collected within 5 days after the onset of symptoms. During the second phase of the study, the sampling window after onset was extended for logistical reasons to 7 days.	Age group (18–49, 50–75 years), gender, underlying medical condition and pregnancy
Thompson, 2014 [76]	Self-report & Official record	Pregnancy and Influenza Project.	Participants were members of Kaiser Permanente who had at least 1 prenatal visit in the Northwest region (Portland, Oregon, metropolitan area) or the Northern California region (San Francisco Bay Area). Identified potential ARIs using daily surveillance of electronic medical records for MAARI (using ICD-9-CM codes 460–466 and 480–488). During the first season, weekly Internet- or telephone-based surveillance also monitored the occurrence of non–medically attended ARI among participants at both sites. Trained study staff collected respiratory specimens at participants' homes for ARIs that included fever and cough within 8 days of illness onset.	Site, season, trimester, age, race, ethnicity (Hispanic), high risk medical condition, whether the illness was medically attended, and days between illness onset and respiratory specimen collection
Thompson, 2016 [77]	Official record	US Flu VE Network.	Patients seeking outpatient medical care for an ARI (onset ≤7 days) with cough. Enrolled during weeks with local influenza virus circulation at the 5 Network sites. Age 6 months to 8 years.	Study site, month of enrolment, age (in months), high risk status, race/ethnicity, and days from illness onset to enrolment. The model for A(H3N2) illness also included a variable for season and an interaction term for season by month

Author, publication year	Vaccine status ascertainment	Participant recruitment method	Eligibility, inclusion criteria and case definition	Adjustment variables
Valenciano, 2016 [78]	Self-report & Official record	I-MOVE Multicentre Case–Control Study. GPs interviewed and collected nasopharyngeal specimens from all (seven study sites) or a systematic sample (in Germany) of patients consulting for ILI aged 60 (Germany, Poland, and three regions in Spain) or 65 years old (Hungary, Ireland, Italy, Portugal, Romania and three regions in Spain) and older and from a systematic sample of ILI patients in the other age groups.	GP presentations more than 14 days after the start of the national vaccination campaigns and who met the European Union ILI case definition. Swabbed within seven days of symptom onset, and no receipt of antivirals before swabbing. In Hungary, only patients aged 18 years or over were eligible for inclusion in the study.	Age (restricted cubic spline or age group), onset date (restricted cubic spline), sex, chronic condition, and study site
Valenciano, 2018 [79]	Self-report & Official record	I-MOVE primary care Multicentre Case–Control Study where practitioners recruited a systematic sample of ILI patients.	Collected swabs patients consulting for ILI by EU case definition: sudden onset of symptoms and at least one of the following systemic symptoms: fever or feverishness, malaise, headache, myalgia and at least one of the following respiratory symptoms: cough, sore throat, shortness of breath. Used the population for which influenza vaccination is recommended every season. Consultations more than 14 days after the start of national or regional seasonal influenza vaccination campaign and were swabbed less than 8 days after ILI symptom onset and did not receive influenza antivirals before swabbing.	Study site, season, age (restricted cubic splines), onset date (restricted cubic splines), chronic condition, sex

Author, publication year	Vaccine status ascertainment	Participant recruitment method	Eligibility, inclusion criteria and case definition	Adjustment variables
Zhang, 2017 [80]	Official record	Influenza outbreaks in elementary, junior high and high schools reported to Beijing Centres for Disease Control and Prevention (CDC) between November 1, 2014, and December 31, 2014. Found through 2 existing syndromic surveillance systems in Beijing that monitor ILI.	ILI (measured or self-reported temperature ≥38°C with either cough or sore throat) and febrile illnesses of any aetiology (measured or self-reported temperature ≥37.5°C). ILI outbreak defined as ten or more epidemiological-linked ILIs identified in a school within 1 week. Febrile outbreak defined as ten or more febrile illnesses within a single school classroom within 2 days. Swabs collected from up to 10 symptomatic cases from each school where an ILI or febrile outbreak was reported. Priority given to students currently sick and attending school. If this number was <10, CDC attempted to collect respiratory specimens through home visits from sick children dismissed from school within the 7 days before the outbreak because of illness. Outbreaks occurred at least 14 days after the start of each school's vaccination campaign. A school outbreak began with the index case and ended when no new cases with ILI or fever were found for 7 consecutive days.	Cluster effect (school in which influenza outbreak occurred), age group, sex, areas, BMI, chronic conditions, and onset week
Zhang, 2018 [81]	Official record	In each sentinel hospital, pharyngeal swabs from 20 or more patients with ILI who visited the outpatient clinic were collected by trained nurses per week. Used convenience sampling to select subjects.	ILI patients (i.e., temperature ≥38°C and either cough or sore throat) aged ≥6 months seeking outpatient medical care were enrolled at 23 sentinel hospitals in Beijing. Onset of MA-ILI November 1, 2015 - April 30, 2016. For the study of previous vaccination were restricted to aged ≥2 years during 2015–2016 season with valid data for TIV receipt both in 2014–2015 and 2015–2016.	Age group and week of illness onset

**Author, publication year**	**Vaccine status ascertainment**	**Participant recruitment method**	**Eligibility, inclusion criteria and case definition**	**Adjustment variables**
Zhang, 2020 [82]	Official record	School outbreaks of influenza illness were found through two existing syndromic surveillance systems in Beijing that monitor ILI.	School outbreaks of ILI (measured or self-reported temperature ≥38°C with either cough or sore throat) and febrile illnesses of any aetiology (measured or self-reported temperature ≥37.5°C). ILI outbreak defined as 10 or more epidemiological-linked ILIs identified in a school within 1 week; Febrile outbreak defined as 10 or more febrile illnesses within a single school classroom within 2 days.	Age group, sex, areas, BMI, chronic conditions
Zimmerman, 2016 [83]	Self-report & Official record	US Flu VE Network in Michigan, Pennsylvania, Texas, Washington, and Wisconsin has enrolled participants seeking outpatient medical care for an ARI with cough	ARI onset ≤7 days prior, presented with cough, date of birth before 1 March 2014, no influenza antiviral medication in the previous 7 days, not previously enrolled within 14 days.	Site, age (spline), any high-risk International Classification of Diseases, 9th Edition, Clinical Modification code in the year prior to enrolment, and calendar time

ARI: acute respiratory infection; ILI: influenza-like illness; MAARI: medically attended acute respiratory infection; MA-ILI: medically attended influenza-like illness; SARI: severe acute respiratory infection; BMI: Body Mass Index; GP: general practitioner

7 Multiple prior year history

Supplementary Table 3: Comparison of ΔVE estimates obtained from studies which examined vaccination across three consecutive seasons[a]

Study	Current Season	Age Group	A(H1N1)pdm09 ΔVE_{1P}	A(H1N1)pdm09 ΔVE_{2P}	A(H3N2) ΔVE_{1P}	A(H3N2) ΔVE_{2P}	B ΔVE_{1P}	B ΔVE_{2P}	B/Victoria ΔVE_{1P}	B/Victoria ΔVE_{2P}	B/Yamagata ΔVE_{1P}	B/Yamagata ΔVE_{2P}
Skowronski 2017a [66]	2010-2011	≥9 years			-39% (-181%, 64%)	-26% (-78%, 68%)						
Skowronski 2017a [66]	2012-2013	≥9 years			-27% (-247%, 116%)	-14% (-73%, 119%)						
Skowronski 2016 [65]	2014-2015	≥3 years			8% (-48%, 65%)	-107% (-166%, -41%)	-8% (-98%, 64%)	-34% (-82%, 37%)			-11% (-120%, 75%)	-30% (-84%, 54%)
Castilla[b] 2016 [7]	2014-2015	≥6 months			25% (-133%, 251%)	82% (-20%, 302%)	-98% (-206%, -30%)	-50% (-87%, -2%)				
McLean 2018 [35]	2014-2015 LAIV	2-17 years			30% (-131%, 285%)	67% (-34%, 320%)						
McLean 2018 [35]	2014-2015 IIV	2-17 years			42% (-51%, 184%)	32% (-48%, 173%)						
Petrie 2017 [50]	2014-2015	≥9 years			22% (-122%, 199%)	16% (-89%, 191%)						
Skowronski 2017b [67]	2015-2016	≥9 years	12% (-33%, 58%)	-27% (-58%, 20%)			42% (-18%, 111%)	1% (-41%, 72%)	38% (-43%, 133%)	7% (-44%, 104%)		
Rose 2020 [56]	2017-2018	≥65 years			15% (-39%, 89%)	13% (-31%, 85%)	16% (-25%, 64%)	10% (-22%, 56%)				

[a] ΔVE_{1P} measures the difference in VE among people vaccinated in the current & one of the two prior seasons versus those vaccinated in the current season and neither of the prior two. ΔVE_{2P} measures the difference in VE among people vaccinated in the current & two prior seasons versus those vaccinated in the current season only. Cells are coloured with increasing intensity as ΔVE estimates diverge from the null, with darker blue indicating improved VE with successive vaccinations and darker red indicating decreased VE with successive vaccinations.

[b] Castilla, 2016 restricted vaccinations to split virion vaccines in the prior seasons and subunit vaccination in the current season.

8 Meta-analysis - additional data

Additional forest plots showing the pooled estimates for the prior-only group and providing the sample counts for each study are included in this section. For all plots, the reference group is people vaccinated in neither season. Prior season is defined as the immediately prior influenza season.

$\Delta VE_{current}$ is calculated as $VE_{current\&prior}$ – $VE_{current}$ only.

ΔVE_{prior} is calculated as $VE_{current\&prior}$ – VE_{prior} only.

Random effect models for each vaccination group are presented in tables and forest plots pooled by current season and across all seasons. Fixed effect models are presented in tables for pooled estimates across all seasons. *Unadjusted VE estimates only presented in study. ‡IIV vaccine only.

For all pooled estimates, both random effect (RE) and fixed effect (FE) models were produced and are shown in tables but only RE pooled estimates are shown for subgroups in forest plots. Results are presented for all individual seasons and influenza A subtypes and influenza B of any lineage and specific lineages. The number of estimates that were included in each model are listed as N. Current is vaccination in the current season only. Prior is vaccination in the prior season only. Current&prior is vaccination in both the current & prior seasons. All are in reference to unvaccinated in both seasons. ΔVE is the difference of VE in the current & prior seasons and current season only ($\Delta VE = VE_{current\&prior} - VE_{current}$). ΔVE>0 implies higher VE when vaccinated in the current & prior seasons than in the current season alone.

For all forest plots, the following abbreviations apply:

- uvc: number of unvaccinated cases included in the estimate
- uvnc: number of unvaccinated non-cases included in the estimate
- vc: number of vaccinated cases included in the estimate
- vnc: number of vaccinated non-cases included in the estimate
- VE: vaccine effectiveness
- 95%CI: 95% confidence interval

8.1 A(H1N1)pdm09

Supplementary Table 4: A(H1N1)pdm09 random effect (upper) and fixed effect (lower) model estimates (95% CI) by season

Year	N	Current[a]	Prior	Current+prior	$\Delta VE_{current}$	ΔVE_{prior}
Random effect						
2010-2011	3	52% (32%, 66%)	53% (10%, 75%)	64% (48%, 75%)	10% (-14%, 33%)	19% (-4%, 42%)
2011-2012	2	59% (-11%, 85%)	49% (2%, 74%)	83% (65%, 92%)	27% (-29%, 83%)	36% (-5%, 77%)
2012-2013	3	48% (-32%, 80%)	-24% (-152%, 38%)	25% (-6%, 46%)	-21% (-80%, 38%)	30% (-35%, 95%)
2013-2014	5	69% (60%, 76%)	42% (29%, 53%)	64% (57%, 69%)	-7% (-17%, 3%)	21% (8%, 35%)
2015-2016	4	50% (19%, 69%)	21% (-1%, 38%)	38% (27%, 47%)	-15% (-36%, 6%)	15% (-7%, 37%)
Pooled	19	58% (48%, 66%)	33% (21%, 43%)	53% (44%, 60%)	-9% (-16%, -1%)	21% (11%, 30%)
Fixed effect						
2010-2011	3	52% (32%, 66%)	43% (21%, 59%)	64% (48%, 75%)	10% (-14%, 33%)	19% (-4%, 42%)
2011-2012	2	59% (-11%, 85%)	49% (2%, 74%)	83% (65%, 92%)	27% (-29%, 83%)	36% (-5%, 77%)
2012-2013	3	38% (-16%, 67%)	-21% (-101%, 27%)	25% (-6%, 46%)	-19% (-72%, 33%)	30% (-35%, 95%)
2013-2014	5	69% (60%, 76%)	42% (29%, 53%)	63% (57%, 69%)	-7% (-17%, 3%)	21% (8%, 35%)
2015-2016	4	45% (30%, 56%)	21% (-1%, 38%)	38% (27%, 47%)	-13% (-29%, 3%)	15% (-7%, 37%)
Pooled	19	57% (50%, 63%)	33% (24%, 41%)	51% (46%, 56%)	-9% (-16%, -1%)	21% (11%, 30%)

[a] Cells coloured blue indicate that the fixed and random effect estimates diverged by 10 percentage points or more).

Supplementary Figure 2: Pooled VE estimates by season for A(H1N1)pdm09 for people vaccinated in the current-only, prior-only and current & prior seasons

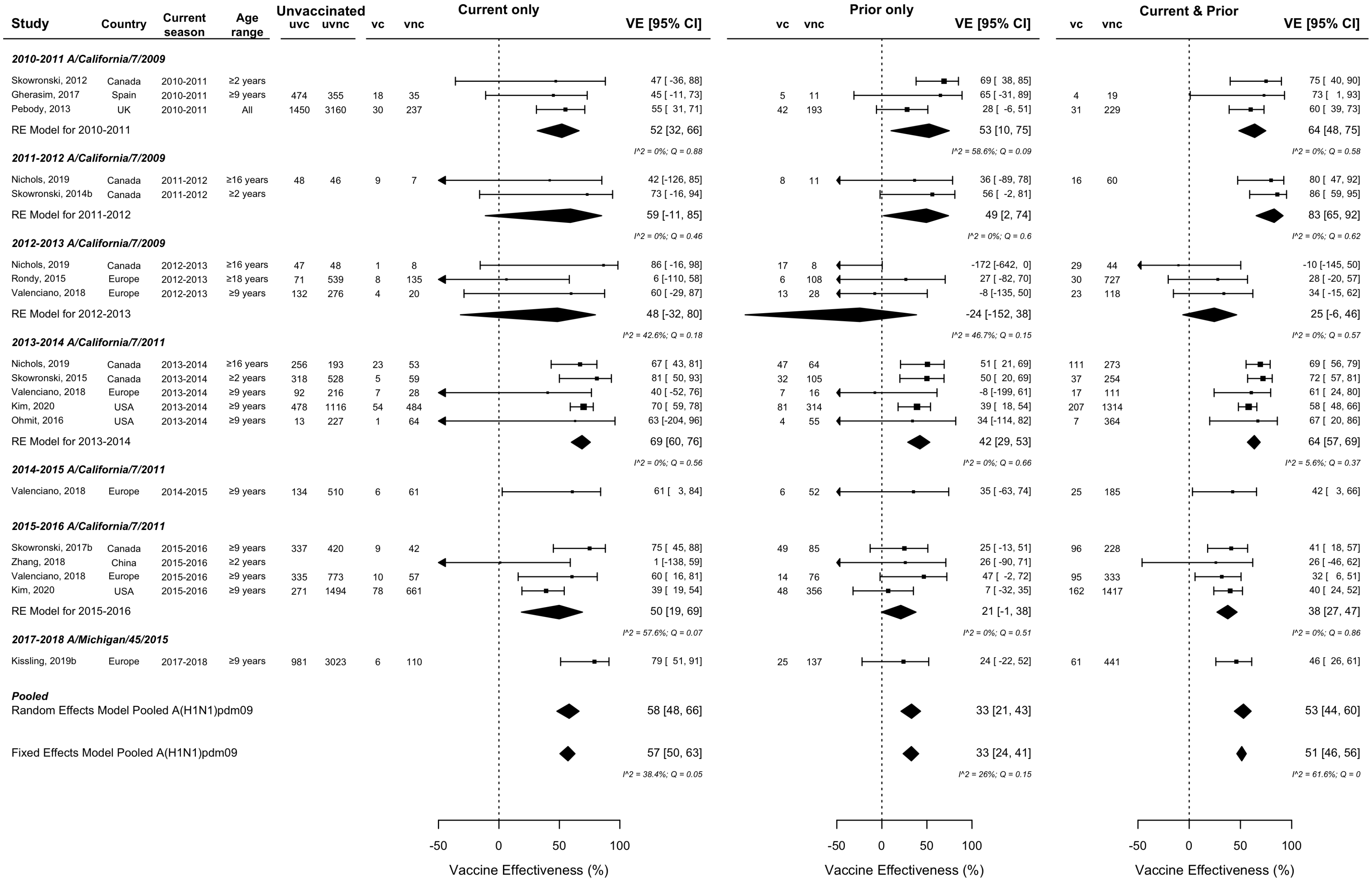

Supplementary Table 5: A(H1N1)pdm09 random effect (RE) and fixed effect (FE) model estimates (95% CI) by age group.

Year	N[a]	Current	Prior	Current+prior	$\Delta VE_{current}$	ΔVE_{prior}
Random effect						
Children	5	68% (52%, 79%)	41% (25%, 54%)	69% (56%, 78%)	1% (-18%, 21%)	26% (6%, 47%)
Older Adults	3	57% (37%, 71%)	36% (11%, 55%)	62% (51%, 71%)	6% (-14%, 26%)	24% (0%, 49%)
Fixed effect						
Children	5	68% (52%, 79%)	41% (25%, 54%)	69% (56%, 78%)	1% (-18%, 21%)	26% (6%, 47%)
Older Adults	3	57% (37%, 71%)	36% (11%, 55%)	62% (52%, 70%)	6% (-14%, 26%)	24% (0%, 49%)

[a] One estimate in children for current season only vaccination was not reported by the study (N current season only/$\Delta VE_{current}$ in children = 4).

Supplementary Figure 3: Pooled VE estimates by age group for A(H1N1)pdm09 for people vaccinated in the current-only, prior-only and current & prior seasons

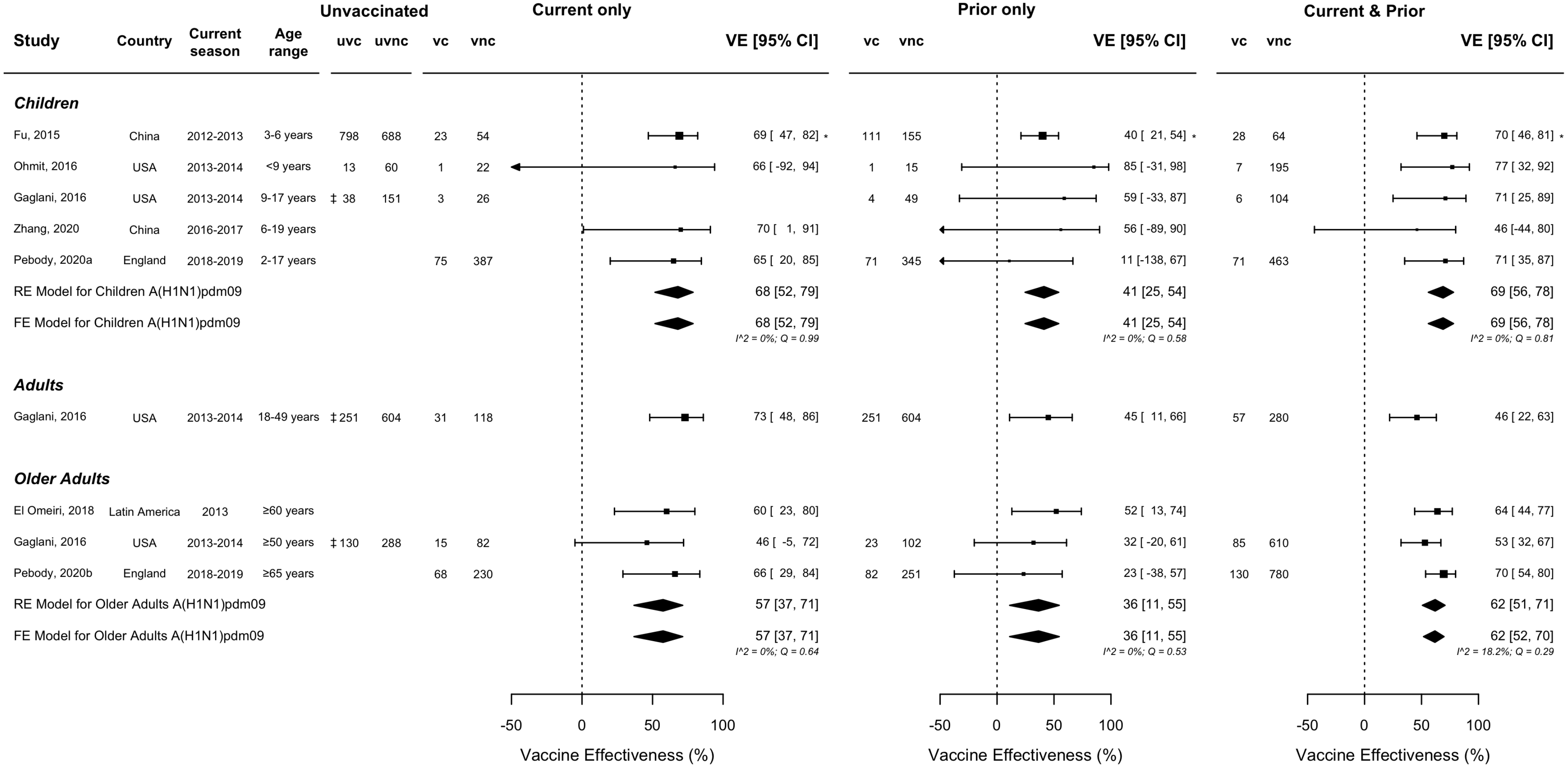

8.2 A(H3N2)

Supplementary Table 6: A(H3N2) random effect (upper) and fixed effect (lower) model estimates (95% CI) by season

Year	N[a]	Current[b]	Prior	Current+prior	$\Delta VE_{current}$	ΔVE_{prior}
Random effect						
2011-2012	4	45% (21%, 62%)	28% (-28%, 59%)	33% (15%, 47%)	-18% (-46%, 10%)	5% (-30%, 41%)
2012-2013	4	48% (36%, 57%)	10% (-45%, 45%)	37% (27%, 45%)	-12% (-26%, 3%)	28% (-12%, 68%)
2013-2014	2	35% (-36%, 69%)	38% (-60%, 76%)	36% (-1%, 60%)	3% (-61%, 67%)	8% (-82%, 99%)
2014-2015	6	36% (10%, 54%)	-7% (-45%, 21%)	-5% (-19%, 7%)	-38% (-67%, -9%)	-8% (-35%, 19%)
2016-2017	5	40% (30%, 49%)	15% (1%, 27%)	24% (12%, 34%)	-17% (-32%, -3%)	9% (-11%, 30%)
2017-2018	4	23% (2%, 40%)	-10% (-31%, 8%)	5% (-7%, 16%)	-19% (-38%, -1%)	14% (-9%, 37%)
2018-2019	2	32% (-169%, 83%)	40% (14%, 58%)	3% (-20%, 21%)	-17% (-115%, 81%)	-37% (-67%, -7%)
Pooled	30	37% (29%, 45%)	9% (-3%, 19%)	20% (12%, 27%)	-18% (-26%, -11%)	7% (-4%, 18%)
Fixed effect						
2011-2012	4	45% (21%, 62%)	21% (-10%, 43%)	33% (15%, 47%)	-18% (-46%, 10%)	3% (-26%, 32%)
2012-2013	4	48% (36%, 57%)	15% (-4%, 30%)	37% (27%, 45%)	-12% (-26%, 3%)	10% (-8%, 28%)
2013-2014	2	35% (-36%, 69%)	38% (-60%, 76%)	36% (-1%, 60%)	3% (-61%, 67%)	8% (-82%, 99%)
2014-2015	6	24% (10%, 36%)	-3% (-23%, 13%)	-5% (-18%, 7%)	-32% (-50%, -14%)	-8% (-30%, 14%)
2016-2017	5	40% (30%, 49%)	15% (1%, 27%)	24% (16%, 31%)	-17% (-30%, -5%)	8% (-8%, 23%)
2017-2018	4	21% (5%, 34%)	-10% (-31%, 8%)	5% (-7%, 16%)	-19% (-38%, -1%)	14% (-9%, 37%)
2018-2019	2	-9% (-52%, 22%)	40% (14%, 58%)	3% (-20%, 21%)	-17% (-54%, 21%)	-37% (-67%, -7%)
Pooled	30	33% (28%, 39%)	7% (0%, 14%)	18% (13%, 22%)	-17% (-24%, -11%)	5% (-4%, 13%)

[a] Three estimates included in the pooled model were single estimates for those seasons. One estimate for prior season only vaccination (2016-2017 season) was not reported by the study (N prior season only / ΔVE_{prior} 2016-2017 = 4; N prior season only/ ΔVE_{prior} pooled = 29). Cells coloured blue indicate that the fixed and random effect estimate diverged by 10 percentage points or more; cells coloured red indicate that the fixed and random effect estimates indicated opposing directions of effect.

[b] Note the discrepant FE and RE estimates for the current vaccination group, particularly 2014-2015 and 2018-2019. These seasons also had high heterogeneity indicated by I^2 (see associated forest plot).

Supplementary Figure 4: Pooled VE estimates by season for A(H3N2) for people vaccinated in the current-only, prior-only and current & prior seasons

Study	Country	Age range	Unvacc. (n/N)	Current only n/N	Current only VE (95% CI)	Prior n/N	Prior VE (95% CI)	Current & Prior n/N	Current & Prior VE (95% CI)
**2007-2008 A/Wisconsin/67/2005**									
McLean, 2014	USA	≥9 years	305/617	29/91	54 (25–71)	36/91	34 (-5–58)	99/289	43 (20–59)
**2010-2011 A/Perth/16/2009**									
Skowronski, 2017a	Canada	≥9 years	205/707	9/42	34 (-51–71)	60/164	-55 (-134–-3)	42/189	34 (-5–58)
**2011-2012 A/Perth/16/2009**									
Nichols, 2019	Canada	≥16 years	13/32	2/4	-68 (-1543–83)	8/16	-43 (-403–60)	21/79	51 (-45–83)
Skowronski, 2014b	Canada	≥2 years	NR	NR	22 (-138–74)	NR	50 (-3–76)	NR	59 (15–80)
Valenciano, 2018	Europe	≥9 years	252/555	21/57	38 (-25–69)	11/51	60 (11–82)	127/291	29 (-3–52)
Ohmit, 2014	USA	≥9 years	163/1462	22/492	54 (25–71)	32/264	-8 (-69–30)	83/872	26 (-3–47)
RE Model for 2011-2012					**45 (21–62)**		**28 (-28–59)**		**33 (15–47)**
					I^2 = 0%; Q = 0.58		*I^2 = 57.6%; Q = 0.07*		*I^2 = 0%; Q = 0.47*
**2012-2013 A/Victoria/361/2011**									
Nichols, 2019	Canada	≥16 years	271/533	28/97	59 (32–75)	128/233	-32 (-85–6)	376/877	31 (11–46)
Skowronski, 2017a	Canada	≥9 years	204/598	5/29	49 (-47–83)	34/105	0 (-66–39)	54/227	28 (-12–54)
Rondy, 2015	Europe	≥18 years	30/213	1/37	68 (-157–96)	6/46	12 (-141–68)	19/275	60 (18–80)
Kim, 2021	USA	≥9 years	482/1606	136/738	45 (31–56)	66/310	43 (23–58)	314/1371	39 (26–49)
RE Model for 2012-2013					**48 (36–57)**		**10 (-45–45)**		**37 (27–45)**
					I^2 = 0%; Q = 0.73		*I^2 = 77.6%; Q = 0*		*I^2 = 0%; Q = 0.48*
**2013-2014 A/Victoria/361/2011**									
Nichols, 2019	Canada	≥16 years	19/39	6/14	12 (-213–76)	3/10	52 (-134–90)	31/92	28 (-70–70)
Valenciano, 2018	Europe	≥9 years	98/322	8/36	45 (-39–78)	5/21	29 (-133–78)	40/151	39 (-4–64)
RE Model for 2013-2014					**35 (-36–69)**		**38 (-60–76)**		**36 (-1–60)**
					I^2 = 0%; Q = 0.57		*I^2 = 0%; Q = 0.7*		*I^2 = 0%; Q = 0.75*
**2014-2015 A/Texas/50/2012**									
Nichols, 2019	Canada	≥16 years	90/192	18/47	35 (-33–68)	47/71	-121 (-293–-25)	216/450	-8 (-56–25)
Skowronski, 2017a	Canada	≥9 years	230/698	9/71	65 (25–83)	57/158	-7 (-59–28)	194/489	-33 (-78–1)
Valenciano, 2018	Europe	≥9 years	378/935	30/101	48 (15–68)	25/94	15 (-46–50)	154/375	5 (-28–30)
Kim, 2021	USA	≥9 years	458/2103	171/898	11 (-11–28)	132/587	4 (-21–25)	576/2561	-2 (-20–14)
Petrie, 2016	USA	≥18 years	18/96	11/107	55 (-9–82)	7/27	-50 (-372–52)	33/107	42 (-27–73)
Petrie, 2017	USA	≥9 years	26/247	8/89	-5 (-133–53) *	7/78	44 (-29–76) *	58/566	-11 (-89–35) *
RE Model for 2014-2015					**36 (10–54)**		**-7 (-45–21)**		**-5 (-19–7)**
					I^2 = 53.1%; Q = 0.06		*I^2 = 50.8%; Q = 0.07*		*I^2 = 6.4%; Q = 0.38*
**2015-2016 A/Switzerland/9715293/2013**									
Zhang, 2018	China	≥2 years	NR	NR	62 (-25–88)	NR	-8 (-114–46)	NR	51 (0–76)
**2016-2017 & 2017 A/Hong Kong/4801/2014**									
Skowronski, 2022	Canada	≥9 years	344/871	19/62	33 (-21–62)	71/178	1 (-41–30)	116/426	43 (24–58)
Kissling, 2019b	Europe	≥9 years	3168/6511	69/201	50 (32–63)	87/231	29 (5–47)	412/879	20 (5–32)
Pebody, 2017	UK	≥18 years	NR	NR	43 (-28–75) *	NR	NR	NR	21 (-12–45) *
Kim, 2021	USA	≥9 years	467/1820	130/708	35 (18–48)	106/444	15 (-9–34)	399/1804	27 (13–38)
Sullivan, 2017	Australia	All	294/935	14/73	43 (1–71)	47/179	4 (-40–36)	155/550	3 (-29–27)
RE Model for 2016-2017					**40 (30–49)**		**15 (1–27)**		**24 (12–34)**
					I^2 = 0%; Q = 0.73		*I^2 = 0%; Q = 0.46*		*I^2 = 43.2%; Q = 0.13*
**2017-2018 A/Hong Kong/4801/2014**									
Skowronski, 2022	Canada	≥9 years	283/1032	12/71	45 (-7–71)	68/217	-22 (-71–13)	164/619	9 (-18–30)
Kissling, 2019b	Europe	≥9 years	491/3274	11/116	49 (1–74)	17/136	16 (-47–52)	95/525	7 (-29–33)
Pebody, 2019	UK	≥18 years	189/932	20/96	-3 (-86–43)	16/112	13 (-68–55)	94/404	-9 (-65–28)
Kim, 2021	USA	≥9 years	571/2038	181/817	17 (-2–32)	133/449	-13 (-43–11)	490/1979	5 (-12–19)
RE Model for 2017-2018					**23 (2–40)**		**-10 (-31–8)**		**5 (-7–16)**
					I^2 = 21%; Q = 0.28		*I^2 = 0%; Q = 0.62*		*I^2 = 0%; Q = 0.9*
**2018-2019 A/Singapore/INFIMH16-0019/2016**									
Skowronski, 2019	Canada	≥9 years	158/862	5/60	69 (18–89)	21/190	33 (-13–61)	83/459	-1 (-45–29)
Kissling, 2019a	Europe	≥9 years	1147/3208	76/175	-27 (-81–11)	25/138	45 (11–66)	153/462	5 (-23–27)
RE Model for 2018-2019					**32 (-169–83)**		**40 (14–58)**		**3 (-20–21)**
					I^2 = 85.1%; Q = 0.01		*I^2 = 0%; Q = 0.59*		*I^2 = 0%; Q = 0.79*
**Pooled**									
Random Effects Model Pooled A(H3N2)					**37 (29–45)**		**9 (-3–19)**		**20 (12–27)**
					I^2 = 45%; Q = 0		*I^2 = 51.7%; Q = 0*		*I^2 = 58.4%; Q = 0*
Fixed Effects Model Pooled A(H3N2)					**33 (28–39)**		**7 (0–14)**		**18 (13–22)**
					I^2 = 45%; Q = 0		*I^2 = 51.7%; Q = 0*		*I^2 = 58.4%; Q = 0*

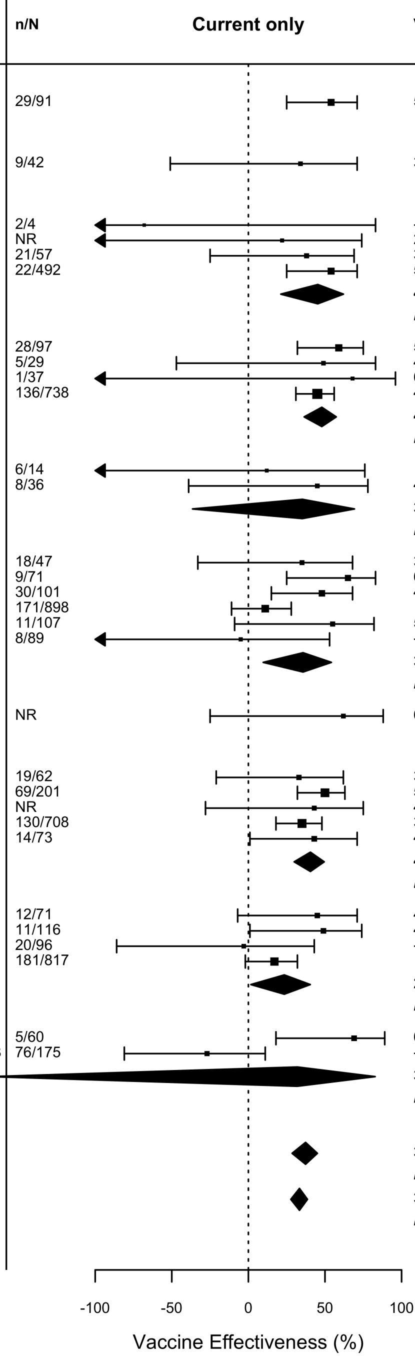

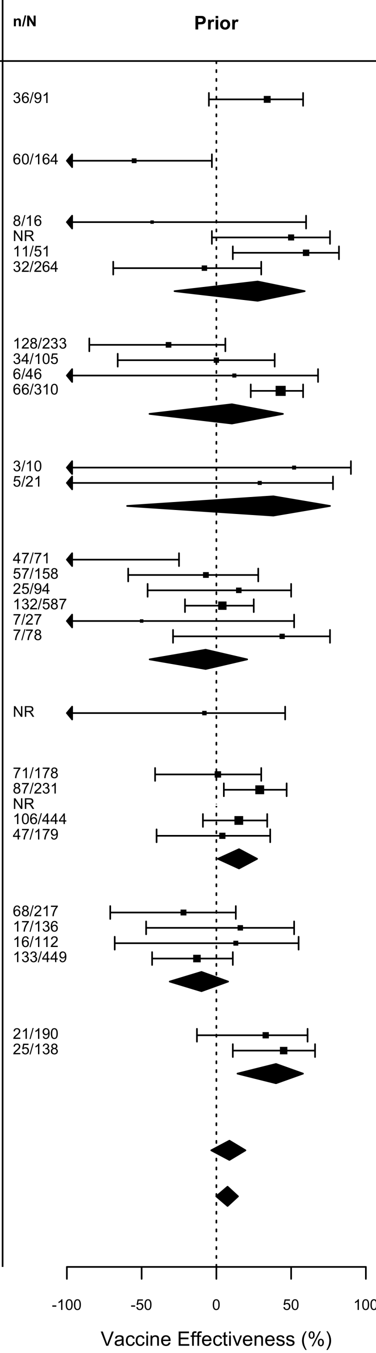

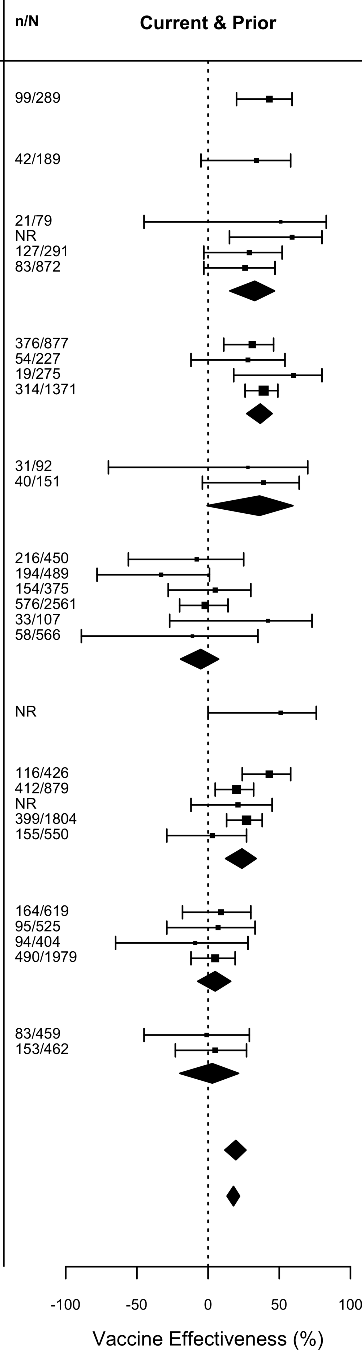

Supplementary Table 7: A(H3N2) random effect (RE) and fixed effect (FE) model estimates (95% CI) by age group

Year	N[a]	Current	Prior[b]	Current+prior	$\Delta VE_{current}$	ΔVE_{prior}
Random effect						
Children	7	29% (-45%, 65%)	-8% (-51%, 23%)	40% (19%, 56%)	14% (-35%, 62%)	52% (13%, 91%)
Adults	2	32% (-222%, 86%)	25% (-34%, 58%)	-21% (-55%, 5%)	-36% (-163%, 90%)	-47% (-88%, -6%)
Older Adults	3	29% (2%, 48%)	6% (-65%, 46%)	19% (4%, 32%)	-10% (-42%, 21%)	4% (-35%, 44%)
Fixed effect						
Children	7	27% (-2%, 48%)	-16% (-47%, 9%)	41% (23%, 54%)	3% (-29%, 35%)	54% (21%, 87%)
Adults	2	-5% (-55%, 29%)	22% (-11%, 46%)	-21% (-55%, 5%)	-55% (-99%, -11%)	-47% (-88%, -6%)
Older Adults	3	29% (2%, 48%)	11% (-20%, 34%)	19% (4%, 32%)	-13% (-40%, 14%)	1% (-28%, 31%)

[a] One estimate in children for current season only vaccination was not reported by that study (N current season only / $\Delta VE_{current}$ in children = 6). Three estimates in children for prior season only vaccination were not reported by those studies (N prior season only / ΔVE_{prior} in children = 4). Cells red indicate that the fixed and random effect estimates indicated opposing directions of effect.

[b] Note the divergent FE and RE estimates for the prior vaccination group. These also had high heterogeneity indicated by I^2 (see forest plots).

Supplementary Figure 5: Pooled VE estimates by age group for A(H3N2) for people vaccinated in the current-only, prior-only and current & prior seasons

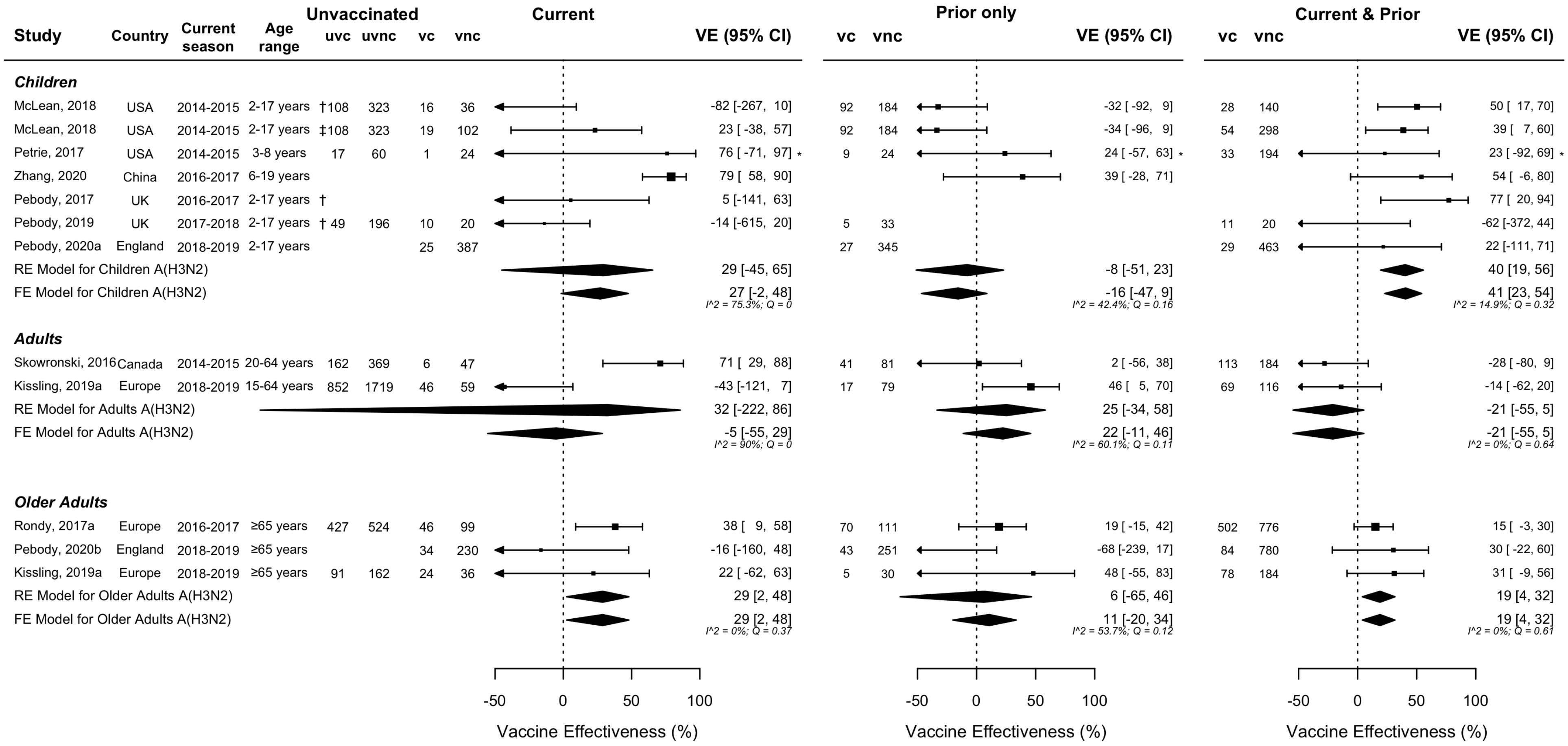

8.3 Influenza B

Supplementary Table 8: B (any lineage) random effect (upper) and fixed effect (lower) model estimates (95% CI) by season

Year[a]	N	Current	Prior	Current+prior	$\Delta VE_{current}$	ΔVE_{prior}
Random effect						
2010-2011	2	56% (29%, 72%)	-7% (-54%, 26%)	54% (31%, 70%)	-2% (-32%, 29%)	60% (14%, 105%)
2011-2012	2	29% (-44%, 65%)	28% (-43%, 64%)	48% (23%, 65%)	19% (-22%, 60%)	18% (-13%, 50%)
2012-2013	5	62% (51%, 71%)	25% (-6%, 46%)	56% (43%, 66%)	-3% (-16%, 10%)	24% (6%, 42%)
2013-2014	2	78% (35%, 93%)	18% (-31%, 48%)	52% (14%, 73%)	-26% (-48%, -3%)	37% (-7%, 81%)
2014-2015	4	57% (41%, 69%)	26% (0%, 46%)	46% (33%, 56%)	-11% (-30%, 7%)	20% (-6%, 45%)
2015-2016	3	58% (42%, 69%)	12% (-24%, 37%)	37% (12%, 55%)	-14% (-33%, 5%)	26% (0%, 53%)
2016-2017	2	53% (36%, 66%)	22% (-16%, 48%)	50% (30%, 64%)	-7% (-26%, 13%)	28% (4%, 52%)
2017-2018	2	44% (30%, 56%)	23% (-2%, 41%)	33% (-10%, 59%)	-2% (-18%, 15%)	6% (-48%, 61%)
Pooled	22	54% (49%, 59%)	21% (12%, 29%)	47% (41%, 53%)	-7% (-14%, 0%)	25% (16%, 34%)
Pooled Vic antigen in TIV	8	47% (38%, 55%)	19% (6%, 31%)	45% (35%, 54%)	-2% (-13%, 9%)	26% (10%, 41%)
Pooled Yam antigen in TIV	14	61% (54%, 67%)	23% (11%, 34%)	48% (39%, 55%)	-10% (-19%, -2%)	24% (12%, 37%)
Fixed effect						
2010-2011	2	56% (29%, 72%)	-7% (-54%, 26%)	54% (31%, 70%)	-2% (-32%, 29%)	60% (14%, 105%)
2011-2012	2	27% (-22%, 57%)	29% (-5%, 52%)	47% (29%, 60%)	19% (-22%, 60%)	18% (-13%, 50%)
2012-2013	5	62% (51%, 71%)	29% (9%, 45%)	57% (49%, 64%)	-3% (-16%, 10%)	24% (6%, 42%)
2013-2014	2	74% (50%, 86%)	18% (-31%, 48%)	48% (30%, 61%)	-26% (-48%, -3%)	37% (-7%, 81%)
2014-2015	4	57% (41%, 69%)	26% (0%, 46%)	46% (34%, 56%)	-11% (-30%, 7%)	20% (-6%, 45%)
2015-2016	3	58% (42%, 69%)	14% (-14%, 35%)	39% (25%, 50%)	-14% (-33%, 5%)	26% (0%, 53%)
2016-2017	2	53% (36%, 66%)	22% (-16%, 48%)	48% (36%, 57%)	-7% (-26%, 12%)	28% (4%, 52%)
2017-2018	2	44% (30%, 56%)	23% (-2%, 41%)	40% (28%, 49%)	-2% (-18%, 15%)	18% (-6%, 42%)
Pooled	22	54% (49%, 59%)	21% (12%, 29%)	47% (43%, 51%)	-7% (-14%, 0%)	25% (16%, 34%)
Pooled Vic antigen in TIV	8	47% (38%, 55%)	19% (6%, 31%)	45% (38%, 51%)	-2% (-13%, 9%)	26% (11%, 40%)
Pooled Yam antigen in TIV	14	61% (54%, 67%)	23% (11%, 34%)	49% (43%, 54%)	-10% (-19%, -2%)	24% (12%, 37%)

[a] A B/Victoria antigen was included in trivalent influenza vaccines (TIV) in 2010-2011, 2011-2012, 2016-2017, 2017-2018, while a B/Yamagata antigen was included in other years.

Supplementary Figure 6: Pooled VE estimates by season for Influenza B for people vaccinated in the current-only, prior-only and current & prior seasons

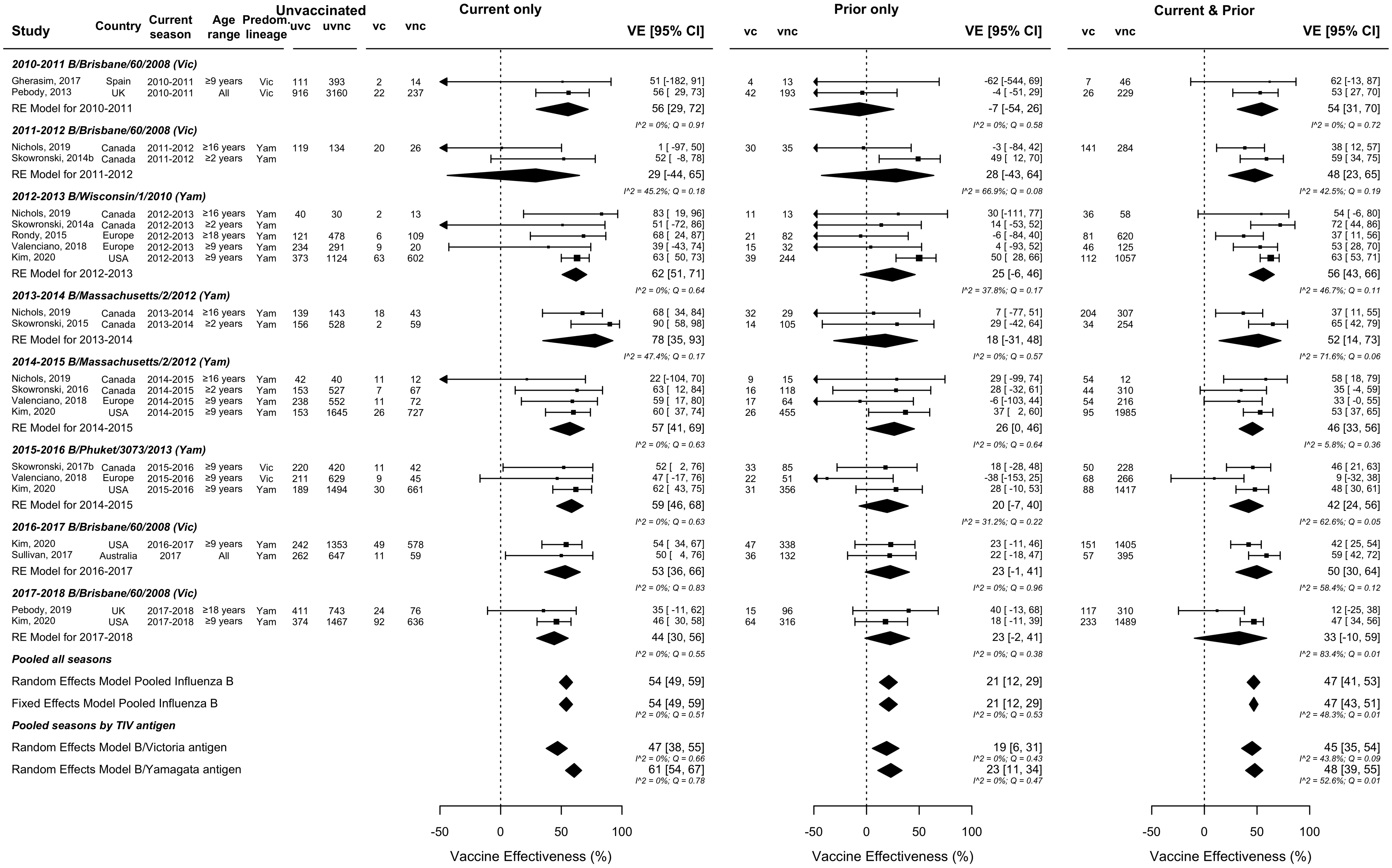

Study	Country	Current season	Age range	Predom. lineage	Unvaccinated uvc	Unvaccinated uvnc	Current only vc	Current only vnc	Current only VE [95% CI]	Prior only vc	Prior only vnc	Prior only VE [95% CI]	Current & Prior vc	Current & Prior vnc	Current & Prior VE [95% CI]
2010-2011 B/Brisbane/60/2008 (Vic)															
Gherasim, 2017	Spain	2010-2011	≥9 years	Vic	111	393	2	14	51 [-182, 91]	4	13	-62 [-544, 69]	7	46	62 [-13, 87]
Pebody, 2013	UK	2010-2011	All	Vic	916	3160	22	237	56 [29, 73]	42	193	-4 [-51, 29]	26	229	53 [27, 70]
RE Model for 2010-2011									56 [29, 72]			-7 [-54, 26]			54 [31, 70]
									I^2 = 0%; Q = 0.91			*I^2 = 0%; Q = 0.58*			*I^2 = 0%; Q = 0.72*
2011-2012 B/Brisbane/60/2008 (Vic)															
Nichols, 2019	Canada	2011-2012	≥16 years	Yam	119	134	20	26	1 [-97, 50]	30	35	-3 [-84, 42]	141	284	38 [12, 57]
Skowronski, 2014b	Canada	2011-2012	≥2 years	Yam					52 [-8, 78]			49 [12, 70]			59 [34, 75]
RE Model for 2011-2012									29 [-44, 65]			28 [-43, 64]			48 [23, 65]
									I^2 = 45.2%; Q = 0.18			*I^2 = 66.9%; Q = 0.08*			*I^2 = 42.5%; Q = 0.19*
2012-2013 B/Wisconsin/1/2010 (Yam)															
Nichols, 2019	Canada	2012-2013	≥16 years	Yam	40	30	2	13	83 [19, 96]	11	13	30 [-111, 77]	36	58	54 [-6, 80]
Skowronski, 2014a	Canada	2012-2013	≥2 years	Yam					51 [-72, 86]			14 [-53, 52]			72 [44, 86]
Rondy, 2015	Europe	2012-2013	≥18 years	Yam	121	478	6	109	68 [24, 87]	21	82	-6 [-84, 40]	81	620	37 [11, 56]
Valenciano, 2018	Europe	2012-2013	≥9 years	Yam	234	291	9	20	39 [-43, 74]	15	32	4 [-93, 52]	46	125	53 [28, 70]
Kim, 2020	USA	2012-2013	≥9 years	Yam	373	1124	63	602	63 [50, 73]	39	244	50 [28, 66]	112	1057	63 [53, 71]
RE Model for 2012-2013									62 [51, 71]			25 [-6, 46]			56 [43, 66]
									I^2 = 0%; Q = 0.64			*I^2 = 37.8%; Q = 0.17*			*I^2 = 46.7%; Q = 0.11*
2013-2014 B/Massachusetts/2/2012 (Yam)															
Nichols, 2019	Canada	2013-2014	≥16 years	Yam	139	143	18	43	68 [34, 84]	32	29	7 [-77, 51]	204	307	37 [11, 55]
Skowronski, 2015	Canada	2013-2014	≥2 years	Yam	156	528	2	59	90 [58, 98]	14	105	29 [-42, 64]	34	254	65 [42, 79]
RE Model for 2013-2014									78 [35, 93]			18 [-31, 48]			52 [14, 73]
									I^2 = 47.4%; Q = 0.17			*I^2 = 0%; Q = 0.57*			*I^2 = 71.6%; Q = 0.06*
2014-2015 B/Massachusetts/2/2012 (Yam)															
Nichols, 2019	Canada	2014-2015	≥16 years	Yam	42	40	11	12	22 [-104, 70]	9	15	29 [-99, 74]	54	12	58 [18, 79]
Skowronski, 2016	Canada	2014-2015	≥2 years	Yam	153	527	7	67	63 [12, 84]	16	118	28 [-32, 61]	44	310	35 [-4, 59]
Valenciano, 2018	Europe	2014-2015	≥9 years	Yam	238	552	11	72	59 [17, 80]	17	64	-6 [-103, 44]	54	216	33 [-0, 55]
Kim, 2020	USA	2014-2015	≥9 years	Yam	153	1645	26	727	60 [37, 74]	26	455	37 [2, 60]	95	1985	53 [37, 65]
RE Model for 2014-2015									57 [41, 69]			26 [0, 46]			46 [33, 56]
									I^2 = 0%; Q = 0.63			*I^2 = 0%; Q = 0.64*			*I^2 = 5.8%; Q = 0.36*
2015-2016 B/Phuket/3073/2013 (Yam)															
Skowronski, 2017b	Canada	2015-2016	≥9 years	Vic	220	420	11	42	52 [2, 76]	33	85	18 [-28, 48]	50	228	46 [21, 63]
Valenciano, 2018	Europe	2015-2016	≥9 years	Vic	211	629	9	45	47 [-17, 76]	22	51	-38 [-153, 25]	68	266	9 [-32, 38]
Kim, 2020	USA	2015-2016	≥9 years	Yam	189	1494	30	661	62 [43, 75]	31	356	28 [-10, 53]	88	1417	48 [30, 61]
RE Model for 2014-2015									59 [46, 68]			20 [-7, 40]			42 [24, 56]
									I^2 = 0%; Q = 0.63			*I^2 = 31.2%; Q = 0.22*			*I^2 = 62.6%; Q = 0.05*
2016-2017 B/Brisbane/60/2008 (Vic)															
Kim, 2020	USA	2016-2017	≥9 years	Yam	242	1353	49	578	54 [34, 67]	47	338	23 [-11, 46]	151	1405	42 [25, 54]
Sullivan, 2017	Australia	2017	All	Yam	262	647	11	59	50 [4, 76]	36	132	22 [-18, 47]	57	395	59 [42, 72]
RE Model for 2016-2017									53 [36, 66]			23 [-1, 41]			50 [30, 64]
									I^2 = 0%; Q = 0.83			*I^2 = 0%; Q = 0.96*			*I^2 = 58.4%; Q = 0.12*
2017-2018 B/Brisbane/60/2008 (Vic)															
Pebody, 2019	UK	2017-2018	≥18 years	Yam	411	743	24	76	35 [-11, 62]	15	96	40 [-13, 68]	117	310	12 [-25, 38]
Kim, 2020	USA	2017-2018	≥9 years	Yam	374	1467	92	636	46 [30, 58]	64	316	18 [-11, 39]	233	1489	47 [34, 56]
RE Model for 2017-2018									44 [30, 56]			23 [-2, 41]			33 [-10, 59]
									I^2 = 0%; Q = 0.55			*I^2 = 0%; Q = 0.38*			*I^2 = 83.4%; Q = 0.01*
Pooled all seasons															
Random Effects Model Pooled Influenza B									54 [49, 59]			21 [12, 29]			47 [41, 53]
Fixed Effects Model Pooled Influenza B									54 [49, 59]			21 [12, 29]			47 [43, 51]
									I^2 = 0%; Q = 0.51			*I^2 = 0%; Q = 0.53*			*I^2 = 48.3%; Q = 0.01*
Pooled seasons by TIV antigen															
Random Effects Model B/Victoria antigen									47 [38, 55]			19 [6, 31]			45 [35, 54]
									I^2 = 0%; Q = 0.66			*I^2 = 0%; Q = 0.43*			*I^2 = 43.8%; Q = 0.09*
Random Effects Model B/Yamagata antigen									61 [54, 67]			23 [11, 34]			48 [39, 55]
									I^2 = 0%; Q = 0.78			*I^2 = 0%; Q = 0.47*			*I^2 = 52.6%; Q = 0.01*

Supplementary Table 9: B/Victoria lineage infection random effect (upper) and fixed effect (lower) model estimates (95% CI) by season.

Year[a]	N	Current	Prior	Current+prior	$\Delta VE_{current}$	ΔVE_{prior}
Random effect						
2012-2013	2	64% (34%, 80%)	59% (16%, 80%)	62% (42%, 75%)	-3% (-33%, 28%)	3% (-42%, 48%)
2015-2016	2	56% (28%, 74%)	14% (-28%, 42%)	42% (18%, 58%)	-16% (-50%, 18%)	30% (-12%, 71%)
Pooled	5	61% (43%, 73%)	31% (0%, 53%)	52% (38%, 63%)	-10% (-31%, 12%)	15% (-10%, 41%)
Fixed effect						
2012-2013	2	64% (34%, 80%)	60% (28%, 78%)	62% (42%, 75%)	-3% (-33%, 28%)	-3% (-33%, 27%)
2015-2016	2	56% (28%, 74%)	14% (-28%, 42%)	42% (18%, 58%)	-16% (-50%, 18%)	30% (-12%, 71%)
Pooled	5	61% (43%, 73%)	29% (5%, 47%)	52% (38%, 62%)	-10% (-31%, 12%)	13% (-10%, 36%)

[a] Two estimates included in the pooled model were single estimates for those seasons.

Supplementary Table 10: B/Yamagata lineage infection random effect (upper) and fixed effect (lower) model estimates (95% CI) by season

x Year[a]	N	Current	Prior	Current+prior	$\Delta VE_{current}$	ΔVE_{prior}
Random effect						
2012-2013	2	67% (50%, 78%)	29% (-56%, 67%)	63% (50%, 73%)	-6% (-25%, 13%)	39% (-32%, 110%)
2014-2015	3	58% (36%, 72%)	46% (18%, 65%)	41% (9%, 62%)	-5% (-28%, 18%)	5% (-24%, 33%)
Pooled	9	56% (39%, 68%)	38% (25%, 49%)	52% (42%, 60%)	-5% (-17%, 6%)	14% (0%, 28%)
Fixed effect						
2012-2013	2	67% (50%, 78%)	38% (11%, 57%)	63% (50%, 73%)	-6% (-25%, 13%)	19% (-6%, 43%)
2014-2015	3	58% (36%, 72%)	46% (18%, 65%)	47% (31%, 59%)	-5% (-28%, 18%)	5% (-24%, 33%)
Pooled	9	57% (46%, 65%)	38% (25%, 49%)	52% (45%, 58%)	-5% (-17%, 6%)	14% (0%, 28%)

[a] Four estimates included in the pooled model were single estimates for those seasons. One estimate for current season only vaccination in 2014-2015 was not reported by the study (N current season only /$\Delta VE_{current}$ 2014-2015 season = 2; N current season only / $\Delta VE_{current}$ pooled = 8).

Supplementary Figure 7: Pooled VE estimates by influenza B lineage of infection for people vaccinated in the current-only, prior-only and current & prior seasons

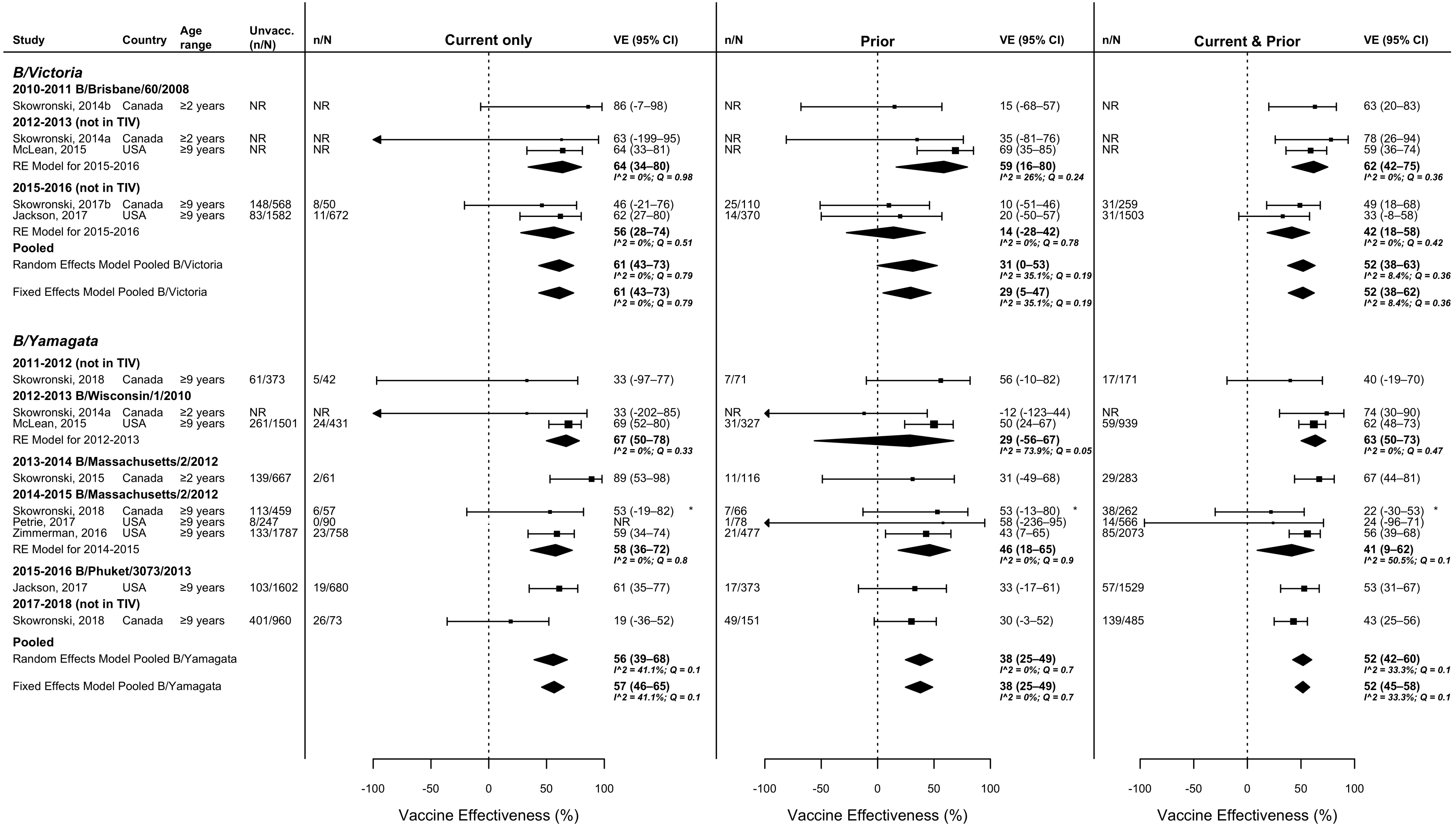

Supplementary Table 11: B (any lineage) random effect (RE) and fixed effect (FE) model estimates (95% CI) by season

Year[a]	N	Current	Prior	Current+prior	$\Delta VE_{current}$	ΔVE_{prior}
Random effect						
Children	2		42% (9%, 63%)	60% (39%, 74%)		20% (-13%, 53%)
Adults	2	39% (-3%, 64%)	57% (12%, 79%)	40% (3%, 63%)	2% (-38%, 41%)	-17% (-46%, 11%)
Older Adults	3	-6% (-99%, 43%)	-5% (-115%, 49%)	-6% (-75%, 35%)	-2% (-95%, 91%)	5% (-108%, 117%)
Fixed effect						
Children	2		42% (9%, 63%)	60% (39%, 74%)		20% (-13%, 53%)
Adults	2	39% (-3%, 64%)	55% (26%, 73%)	39% (17%, 55%)	2% (-38%, 41%)	-17% (-46%, 11%)
Older Adults	3	-6% (-99%, 43%)	-5% (-115%, 49%)	-6% (-75%, 35%)	-2% (-95%, 91%)	5% (-108%, 117%)

[a] One estimate in children for current season only vaccination was not reported by that study (N estimates children current season only = 1, therefore no model estimated for current season only / $\Delta VE_{current}$ in children).

Supplementary Figure 8: Pooled VE estimates against influenza B by age group for people vaccinated in the current-only, prior-only and current & prior seasons

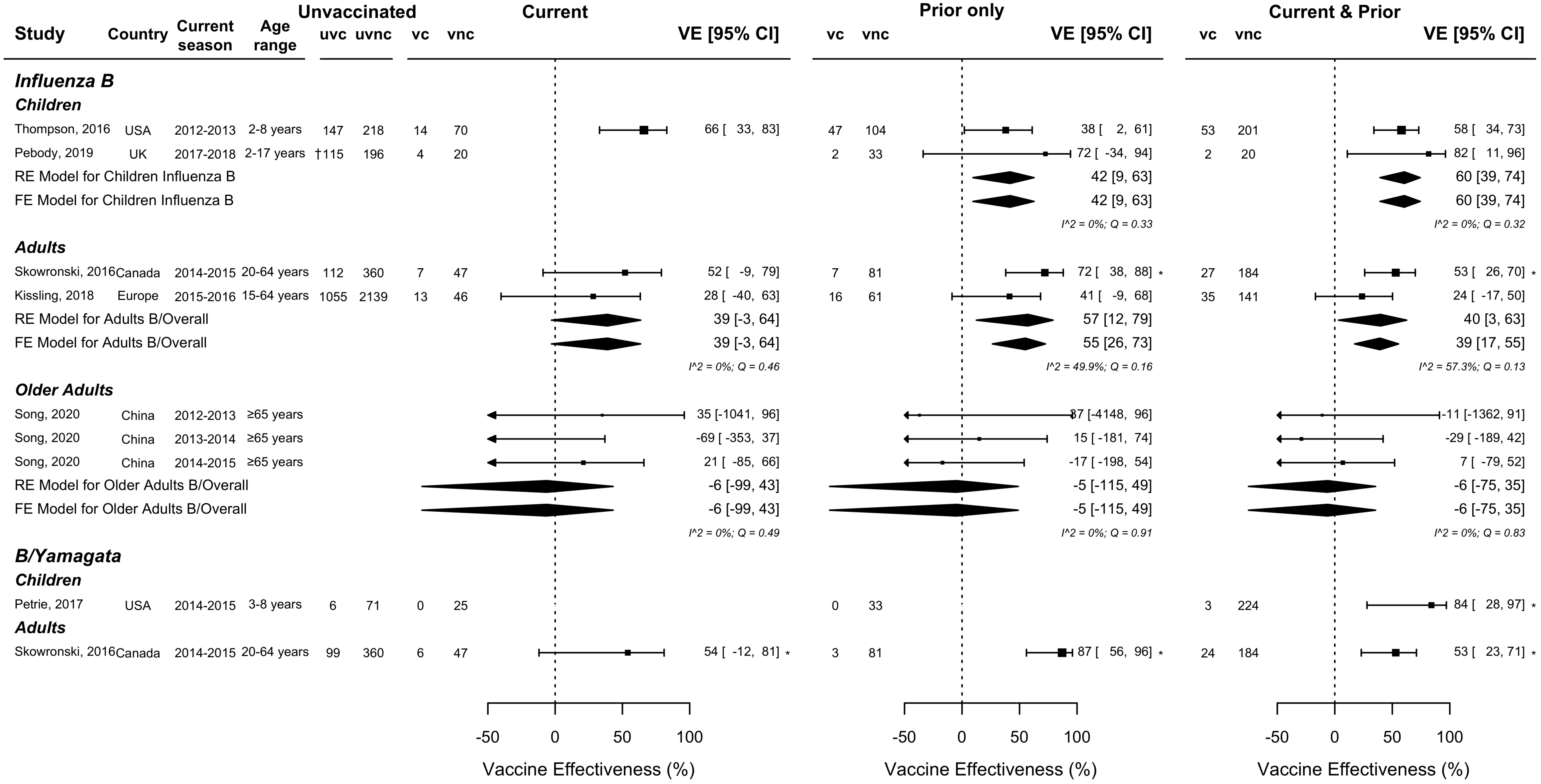

9 Risk of bias

Studies were assessed using the Risk Of Bias In Non-randomized Studies - of Interventions (ROBINS-I).

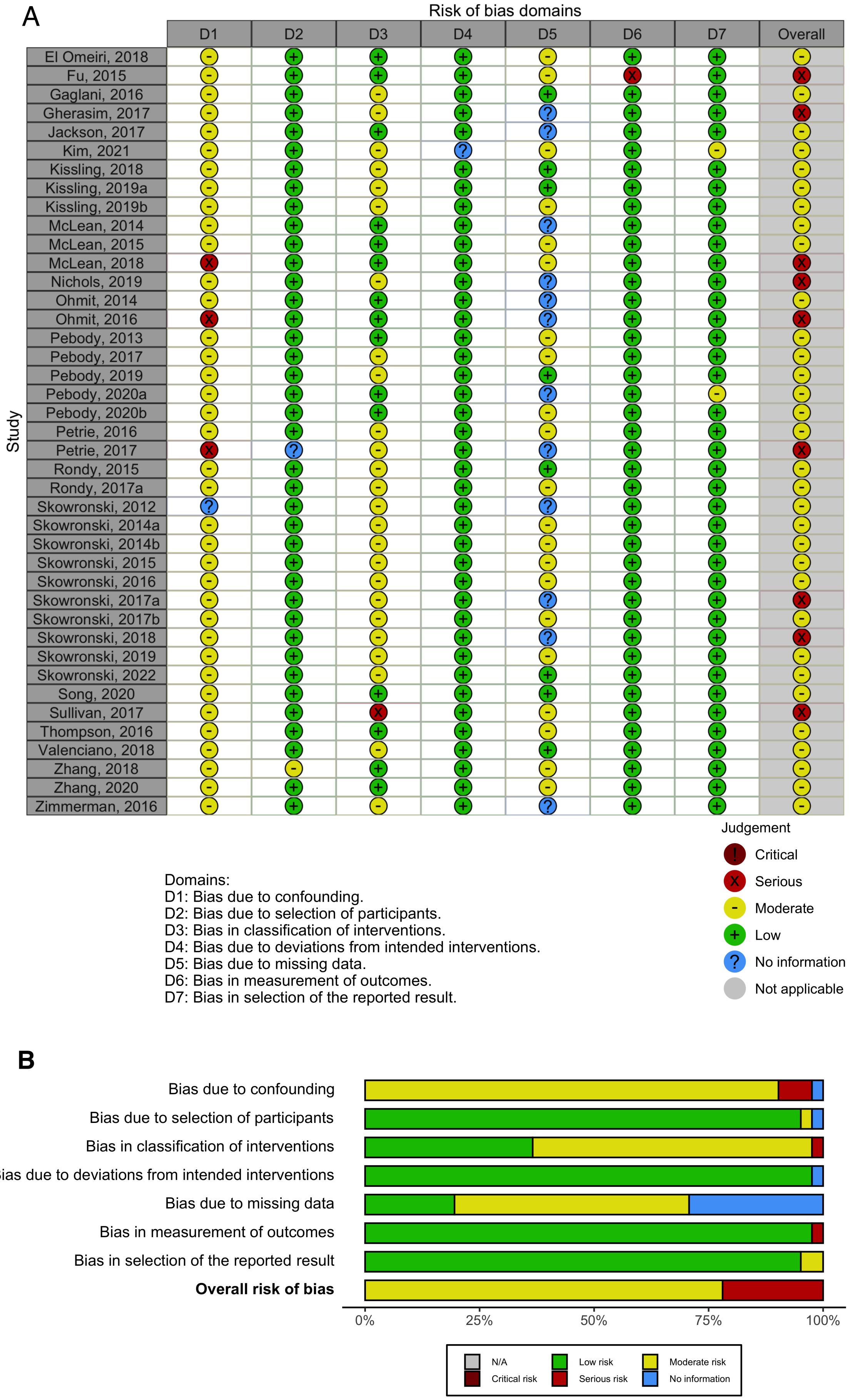

10 Publication bias

Supplementary Figure 9: Funnel plots for each pooled analysis by subtype/lineage

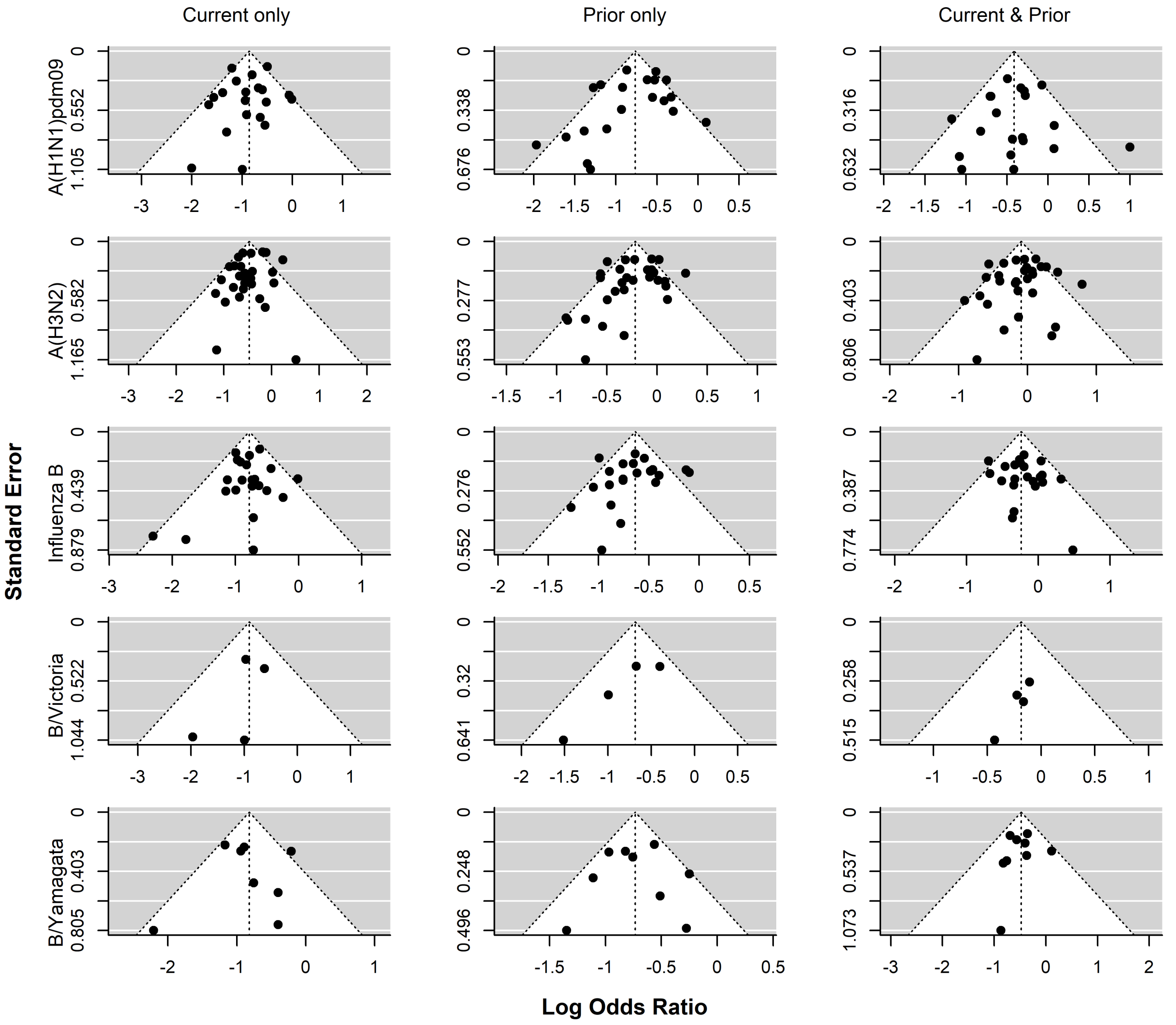

Note that estimates for B/Victoria and B/Yamagata include fewer than 10 observations, which is lower than the number recommended by Cochrane for statistical testing (`https://methods.cochrane.org/bias/reporting-biases`).

Supplementary Table 12: Egger's test for publication bias

Estimate	Vaccination group	Egger's test value (95% CI)	p-value[a]
A(H1N1)pdm09	Current only	-0.76% (-1.24%; -0.28%)	0.61
A(H1N1)pdm09	Prior only	-0.44% (-0.84%; -0.03%)	0.84
A(H1N1)pdm09	Current and prior	-0.47% (-0.85%; -0.09%)	0.1
A(H3N2)	Current only	-0.33% (-0.58%; -0.08%)	0.23
A(H3N2)	Prior only	0.01% (-0.27%; 0.3%)	0.41
A(H3N2)	Current and prior	-0.02% (-0.22%; 0.19%)	0.03
Influenza B	Current only	-0.71% (-0.96%; -0.46%)	0.55
Influenza B	Prior only	-0.39% (-0.72%; -0.06%)	0.35
Influenza B	Current and prior	-0.71% (-0.96%; -0.46%)	0.23

[a] Note that estimates for B/Victoria and B/Yamagata include fewer than 10 observations, which is lower than the number recommended by Cochrane for statistical testing (`https://methods.cochrane.org/bias/reporting-biases`). Statistical tests are therefore not provided for these viruses.

11 Sensitivity analyses

Vaccine effectiveness (VE) random effect (RE) and fixed effect (FE) models produced by meta-analyses. Results are presented for all individual seasons and influenza A subtypes and influenza B of any lineage and specific lineages. The number of estimates that were included in each model are listed as N. Current is vaccination in the current season only. Prior is vaccination in the prior season only. Current and prior is vaccination in both the current & prior seasons. All are in reference to unvaccinated in both seasons. ΔVE is the difference of VE in the current & prior seasons and current season only ($\Delta VE = VE_{C+P} \breve{} VE_C$). ΔVE>0 implies higher VE when vaccinated in the current & prior seasons than in the current season alone.

11.1 Inclusion of non-PCR diagnostics tests

Studies that utilised rapid diagnostic influenza tests as the diagnostic confirmation method were not included in the main analysis. One study with seasonal influenza B VE estimates (Shinjoh, 2018; current season studied was 2016-2017) was eligible to be added but made no impact on the pooled estimates.

Supplementary Table 13: Sensitivity of pooled estimates including studies that used RIDT, influenza B

	Original model			Sensitivity model		
	N	Random Effect	Fixed Effect	N	Random Effect	Fixed Effect
Current only	22	54% (49%, 59%)	54% (49%, 59%)	23	54% (49%, 59%)	54% (49%, 59%)
Prior only	22	21% (12%, 29%)	21% (12%, 29%)	23	21% (12%, 29%)	21% (12%, 29%)
Current and prior	22	47% (41%, 53%)	47% (43%, 51%)	23	47% (40%, 52%)	47% (42%, 51%)
Delta	22	-7% (-14%, 0%)	-7% (-14%, 0%)	23	-8% (-14%, -1%)	-8% (-14%, -1%)

11.2 Removal of studies with serious/critical/no information risk of bias overall

Studies deemed at serious/critical/no information overall risk of bias by the ROBINS-I method were removed and pooled estimates recalculated. Nine studies were judged at serious overall risk of bias. Their removal had minimal impact on pooled estimates.

Supplementary Table 14: Sensitivity of pooled estimates excluding studies at risk of bias, A(H1N1)pdm09

	Original model			Sensitivity model		
	N	Random Effect	Fixed Effect	N	Random Effect	Fixed Effect
Current only	19	58% (48%, 66%)	57% (50%, 63%)	14	58% (45%, 68%)	57% (49%, 63%)
Prior only	19	33% (21%, 43%)	33% (24%, 41%)	14	32% (22%, 42%)	32% (22%, 41%)
Current and prior	19	53% (44%, 60%)	51% (46%, 56%)	14	50% (41%, 58%)	49% (44%, 54%)
Delta	19	-9% (-16%, -1%)	-9% (-16%, -1%)	14	-11% (-19%, -2%)	-11% (-19%, -2%)

Supplementary Table 15: Sensitivity of pooled estimates excluding studies at risk of bias, A(H3N2)

	Original model			Sensitivity model		
	N	Random Effect	Fixed Effect	N	Random Effect	Fixed Effect
Current only	30	37% (29%, 45%)	33% (28%, 39%)	21	36% (26%, 45%)	32% (26%, 38%)
Prior only	29	9% (-3%, 19%)	7% (0%, 14%)	20	17% (5%, 27%)	14% (6%, 22%)
Current and prior	30	20% (12%, 27%)	18% (13%, 22%)	21	22% (14%, 30%)	19% (15%, 24%)
Delta	30	-18% (-26%, -11%)	-17% (-24%, -11%)	21	-15% (-22%, -7%)	-15% (-22%, -7%)

Supplementary Table 16: Sensitivity of pooled estimates excluding studies at risk of bias, influenza B

	Original model			Sensitivity model		
	N	Random Effect	Fixed Effect	N	Random Effect	Fixed Effect
Current only	22	54% (49%, 59%)	54% (49%, 59%)	16	55% (49%, 60%)	55% (49%, 60%)
Prior only	22	21% (12%, 29%)	21% (12%, 29%)	16	22% (12%, 32%)	23% (13%, 31%)
Current and prior	22	47% (41%, 53%)	47% (43%, 51%)	16	47% (39%, 53%)	47% (42%, 51%)
Delta	22	-7% (-14%, 0%)	-7% (-14%, 0%)	16	-7% (-14%, 0%)	-7% (-14%, 0%)

Supplementary Table 17: Sensitivity of pooled estimates excluding studies at risk of bias, B/Victoria

	Original model			Sensitivity model		
	N	Random Effect	Fixed Effect	N	Random Effect	Fixed Effect
Current only	4	60% (35%, 75%)	60% (35%, 75%)	4	60% (35%, 75%)	60% (35%, 75%)
Prior only	4	17% (-15%, 40%)	17% (-15%, 40%)	4	17% (-15%, 40%)	17% (-15%, 40%)
Current and prior	4	50% (29%, 64%)	48% (30%, 62%)	4	50% (29%, 64%)	48% (30%, 62%)
Delta	4	-15% (-45%, 15%)	-15% (-45%, 15%)	4	-15% (-45%, 15%)	-15% (-45%, 15%)

Supplementary Table 18: Sensitivity of pooled estimates excluding studies at risk of bias, B/Yamagata

	Original model			Sensitivity model		
	N	Random Effect	Fixed Effect	N	Random Effect	Fixed Effect
Current only	8	56% (39%, 68%)	57% (46%, 65%)	5	64% (53%, 73%)	64% (53%, 73%)
Prior only	9	38% (25%, 49%)	38% (25%, 49%)	5	38% (20%, 51%)	38% (21%, 51%)
Current and prior	9	52% (42%, 60%)	52% (45%, 58%)	5	59% (51%, 66%)	59% (51%, 66%)
Delta	9	-5% (-17%, 6%)	-5% (-17%, 6%)	5	-7% (-19%, 5%)	-7% (-19%, 5%)

11.3 Restriction to Northern hemisphere studies

In the main analysis, estimates for the southern hemisphere were grouped with the preceding northern hemisphere season if the current and prior vaccine formulation was the same. Only one study was identified from the southern hemisphere, with relevant estimates for influenza A(H3N2) and B for 2017. It had the same formulation in the current and prior season and could be included with the 2016-17 estimates for the northern hemisphere. Nevertheless, it was removed and pooled estimates recalculated. Its removal had minimal impact on pooled estimates.

Supplementary Table 19: Sensitivity of pooled estimates for A(H3N2) when restricted to northern hemisphere studies

	Original model			Sensitivity model		
	N	Random Effect	Fixed Effect	N	Random Effect	Fixed Effect
Current only	30	37% (29%, 45%)	33% (28%, 39%)	29	37% (28%, 45%)	33% (27%, 38%)
Prior only	29	9% (-3%, 19%)	7% (0%, 14%)	28	9% (-4%, 20%)	8% (0%, 15%)
Current and prior	30	20% (12%, 27%)	18% (13%, 22%)	29	20% (12%, 27%)	18% (14%, 23%)
Delta	30	-18% (-26%, -11%)	-17% (-24%, -11%)	29	-18% (-25%, -10%)	-17% (-24%, -10%)

Supplementary Table 20: Sensitivity of pooled estimates for influenza B when restricted to northern hemisphere studies

	Original model			Sensitivity model		
	N	Random Effect	Fixed Effect	N	Random Effect	Fixed Effect
Current only	22	54% (49%, 59%)	54% (49%, 59%)	21	54% (49%, 59%)	54% (49%, 59%)
Prior only	22	21% (12%, 29%)	21% (12%, 29%)	21	21% (12%, 29%)	21% (12%, 29%)
Current and prior	22	47% (41%, 53%)	47% (43%, 51%)	21	46% (40%, 52%)	46% (42%, 50%)
Delta	22	-7% (-14%, 0%)	-7% (-14%, 0%)	21	-8% (-15%, -1%)	-8% (-15%, -1%)

11.4 Restriction to outpatient populations

Because concern exists that patients recruited through outpatient surveillance may differ systematically from patients identified through inpatient surveillance, pooled VE estimates were recalculated removing studies with inpatient populations or with a mixed inpatient/outpatient population. Restriction to outpatient studies, only, had minimal impact on pooled estimates.

Supplementary Table 21: Sensitivity of pooled estimates when restricted to outpatient studies, A(H1N1)pdm09

	Original model			Sensitivity model		
	N	Random Effect	Fixed Effect	N	Random Effect	Fixed Effect
Current only	19	58% (48%, 66%)	57% (50%, 63%)	14	59% (48%, 68%)	57% (50%, 63%)
Prior only	19	33% (21%, 43%)	33% (24%, 41%)	14	33% (22%, 43%)	33% (23%, 41%)
Current and prior	19	53% (44%, 60%)	51% (46%, 56%)	14	52% (43%, 60%)	50% (45%, 55%)
Delta	19	-9% (-16%, -1%)	-9% (-16%, -1%)	14	-10% (-18%, -2%)	-10% (-18%, -2%)

Supplementary Table 22: Sensitivity of pooled estimates when restricted to outpatient studies, A(H3N2)

	Original model			Sensitivity model		
	N	Random Effect	Fixed Effect	N	Random Effect	Fixed Effect
Current only	30	37% (29%, 45%)	33% (28%, 39%)	23	37% (27%, 45%)	33% (27%, 38%)
Prior only	29	9% (-3%, 19%)	7% (0%, 14%)	22	13% (1%, 23%)	11% (3%, 18%)
Current and prior	30	20% (12%, 27%)	18% (13%, 22%)	23	19% (10%, 26%)	17% (13%, 22%)
Delta	30	-18% (-26%, -11%)	-17% (-24%, -11%)	23	-18% (-27%, -9%)	-17% (-24%, -10%)

Supplementary Table 23: Sensitivity of pooled estimates when restricted to outpatient studies, influenza B

	Original model			Sensitivity model		
	N	Random Effect	Fixed Effect	N	Random Effect	Fixed Effect
Current only	22	54% (49%, 59%)	54% (49%, 59%)	17	55% (49%, 60%)	55% (49%, 60%)
Prior only	22	21% (12%, 29%)	21% (12%, 29%)	17	23% (13%, 32%)	23% (14%, 32%)
Current and prior	22	47% (41%, 53%)	47% (43%, 51%)	17	48% (41%, 55%)	48% (44%, 52%)
Delta	22	-7% (-14%, 0%)	-7% (-14%, 0%)	17	-6% (-13%, 1%)	-6% (-13%, 1%)

Supplementary Table 24: Sensitivity of pooled estimates when restricted to outpatient studies, B/Victoria

	Original model			Sensitivity model		
	N	Random Effect	Fixed Effect	N	Random Effect	Fixed Effect
Current only	4	60% (35%, 75%)	60% (35%, 75%)	4	60% (35%, 75%)	60% (35%, 75%)
Prior only	4	17% (-15%, 40%)	17% (-15%, 40%)	4	17% (-15%, 40%)	17% (-15%, 40%)
Current and prior	4	50% (29%, 64%)	48% (30%, 62%)	4	50% (29%, 64%)	48% (30%, 62%)
Delta	4	-15% (-45%, 15%)	-15% (-45%, 15%)	4	-15% (-45%, 15%)	-15% (-45%, 15%)

Supplementary Table 25: Sensitivity of pooled estimates when restricted to outpatient studies, B/Yamagata

	Original model			Sensitivity model		
	N	Random Effect	Fixed Effect	N	Random Effect	Fixed Effect
Current only	8	56% (39%, 68%)	57% (46%, 65%)	8	56% (39%, 68%)	57% (46%, 65%)
Prior only	9	38% (25%, 49%)	38% (25%, 49%)	8	38% (24%, 49%)	38% (24%, 49%)
Current and prior	9	52% (42%, 60%)	52% (45%, 58%)	8	53% (43%, 61%)	52% (45%, 59%)
Delta	9	-5% (-17%, 6%)	-5% (-17%, 6%)	8	-5% (-17%, 6%)	-5% (-17%, 6%)

11.5 Restriction to test-negative study designs only

The vast majority of studies contributing estimates to this review used the test-negative design, which incorporates certain features that put it at reduced risk of certain types of bias. Restriction of studies to include only those that used the test-negative design had minimal impact on pooled estimates, supporting the addition of observational studies which did not use this design.

Supplementary Table 26: Sensitivity of pooled estimates excluding studies that did not use the test-negative design, A(H1N1)pdm09

	Original model			Sensitivity model		
	N	Random Effect	Fixed Effect	N	Random Effect	Fixed Effect
Current only	19	58% (48%, 66%)	57% (50%, 63%)	18	58% (48%, 66%)	57% (50%, 63%)
Prior only	19	33% (21%, 43%)	33% (24%, 41%)	18	33% (21%, 43%)	33% (24%, 41%)
Current and prior	19	53% (44%, 60%)	51% (46%, 56%)	18	53% (43%, 60%)	51% (46%, 56%)
Delta	19	-9% (-16%, -1%)	-9% (-16%, -1%)	18	-9% (-16%, -1%)	-9% (-16%, -1%)

Supplementary Table 27: Sensitivity of pooled estimates excluding studies that did not use the test-negative design, A(H3N2)

	Original model			Sensitivity model		
	N	Random Effect	Fixed Effect	N	Random Effect	Fixed Effect
Current only	19	58% (48%, 66%)	57% (50%, 63%)	18	58% (48%, 66%)	57% (50%, 63%)
Prior only	19	33% (21%, 43%)	33% (24%, 41%)	18	33% (21%, 43%)	33% (24%, 41%)
Current and prior	19	53% (44%, 60%)	51% (46%, 56%)	18	53% (43%, 60%)	51% (46%, 56%)
Delta	19	-9% (-16%, -1%)	-9% (-16%, -1%)	18	-9% (-16%, -1%)	-9% (-16%, -1%)

Supplementary Table 28: Sensitivity of pooled estimates excluding studies that did not use the test-negative design, B/Yamagata

	Original model			Sensitivity model		
	N	Random Effect	Fixed Effect	N	Random Effect	Fixed Effect
Current only	19	58% (48%, 66%)	57% (50%, 63%)	18	58% (48%, 66%)	57% (50%, 63%)
Prior only	19	33% (21%, 43%)	33% (24%, 41%)	18	33% (21%, 43%)	33% (24%, 41%)
Current and prior	19	53% (44%, 60%)	51% (46%, 56%)	18	53% (43%, 60%)	51% (46%, 56%)
Delta	19	-9% (-16%, -1%)	-9% (-16%, -1%)	18	-9% (-16%, -1%)	-9% (-16%, -1%)

12 GRADE evaluation

The GRADE (Grading of Recommendations, Assessment, Development and Evaluations) system developed by the GRADE Working Group (Schünemann, 2013) was used to grade the certainty of the body of evidence presented by meta-analysis. Two reviewers (EJG, ER) collaboratively assessed quality of evidence using the GRADE methodology, synthesising evidence into summary of findings and GRADE tables. Classification of certainty was made according to GRADE criteria and represented as a final numerical score; Very low (1), Low (2), Moderate (3) or High (4).

Following GRADE recommendations by Cochrane, the studies included in this review started with a low-certainty rating because all were observational and at risk of confounding induced by the lack of randomization. Five domains were considered when assessing certainty (factors decreasing confidence): limitations in study design, inconsistency, indirectness, imprecision, and publication bias. Identification of problems in these domains reduced certainty and downgraded the rating. Ratings could be upgraded if studies included factors increasing confidence in the certainty of the evidence. These factors fell into 3 domains: large effects, dose response, and mitigated bias and confounding. For interpretation a GRADE assessment score of "Low certainty" can be interpreted as low confidence in the body of evidence for that outcome.

The policy question reviewed under the GRADE framework was: "What evidence exists on the effect of prior immunization on the efficacy and effectiveness of seasonal influenza vaccines, and does it warrant a change in policy that would result in improved public health outcomes?" This question was explored separately for each influenza virus type and subtype.

Supplementary Table 29: GRADE evaluation: What is the evidence on the vaccine effectiveness of repeated seasonal influenza vaccination against influenza A(H1N1)pdm09 across all ages?

			Rating	Adjustment of score
	No. studies / starting score	41 observational studies		2
Quality assessment	Factors decreasing confidence	Limitation in study design	Not serious[a]	0
		Inconsistency	Not serious[b]	0
		Indirectness	Not serious	0
		Imprecision	Not serious[c]	0
		Publication bias	Unlikely	0
	Factors increasing confidence	Large effect	No	0
		Dose-response	No	0
		Mitigated bias and confounding	No	0
	Final numerical score of quality of evidence			2
Conclusion		We have very low confidence in the evidence that VE against A(H1N1)pdm09 is attenuated by repeated influenza vaccination across all ages		

[a] Three studies of 13 included in A(H1N1)pdm09 all ages meta-analysis were judged at serious risk of bias using the ROBINS-I tool. However, sensitivity analyses showed that the impacts of these studies on overall VE estimates were very minimal.

[b] Heterogeneity was generally low within seasons and the moderate heterogeneity of pooled season estimates is plausibly explained by differences in study setting, age inclusions, country, and vaccine match/mismatch.

[c] A policy decision is unlikely be different if the true effects were at either the lower or upper ends of the confidence limits of current+prior or current only pooled estimates.

Supplementary Table 30: GRADE evaluation: What is the evidence on the vaccine effectiveness of repeated seasonal influenza vaccination against influenza A(H3N2) across all ages?

			Rating	Adjustment of score
	No. studies / starting score	41 observational studies		2
Quality assessment	Factors decreasing confidence	Limitation in study design	Not serious[a]	0
		Inconsistency	Not serious[b]	0
		Indirectness	Not serious	0
		Imprecision	Serious[c]	-1
		Publication bias	Unlikely	0
	Factors increasing confidence	Large effect	No	0
		Dose-response	No	0
		Mitigated bias and confounding	No	0
	Final numerical score of quality of evidence			1
Conclusion		We have very low confidence in the evidence that VE against A(H3N2) is attenuated by repeated influenza vaccination across all ages		

[a] Four studies of 18 included in A(H3N2) all ages meta-analysis were judged at serious risk of bias using the ROBINS-I tool. However, sensitivity analyses showed that the impacts of these studies on overall VE estimates were minimal.

[b] Heterogeneity was generally low within seasons and the moderate heterogeneity of pooled season estimates is plausibly explained by differences in study setting, age inclusions, country, and vaccine match/mismatch.

[c] While current+prior and current only pooled season estimates do not indicate a policy decision is likely to be different if the true effects were at either the lower or upper ends of their confidence limits, the 2014-2015 current+prior estimate -5 (95% CI: -19, 7) crosses the null value and provides evidence that a policy decision may be made differently if the true effects were at either the lower or upper ends of their confidence limits.

Supplementary Table 31: GRADE evaluation: What is the evidence on the vaccine effectiveness of repeated seasonal influenza vaccination against influenza B across all ages?

			Rating	Adjustment of score
	No. studies / starting score	41 observational studies		2
Quality assessment	Factors decreasing confidence	Limitation in study design	Not serious[a]	0
		Inconsistency	Not serious[b]	0
		Indirectness	Not serious	0
		Imprecision	Serious[c]	2
		Publication bias	Unlikely	0
	Factors increasing confidence	Large effect	No	0
		Dose-response	No	0
		Mitigated bias and confounding	No	0
	Final numerical score of quality of evidence			2
Conclusion		We have very low confidence in the evidence that VE against influenza B is attenuated by repeated influenza vaccination across all ages		

[a] Three studies of 13 included in influenza B all ages meta-analysis were judged at serious risk of bias using the ROBINS-I tool. However, sensitivity analyses showed that the impacts of these studies on overall VE estimates were very minimal.

[b] Overall, heterogeneity was generally low within seasons, low to moderate for pooled seasons and can be plausibly explained by differences in study setting, age inclusions, country, and vaccine match/mismatch. Large I2 values for current and prior estimates in the 2014-2015 and 2017-2018 seasons indicate high heterogeneity however this can be plausibly explained by previously listed factors in addition to limited numbers of study estimates.

[c] A policy decision is unlikely be different if the true effects were at either the lower or upper ends of the confidence limits of current+prior or current only pooled estimates.

Supplementary Table 32: GRADE evaluation: What is the evidence on the vaccine effectiveness of repeated seasonal influenza vaccination against influenza B/Victoria across all ages?

			Rating	Adjustment of score
	No. studies / starting score	41 observational studies		2
Quality assessment	Factors decreasing confidence	Limitation in study design	Not serious[a]	0
		Inconsistency	Not serious[b]	0
		Indirectness	Not serious	0
		Imprecision	Serious[c]	-1
		Publication bias	Unlikely[b]	0
	Factors increasing confidence	Large effect	No	0
		Dose-response	No	0
		Mitigated bias and confounding	No	0
	Final numerical score of quality of evidence			1
Conclusion		We have very low confidence in the evidence that VE against influenza B/Yamagata is attenuated by repeated influenza vaccination across all ages		

[a] No studies of four included in B/Victoria all ages meta-analysis were judged at serious risk of bias using the ROBINS-I tool.

[b] Heterogeneity was low within season specific and pooled season estimates, any heterogeneity is plausibly explained by differences in study setting, age inclusions, country, and vaccine match/mismatch.

[c] Very few estimates available for each season and many seasons unrepresented.

[d] There is an insufficient number of study estimates for B/Victoria to make a judgement on publication bias.

Supplementary Table 33: GRADE evaluation: What is the evidence on the vaccine effectiveness of repeated seasonal influenza vaccination against influenza B/Yamagata across all ages?

			Rating	Adjustment of score
	No. studies / starting score	41 observational studies		2
Quality assessment	Factors decreasing confidence	Limitation in study design	Not serious[a]	0
		Inconsistency	Not serious[b]	0
		Indirectness	Not serious	0
		Imprecision	Serious[c]	-1
		Publication bias	Unlikely[d]	0
	Factors increasing confidence	Large effect	No	0
		Dose-response	No	0
		Mitigated bias and confounding	No	0
	Final numerical score of quality of evidence			1
Conclusion		We have very low confidence in the evidence that VE against influenza B/Yamagata is attenuated by repeated influenza vaccination across all ages		

[a] No studies of four included in B/Yamagata all ages meta-analysis were judged at serious risk of bias using the ROBINS-I tool.

[b] Heterogeneity was low within season specific and pooled season estimates, any heterogeneity is plausibly explained by differences in study setting, age inclusions, country, and vaccine match/mismatch.

[c] Very few estimates available for each season and many seasons unrepresented.

[d] There is an insufficient number of study estimates for B/Victoria to make a judgement on publication bias.

Supplementary Table 34: GRADE evaluation: summary of findings

Current season only vaccination compared with current+prior season vaccination against seasonal influenza						
Patients or population: anyone eligible for seasonal influenza vaccination<br>Settings: Northern and Southern hemisphere, inpatient and outpatient, all ages<br>Intervention: vaccination in the current season but not in the season immediately prior (current only)<br>Comparison: annual influenza vaccination (current+prior)						
	Pooled season VE (95%CI)			Number of	Certainty of the	
Outcome	current+prior	current only	ΔVE (95% CI)	participants (studies)	evidence (GRADE)	Comments
A(H1N1)pdm09	53 (44, 60)	58 (48, 66)	-9 (-16, -1)	27759 (13)	2, low[a]	
A(H3N2)	20 (12, 27)	37 (29, 45)	-18 (-26, -11)	55135 (18)	1, very low[b]	
Influenza B	47 (41, 53)	54 (49, 59)	-7 (-14, 0)	37736 (13)	2, low[c]	
B/Victoria	50 (29, 64)	60 (35, 75)	-15 (-45, 15)	4634 (4)	1, very low[d]	
B/Yamagata	52 (42, 60)	56 (39, 68)	-5 (-17, 6)	16096 (7)	1, very low[e]	

[a] We have low confidence in the evidence that VE against A(H1N1)pdm09 is attenuated by repeated influenza vaccination in all ages.

[b] We have very low confidence in the evidence that VE against A(H3N2) is attenuated by repeated influenza vaccination in all ages. While current+prior and current only pooled season estimates do not indicate a policy decision is likely to be different if the true effects were at either the lower or upper ends of their confidence limits, the 2014-2015 current+prior estimate -5 (95% CI: -19, 7) crosses the null value and provides evidence that a policy decision may be made differently if the true effects were at either the lower or upper ends of their confidence limits.

[c] We have low confidence in the evidence that VE against Influenza B of any lineage is attenuated by repeated influenza vaccination in all ages.

[d] We have very low confidence in the evidence that VE against B/Victoria is attenuated by repeated influenza vaccination in all ages. Very few estimates available for each season and many seasons unrepresented. There is an insufficient number of study estimates for B/Victoria to make a judgement on publication bias.

[e] We have very low confidence in the evidence that VE against B/Yamagata is attenuated by repeated influenza vaccination in all ages. Very few estimates available for each season and many seasons unrepresented. There is an insufficient number of study estimates for B/Yamagata to make a judgement on publication bias.

13 References

References

[1] N. L. Boddington, F. Warburton, H. Zhao, N. Andrews, J. Ellis, M. Donati, and R. G. Pebody. Influenza vaccine effectiveness against hospitalisation due to laboratory-confirmed influenza in children in england in the 2015-2016 influenza season - a test-negative case-control study. *Epidemiol Infect*, 147:e201, 2019.

[2] S. A. Buchan, H. Chung, M. A. Campitelli, N. S. Crowcroft, J. B. Gubbay, T. Karnauchow, K. Katz, A. J. McGeer, J. D. McNally, D. Richardson, S. E. Richardson, L. C. Rosella, A. Simor, M. Smieja, D. Tran, G. Zahariadis, and J. C. Kwong. Vaccine effectiveness against laboratory-confirmed influenza hospitalizations among young children during the 2010-11 to 2013-14 influenza seasons in ontario, canada. *PLoS One*, 12(11):e0187834, 2017.

[3] S. A. Buchan, S. Booth, A. N. Scott, K. A. Simmonds, L. W. Svenson, S. J. Drews, M. L. Russell, N. S. Crowcroft, M. Loeb, B. F. Warshawsky, and J. C. Kwong. Effectiveness of live attenuated vs inactivated influenza vaccines in children during the 2012-2013 through 2015-2016 influenza seasons in alberta, canada: A canadian immunization research network (cirn) study. *JAMA Pediatr*, 172(9):e181514, 2018.

[4] I. Casado, A. Dominguez, D. Toledo, J. Chamorro, L. Force, N. Soldevila, J. Astray, M. Egurrola, P. Godoy, J. M. Mayoral, S. Tamames, F. Sanz, J. Castilla, and Group Project Pi12/ Working. Effect of influenza vaccination on the prognosis of hospitalized influenza patients. *Expert Rev Vaccines*, 15(3):425–32, 2016.

[5] I. Casado, A. Dominguez, D. Toledo, J. Chamorro, J. Astray, M. Egurrola, M. A. Fernandez-Sierra, V. Martin, M. Morales-Suarez-Varela, P. Godoy, J. Castilla, and P. I. Working Group Project. Repeated influenza vaccination for preventing severe and fatal influenza infection in older adults: a multicentre case-control study. *CMAJ*, 190(1):E3–E12, 2018.

[6] J. Castilla, J. Moran, V. Martinez-Artola, G. Reina, I. Martinez-Baz, M. Garcia Cenoz, N. Alvarez, F. Irisarri, M. Arriazu, F. Elia, and E. Salcedo. Effectiveness of trivalent seasonal and monovalent influenza a(h1n1)2009 vaccines in population with major chronic conditions of navarre, spain: 2010/11 mid-season analysis. *Euro Surveill*, 16(7), 2011.

[7] J. Castilla, A. Navascues, M. Fernandez-Alonso, G. Reina, F. Pozo, I. Casado, M. Guevara, I. Martinez-Baz, A. Barricarte, C. Ezpeleta, Network Primary Health Care Sentinel, and Navarra Network for Influenza Surveillance in Hospitals of. Effectiveness of subunit influenza vaccination in the 2014-2015 season and residual effect of split vaccination in previous seasons. *Vaccine*, 34(11):1350–7, 2016.

[8] J. Castilla, A. Navascues, I. Casado, J. Diaz-Gonzalez, A. Perez-Garcia, L. Fernandino, I. Martinez-Baz, A. Aguinaga, F. Pozo, C. Ezpeleta, Network Primary Health Care Sentinel, and Navarre The Network For Influenza Surveillance In Hospitals Of. Combined effectiveness of prior and current season influenza vaccination in northern spain: 2016/17 mid-season analysis. *Euro Surveill*, 22(7), 2017.

[9] J. Castilla, A. Navascues, I. Casado, A. Perez-Garcia, A. Aguinaga, G. Ezpeleta, F. Pozo, C. Ezpeleta, I. Martinez-Baz, Network Primary Health Care Sentinel, and Navarre Network For Influenza Surveillance In Hospitals Of. Interim effectiveness of trivalent influenza

vaccine in a season dominated by lineage mismatched influenza b, northern spain, 2017/18. *Euro Surveill*, 23(7):18–00057, 2018.

[10] J. Castilla, M. E. Portillo, I. Casado, F. Pozo, A. Navascues, M. Adelantado, C. Gomez Ibanez, C. Ezpeleta, I. Martinez-Baz, Network Primary Health Care Sentinel, and Navarre Network for Influenza Surveillance in Hospitals of. Effectiveness of the current and prior influenza vaccinations in northern spain, 2018-2019. *Vaccine*, 38(8):1925–1932, 2020.

[11] A. C. Cheng, K. K. Macartney, G. W. Waterer, T. Kotsimbos, P. M. Kelly, C. C. Blyth, and Investigators Influenza Complications Alert Network. Repeated vaccination does not appear to impact upon influenza vaccine effectiveness against hospitalization with confirmed influenza. *Clin Infect Dis*, 64(11):1564–1572, 2017.

[12] A. Dominguez, N. Soldevila, D. Toledo, P. Godoy, E. Espejo, M. A. Fernandez, J. M. Mayoral, J. Castilla, M. Egurrola, S. Tamames, J. Astray, M. Morales-Suarez-Varela, and Pi The Working Group Of The Project. The effectiveness of influenza vaccination in preventing hospitalisations of elderly individuals in two influenza seasons: a multicentre case-control study, spain, 2013/14 and 2014/15. *Euro Surveill*, 22(34), 2017.

[13] N. El Omeiri, E. Azziz-Baumgartner, M. G. Thompson, R. EVELAC-i network participants, W. Clara, M. Cerpa, R. Palekar, S. Mirza, and A. M. Ropero-Alvarez. Seasonal influenza vaccine effectiveness against laboratory-confirmed influenza hospitalizations - latin america, 2013. *Vaccine*, 36(24):3555–3566, 2018.

[14] J. M. Ferdinands, A. M. Fry, S. Reynolds, J. Petrie, B. Flannery, M. L. Jackson, and E. A. Belongia. Intraseason waning of influenza vaccine protection: Evidence from the us influenza vaccine effectiveness network, 2011-12 through 2014-15. *Clin Infect Dis*, 64(5):544–550, 2017.

[15] B. Flannery, J. R. Chung, A. S. Monto, E. T. Martin, E. A. Belongia, H. Q. McLean, M. Gaglani, K. Murthy, R. K. Zimmerman, M. P. Nowalk, M. L. Jackson, L. A. Jackson, M. A. Rolfes, S. Spencer, A. M. Fry, and U. S. Flu VE Investigators. Influenza vaccine effectiveness in the united states during the 2016-2017 season. *Clin Infect Dis*, 68(11):1798–1806, 2019.

[16] C. Fu, J. Xu, J. Lin, M. Wang, K. Li, J. Ge, and M. G. Thompson. Concurrent and cross-season protection of inactivated influenza vaccine against a(h1n1)pdm09 illness among young children: 2012-2013 case-control evaluation of influenza vaccine effectiveness. *Vaccine*, 33(25):2917–21, 2015.

[17] M. Gaglani, J. Pruszynski, K. Murthy, L. Clipper, A. Robertson, M. Reis, J. R. Chung, P. A. Piedra, V. Avadhanula, M. P. Nowalk, R. K. Zimmerman, M. L. Jackson, L. A. Jackson, J. G. Petrie, S. E. Ohmit, A. S. Monto, H. Q. McLean, E. A. Belongia, A. M. Fry, and B. Flannery. Influenza vaccine effectiveness against 2009 pandemic influenza a(h1n1) virus differed by vaccine type during 2013-2014 in the united states. *J Infect Dis*, 213(10):1546–56, 2016.

[18] A. Gherasim, I. Martinez-Baz, J. Castilla, F. Pozo, A. Larrauri, and E. V. A. working group cyc. Effect of previous and current vaccination against influenza a(h1n1)pdm09, a(h3n2), and b during the post-pandemic period 2010-2016 in spain. *PLoS One*, 12(6):e0179160, 2017.

[19] C. G. Grijalva, L. R. Feldstein, H. K. Talbot, M. Aboodi, A. H. Baughman, S. M. Brown, J. D. Casey, H. L. Erickson, M. C. Exline, D. C. Files, K. W. Gibbs, A. A. Ginde, M. N. Gong, N. Halasa, A. Khan, C. J. Lindsell, S. K. Nwosu, I. D. Peltan, M. E. Prekker, T. W. Rice, N. I. Shapiro, J. S. Steingrub, W. B. Stubblefield, M. W. Tenforde, M. M. Patel, W. H. Self, Influenza, and Network Other Viruses in the Acutely Ill. Influenza vaccine effectiveness for prevention of severe influenza-associated illness among adults in the united states, 2019-2020: A test-negative study. *Clin Infect Dis*, 73(8):1459–1468, 2021.

[20] M. L. Jackson, J. R. Chung, L. A. Jackson, C. H. Phillips, J. Benoit, A. S. Monto, E. T. Martin, E. A. Belongia, H. Q. McLean, M. Gaglani, K. Murthy, R. Zimmerman, M. P. Nowalk, A. M. Fry, and B. Flannery. Influenza vaccine effectiveness in the united states during the 2015-2016 season. *N Engl J Med*, 377(6):534–543, 2017.

[21] S. Jimenez-Jorge, C. Savulescu, F. Pozo, S. de Mateo, I. Casas, J. Ledesma, A. Larrauri, E. V. A. Study Team cyc, and System Spanish Influenza Sentinel Surveillance. Effectiveness of the 2010-11 seasonal trivalent influenza vaccine in spain: cyceva study. *Vaccine*, 30(24):3595–602, 2012.

[22] S. S. Kim, B. Flannery, I. M. Foppa, J. R. Chung, M. P. Nowalk, R. K. Zimmerman, M. Gaglani, A. S. Monto, E. T. Martin, E. A. Belongia, H. Q. McLean, M. L. Jackson, L. A. Jackson, and M. Patel. Effects of prior season vaccination on current season vaccine effectiveness in the united states flu vaccine effectiveness network, 2012-2013 through 2017-2018. *Clin Infect Dis*, 73(3):497–505, 2021.

[23] E. Kissling, M. Valenciano, F. Pozo, A. M. Vilcu, A. Reuss, C. Rizzo, A. Larrauri, J. K. Horvath, M. Brytting, L. Domegan, M. Korczynska, A. Meijer, A. Machado, A. Ivanciuc, V. Visekruna Vucina, S. van der Werf, B. Schweiger, A. Bella, A. Gherasim, A. Ferenczi, K. Zakikhany, O. Donnell J, I. Paradowska-Stankiewicz, F. Dijkstra, R. Guiomar, M. Lazar, S. Kurecic Filipovic, K. Johansen, A. Moren, and I. Move I. Move study team. 2015/16 i-move/i-move+ multicentre case-control study in europe: Moderate vaccine effectiveness estimates against influenza a(h1n1)pdm09 and low estimates against lineage-mismatched influenza b among children. *Influenza Other Respir Viruses*, 12(4):423–437, 2018.

[24] E. Kissling, F. Pozo, S. Buda, A. M. Vilcu, A. Gherasim, M. Brytting, L. Domegan, V. Gomez, A. Meijer, M. Lazar, V. V. Vucina, R. Durrwald, S. van der Werf, A. Larrauri, T. Enkirch, J. O'Donnell, R. Guiomar, M. Hooiveld, G. Petrovic, E. Stoian, P. Penttinen, M. Valenciano, and I. Move Primary Care Study Team. Low 2018/19 vaccine effectiveness against influenza a(h3n2) among 15-64-year-olds in europe: exploration by birth cohort. *Euro Surveill*, 24(48):1900604, 2019.

[25] E. Kissling, F. Pozo, S. Buda, A. M. Vilcu, C. Rizzo, A. Gherasim, J. K. Horvath, M. Brytting, L. Domegan, A. Meijer, I. Paradowska-Stankiewicz, A. Machado, V. V. Vucina, M. Lazar, K. Johansen, R. Durrwald, S. van der Werf, A. Bella, A. Larrauri, A. Ferenczi, K. Zakikhany, J. O'Donnell, F. Dijkstra, J. Bogusz, R. Guiomar, S. K. Filipovic, D. Pitigoi, P. Penttinen, M. Valenciano, and I. Move I. Move study team. Effectiveness of influenza vaccine against influenza a in europe in seasons of different a(h1n1)pdm09 and the same a(h3n2) vaccine components (2016-17 and 2017-18). *Vaccine X*, 3:100042, 2019.

[26] J. C. Kwong, H. Chung, J. K. Jung, S. A. Buchan, A. Campigotto, M. A. Campitelli, N. S. Crowcroft, J. B. Gubbay, T. Karnauchow, K. Katz, A. J. McGeer, J. D. McNally, D. C. Richardson, S. E. Richardson, L. C. Rosella, K. L. Schwartz, A. Simor, M. Smieja,

G. Zahariadis, and Investigators On Behalf Of The Canadian Immunization Research Network Cirn. The impact of repeated vaccination using 10-year vaccination history on protection against influenza in older adults: a test-negative design study across the 2010/11 to 2015/16 influenza seasons in ontario, canada. *Euro Surveill*, 25(1):1900245, 2020.

[27] C. Ma, Y. Pan, L. Zhang, Y. Zhang, S. Wu, Y. Sun, W. Duan, M. Zhang, Q. Wang, and P. Yang. Influenza vaccine effectiveness against medically attended influenza illness in beijing, china, 2014/15 season. *Hum Vaccin Immunother*, 13(10):2379–2384, 2017.

[28] I. Martinez-Baz, V. Martinez-Artola, G. Reina, M. Guevara, M. G. Cenoz, J. Moran, F. Irisarri, M. Arriazu, E. Albeniz, J. Castilla, and Navarre Primary Health Care Sentinel Network of. Effectiveness of the trivalent influenza vaccine in navarre, spain, 2010-2011: a population-based test-negative case-control study. *BMC Public Health*, 13:191, 2013.

[29] I. Martinez-Baz, I. Casado, A. Navascues, J. Diaz-Gonzalez, A. Aguinaga, L. Barrado, J. Delfrade, C. Ezpeleta, and J. Castilla. Effect of repeated vaccination with the same vaccine component against 2009 pandemic influenza a(h1n1) virus. *J Infect Dis*, 215(6):847–855, 2017.

[30] I. Martinez-Baz, A. Navascues, I. Casado, A. Aguinaga, C. Ezpeleta, and J. Castilla. Simple models to include influenza vaccination history when evaluating the effect of influenza vaccination. *Euro Surveill*, 26(32):2001099, 2021.

[31] I. Martinez-Baz, A. Navascues, M. E. Portillo, I. Casado, U. Fresan, C. Ezpeleta, and J. Castilla. Effect of influenza vaccination in preventing laboratory-confirmed influenza hospitalization in patients with diabetes mellitus. *Clin Infect Dis*, 73(1):107–114, 2021.

[32] H. Q. McLean, M. G. Thompson, M. E. Sundaram, J. K. Meece, D. L. McClure, T. C. Friedrich, and E. A. Belongia. Impact of repeated vaccination on vaccine effectiveness against influenza a(h3n2) and b during 8 seasons. *Clin Infect Dis*, 59(10):1375–85, 2014.

[33] H. Q. McLean, M. G. Thompson, M. E. Sundaram, B. A. Kieke, M. Gaglani, K. Murthy, P. A. Piedra, R. K. Zimmerman, M. P. Nowalk, J. M. Raviotta, M. L. Jackson, L. Jackson, S. E. Ohmit, J. G. Petrie, A. S. Monto, J. K. Meece, S. N. Thaker, J. R. Clippard, S. M. Spencer, A. M. Fry, and E. A. Belongia. Influenza vaccine effectiveness in the united states during 2012-2013: variable protection by age and virus type. *J Infect Dis*, 211(10):1529–40, 2015.

[34] H. Q. McLean, H. Caspard, M. R. Griffin, K. A. Poehling, M. Gaglani, E. A. Belongia, H. K. Talbot, T. R. Peters, K. Murthy, and C. S. Ambrose. Effectiveness of live attenuated influenza vaccine and inactivated influenza vaccine in children during the 2014-2015 season. *Vaccine*, 35(20):2685–2693, 2017.

[35] H. Q. McLean, H. Caspard, M. R. Griffin, M. Gaglani, T. R. Peters, K. A. Poehling, C. S. Ambrose, and E. A. Belongia. Association of prior vaccination with influenza vaccine effectiveness in children receiving live attenuated or inactivated vaccine. *JAMA Netw Open*, 1(6):e183742, 2018.

[36] A. Mira-Iglesias, F. X. Lopez-Labrador, B. Guglieri-Lopez, M. Tortajada-Girbes, V. Baselga-Moreno, L. Cano, J. Mollar-Maseres, M. Carballido-Fernandez, G. Schwarz-Chavarri, J. Diez-Domingo, J. Puig-Barbera, Influenza Valencia Hospital Network For The Study Of, and Disease Respiratory Viruses. Influenza vaccine effectiveness in preventing hospitalisation of individuals 60 years of age and over with laboratory-confirmed

influenza, valencia region, spain, influenza season 2016/17. *Euro Surveill*, 23(8):17–00318, 2018.

[37] A. Mira-Iglesias, F. X. Lopez-Labrador, V. Baselga-Moreno, M. Tortajada-Girbes, J. Mollar-Maseres, M. Carballido-Fernandez, G. Schwarz-Chavarri, J. Puig-Barbera, J. Diez-Domingo, Influenza On Behalf Of The Valencia Hospital Network For The Study Of, and Disease Respiratory Viruses. Influenza vaccine effectiveness against laboratory-confirmed influenza in hospitalised adults aged 60 years or older, valencia region, spain, 2017/18 influenza season. *Euro Surveill*, 24(31):1800461, 2019.

[38] M. K. Nichols, M. K. Andrew, L. Ye, T. F. Hatchette, A. Ambrose, G. Boivin, W. Bowie, G. Dos Santos, M. Elsherif, K. Green, F. Haguinet, K. Katz, J. Leblanc, M. Loeb, D. MacKinnon-Cameron, A. McCarthy, J. E. McElhaney, A. McGeer, J. Powis, D. Richardson, M. Semret, R. Sharma, V. Shinde, D. Smyth, S. Trottier, L. Valiquette, D. Webster, S. A. McNeil, and Network Serious Outcomes Surveillance Network of the Canadian Immunization Research. The impact of prior season vaccination on subsequent influenza vaccine effectiveness to prevent influenza-related hospitalizations over 4 influenza seasons in canada. *Clin Infect Dis*, 69(6):970–979, 2019.

[39] S. E. Ohmit, J. G. Petrie, R. E. Malosh, B. J. Cowling, M. G. Thompson, D. K. Shay, and A. S. Monto. Influenza vaccine effectiveness in the community and the household. *Clin Infect Dis*, 56(10):1363–9, 2013.

[40] S. E. Ohmit, J. G. Petrie, R. E. Malosh, A. M. Fry, M. G. Thompson, and A. S. Monto. Influenza vaccine effectiveness in households with children during the 2012-2013 season: assessments of prior vaccination and serologic susceptibility. *J Infect Dis*, 211(10):1519–28, 2015.

[41] S. E. Ohmit, J. G. Petrie, R. E. Malosh, E. Johnson, R. Truscon, B. Aaron, C. Martens, C. Cheng, A. M. Fry, and A. S. Monto. Substantial influenza vaccine effectiveness in households with children during the 2013-2014 influenza season, when 2009 pandemic influenza a(h1n1) virus predominated. *J Infect Dis*, 213(8):1229–36, 2016.

[42] A. Ortqvist, M. Brytting, A. Leval, and M. P. Hergens. Impact of repeated influenza vaccinations in persons over 65years of age: A large population-based cohort study of severe influenza over six consecutive seasons, 2011/12-2016/17. *Vaccine*, 36(37):5556–5564, 2018.

[43] R. G. Pebody, N. Andrews, D. M. Fleming, J. McMenamin, S. Cottrell, B. Smyth, H. Durnall, C. Robertson, W. Carman, J. Ellis, P. Sebastian-Pillai, M. Zambon, C. Kearns, C. Moore, D. R. Thomas, and J. M. Watson. Age-specific vaccine effectiveness of seasonal 2010/2011 and pandemic influenza a(h1n1) 2009 vaccines in preventing influenza in the united kingdom. *Epidemiol Infect*, 141(3):620–30, 2013.

[44] R. Pebody, F. Warburton, J. Ellis, N. Andrews, A. Potts, S. Cottrell, A. Reynolds, R. Gunson, C. Thompson, M. Galiano, C. Robertson, N. Gallagher, M. Sinnathamby, I. Yonova, A. Correa, C. Moore, M. Sartaj, S. de Lusignan, J. McMenamin, and M. Zambon. End-of-season influenza vaccine effectiveness in adults and children, united kingdom, 2016/17. *Euro Surveill*, 22(44):17–00306, 2017.

[45] R. Pebody, A. Djennad, J. Ellis, N. Andrews, D. F. P. Marques, S. Cottrell, A. J. Reynolds, R. Gunson, M. Galiano, K. Hoschler, A. Lackenby, C. Robertson, M. O'Doherty, M. Sinnathamby, N. Panagiotopoulos, I. Yonova, R. Webb, C. Moore, M. Donati, M. Sartaj, S. J.

Shepherd, J. McMenamin, S. de Lusignan, and M. Zambon. End of season influenza vaccine effectiveness in adults and children in the united kingdom in 2017/18. *Euro Surveill*, 24(31):1800488, 2019.

[46] R. Pebody, H. Whitaker, H. Zhao, N. Andrews, J. Ellis, M. Donati, and M. Zambon. Protection provided by influenza vaccine against influenza-related hospitalisation in >/=65 year olds: Early experience of introduction of a newly licensed adjuvanted vaccine in england in 2018/19. *Vaccine*, 38(2):173–179, 2020.

[47] R. G. Pebody, H. Whitaker, J. Ellis, N. Andrews, D. F. P. Marques, S. Cottrell, A. J. Reynolds, R. Gunson, C. Thompson, M. Galiano, A. Lackenby, C. Robertson, M. G. O'Doherty, K. Owens, I. Yonova, S. J. Shepherd, C. Moore, J. Johnston, M. Donati, J. McMenamin, S. Lusignan, and M. Zambon. End of season influenza vaccine effectiveness in primary care in adults and children in the united kingdom in 2018/19. *Vaccine*, 38(3):489–497, 2020.

[48] R. G. Pebody, H. Zhao, H. J. Whitaker, J. Ellis, M. Donati, M. Zambon, and N. Andrews. Effectiveness of influenza vaccine in children in preventing influenza associated hospitalisation, 2018/19, england. *Vaccine*, 38(2):158–164, 2020.

[49] J. G. Petrie, S. E. Ohmit, C. K. Cheng, E. T. Martin, R. E. Malosh, A. S. Lauring, L. E. Lamerato, K. C. Reyes, B. Flannery, J. M. Ferdinands, and A. S. Monto. Influenza vaccine effectiveness against antigenically drifted influenza higher than expected in hospitalized adults: 2014-2015. *Clin Infect Dis*, 63(8):1017–25, 2016.

[50] J. G. Petrie, R. E. Malosh, C. K. Cheng, S. E. Ohmit, E. T. Martin, E. Johnson, R. Truscon, M. C. Eichelberger, L. V. Gubareva, A. M. Fry, and A. S. Monto. The household influenza vaccine effectiveness study: Lack of antibody response and protection following receipt of 2014-2015 influenza vaccine. *Clin Infect Dis*, 65(10):1644–1651, 2017.

[51] L. N. Powell and R. E. Begue. Influenza vaccine effectiveness among children for the 2017-2018 season. *J Pediatric Infect Dis Soc*, 9(4):468–473, 2020.

[52] S. Rao, A. Moss, M. M. Lamb, and E. J. Asturias. Evaluation of influenza vaccine effectiveness among young children receiving consecutive versus nonconsecutive vaccination during influenza a(h3n2)-predominant seasons. *J Pediatric Infect Dis Soc*, 10(3):359–362, 2021.

[53] M. Rondy, O. Launay, J. Puig-Barbera, G. Gefenaite, J. Castilla, K. de Gaetano Donati, F. Galtier, E. Hak, M. Guevara, S. Costanzo, I. V. E. network European hospital, and A. Moren. 2012/13 influenza vaccine effectiveness against hospitalised influenza a(h1n1)pdm09, a(h3n2) and b: estimates from a european network of hospitals. *Euro Surveill*, 20(2), 2015.

[54] M. Rondy, A. Gherasim, I. Casado, O. Launay, C. Rizzo, D. Pitigoi, A. Mickiene, S. D. Marbus, A. Machado, R. K. Syrjanen, I. Pem-Novose, J. K. Horvath, A. Larrauri, J. Castilla, P. Vanhems, V. Alfonsi, A. E. Ivanciuc, M. Kuliese, R. van Gageldonk-Lafeber, V. Gomez, N. Ikonen, Z. Lovric, A. Ferenczi, A. Moren, and I. Move hospital working group. Low 2016/17 season vaccine effectiveness against hospitalised influenza a(h3n2) among elderly: awareness warranted for 2017/18 season. *Euro Surveill*, 22(41):17–00645, 2017.

[55] M. Rondy, O. Launay, J. Castilla, S. Costanzo, J. Puig-Barbera, G. Gefenaite, A. Larrauri, C. Rizzo, D. Pitigoi, R. K. Syrjanen, A. Machado, S. Kurecic Filipovic, J. Krisztina Horvath, I. Paradowska-Stankiewicz, S. Marbus, Nhove I. Move working group In, and A. Moren. Repeated seasonal influenza vaccination among elderly in europe: Effects on laboratory confirmed hospitalised influenza. *Vaccine*, 35(34):4298–4306, 2017.

[56] A. M. C. Rose, E. Kissling, A. Gherasim, I. Casado, A. Bella, O. Launay, M. Lazar, S. Marbus, M. Kuliese, R. Syrjanen, A. Machado, S. Kurecic Filipovic, A. Larrauri, J. Castilla, V. Alfonsi, F. Galtier, A. Ivanciuc, A. Meijer, A. Mickiene, N. Ikonen, V. Gomez, Z. Lovric Makaric, A. Moren, M. Valenciano, and I. Move Hospital study team. Vaccine effectiveness against influenza a(h3n2) and b among laboratory-confirmed, hospitalised older adults, europe, 2017-18: A season of b lineage mismatched to the trivalent vaccine. *Influenza Other Respir Viruses*, 14(3):302–310, 2020.

[57] N. Saito, K. Komori, M. Suzuki, K. Morimoto, T. Kishikawa, T. Yasaka, and K. Ariyoshi. Negative impact of prior influenza vaccination on current influenza vaccination among people infected and not infected in prior season: A test-negative case-control study in japan. *Vaccine*, 35(4):687–693, 2017.

[58] N. Saito, K. Komori, M. Suzuki, T. Kishikawa, T. Yasaka, and K. Ariyoshi. Dose-dependent negative effects of prior multiple vaccinations against influenza a and influenza b among schoolchildren: A study of kamigoto island in japan during the 2011-2012, 2012-2013, and 2013-2014 influenza seasons. *Clin Infect Dis*, 67(6):897–904, 2018.

[59] M. Shinjoh, N. Sugaya, Y. Yamaguchi, N. Iibuchi, I. Kamimaki, A. Goto, H. Kobayashi, Y. Kobayashi, M. Shibata, S. Tamaoka, Y. Nakata, A. Narabayashi, M. Nishida, Y. Hirano, T. Munenaga, K. Morita, K. Mitamura, T. Takahashi, and Group Keio Pediatric Influenza Research. Inactivated influenza vaccine effectiveness and an analysis of repeated vaccination for children during the 2016/17 season. *Vaccine*, 36(37):5510–5518, 2018.

[60] C. R. Simpson, N. I. Lone, K. Kavanagh, L. D. Ritchie, C. Robertson, A. Sheikh, and J. McMenamin. Trivalent inactivated seasonal influenza vaccine effectiveness for the prevention of laboratory-confirmed influenza in a scottish population 2000 to 2009. *Euro Surveill*, 20(8), 2015.

[61] D. M. Skowronski, N. Z. Janjua, G. De Serres, A. L. Winter, J. A. Dickinson, J. L. Gardy, J. Gubbay, K. Fonseca, H. Charest, N. S. Crowcroft, M. D. Fradet, N. Bastien, Y. Li, M. Krajden, S. Sabaiduc, and M. Petric. A sentinel platform to evaluate influenza vaccine effectiveness and new variant circulation, canada 2010-2011 season. *Clin Infect Dis*, 55(3):332–42, 2012.

[62] D. M. Skowronski, N. Z. Janjua, G. De Serres, S. Sabaiduc, A. Eshaghi, J. A. Dickinson, K. Fonseca, A. L. Winter, J. B. Gubbay, M. Krajden, M. Petric, H. Charest, N. Bastien, T. L. Kwindt, S. M. Mahmud, P. Van Caeseele, and Y. Li. Low 2012-13 influenza vaccine effectiveness associated with mutation in the egg-adapted h3n2 vaccine strain not antigenic drift in circulating viruses. *PLoS One*, 9(3):e92153, 2014.

[63] D. M. Skowronski, N. Z. Janjua, S. Sabaiduc, G. De Serres, A. L. Winter, J. B. Gubbay, J. A. Dickinson, K. Fonseca, H. Charest, N. Bastien, Y. Li, T. L. Kwindt, S. M. Mahmud, P. Van Caeseele, M. Krajden, and M. Petric. Influenza a/subtype and b/lineage effectiveness estimates for the 2011-2012 trivalent vaccine: cross-season and cross-lineage protection with unchanged vaccine. *J Infect Dis*, 210(1):126–37, 2014.

[64] D. M. Skowronski, C. Chambers, S. Sabaiduc, G. De Serres, A. L. Winter, J. A. Dickinson, J. Gubbay, K. Fonseca, H. Charest, M. Krajden, M. Petric, S. M. Mahmud, P. Van Caeseele, N. Bastien, A. Eshaghi, and Y. Li. Integrated sentinel surveillance linking genetic, antigenic, and epidemiologic monitoring of influenza vaccine-virus relatedness and effectiveness during the 2013-2014 influenza season. *J Infect Dis*, 212(5):726–39, 2015.

[65] D. M. Skowronski, C. Chambers, S. Sabaiduc, G. De Serres, A. L. Winter, J. A. Dickinson, M. Krajden, J. B. Gubbay, S. J. Drews, C. Martineau, A. Eshaghi, T. L. Kwindt, N. Bastien, and Y. Li. A perfect storm: Impact of genomic variation and serial vaccination on low influenza vaccine effectiveness during the 2014-2015 season. *Clin Infect Dis*, 63(1):21–32, 2016.

[66] D. M. Skowronski, C. Chambers, G. De Serres, S. Sabaiduc, A. L. Winter, J. A. Dickinson, J. B. Gubbay, K. Fonseca, S. J. Drews, H. Charest, C. Martineau, M. Krajden, M. Petric, N. Bastien, Y. Li, and D. J. Smith. Serial vaccination and the antigenic distance hypothesis: Effects on influenza vaccine effectiveness during a(h3n2) epidemics in canada, 2010-2011 to 2014-2015. *J Infect Dis*, 215(7):1059–1099, 2017.

[67] D. M. Skowronski, C. Chambers, S. Sabaiduc, G. De Serres, A. L. Winter, J. A. Dickinson, J. B. Gubbay, S. J. Drews, C. Martineau, H. Charest, M. Krajden, N. Bastien, and Y. Li. Beyond antigenic match: Possible agent-host and immuno-epidemiological influences on influenza vaccine effectiveness during the 2015-2016 season in canada. *J Infect Dis*, 216(12):1487–1500, 2017.

[68] D. M. Skowronski, C. Chambers, G. De Serres, S. Sabaiduc, A. L. Winter, J. A. Dickinson, J. B. Gubbay, S. J. Drews, K. Fonseca, H. Charest, C. Martineau, R. Hickman, T. Chan, A. Jassem, M. Petric, C. Rose, N. Bastien, Y. Li, and M. Krajden. Vaccine effectiveness against lineage-matched and -mismatched influenza b viruses across 8 seasons in canada, 2010-2011 to 2017-2018. *Clin Infect Dis*, 68(10):1754–1757, 2019.

[69] D. M. Skowronski, S. Sabaiduc, S. Leir, C. Rose, M. Zou, M. Murti, J. A. Dickinson, R. Olsha, J. B. Gubbay, M. A. Croxen, H. Charest, N. Bastien, Y. Li, A. Jassem, M. Krajden, and G. De Serres. Paradoxical clade- and age-specific vaccine effectiveness during the 2018/19 influenza a(h3n2) epidemic in canada: potential imprint-regulated effect of vaccine (i-rev). *Euro Surveill*, 24(46):1900585, 2019.

[70] D. M. Skowronski, S. Leir, S. Sabaiduc, C. Chambers, M. Zou, C. Rose, R. Olsha, J. A. Dickinson, A. L. Winter, A. Jassem, J. B. Gubbay, S. J. Drews, H. Charest, T. Chan, R. Hickman, N. Bastien, Y. Li, M. Krajden, and G. De Serres. Influenza vaccine effectiveness by a(h3n2) phylogenetic sub-cluster and prior vaccination history: 2016-17 and 2017-18 epidemics in canada. *J Infect Dis*, 2022.

[71] M. Smithgall, C. Y. Vargas, C. Reed, L. Finelli, P. LaRussa, E. L. Larson, L. Saiman, and M. S. Stockwell. Influenza vaccine effectiveness in a low-income, urban community cohort. *Clin Infect Dis*, 62(3):358–360, 2016.

[72] J. Y. Song, J. Y. Noh, J. S. Lee, S. H. Wie, Y. K. Kim, J. Lee, H. W. Jeong, S. W. Kim, S. H. Lee, K. H. Park, W. S. Choi, H. J. Cheong, and W. J. Kim. Effectiveness of repeated influenza vaccination among the elderly population with high annual vaccine uptake rates during the three consecutive a/h3n2 epidemics. *Vaccine*, 38(2):318–322, 2020.

[73] S. G. Sullivan and H. Kelly. Stratified estimates of influenza vaccine effectiveness by prior vaccination: caution required. *Clin Infect Dis*, 57(3):474–6, 2013.

[74] S. G. Sullivan, M. B. Chilver, K. S. Carville, Y. M. Deng, K. A. Grant, G. Higgins, N. Komadina, V. K. Leung, C. A. Minney-Smith, D. Teng, T. Tran, N. Stocks, and J. E. Fielding. Low interim influenza vaccine effectiveness, australia, 1 may to 24 september 2017. *Euro Surveill*, 22(43):17–00707, 2017.

[75] R. K. Syrjanen, J. Jokinen, T. Ziegler, J. Sundman, M. Lahdenkari, I. Julkunen, and T. M. Kilpi. Effectiveness of pandemic and seasonal influenza vaccines in preventing laboratory-confirmed influenza in adults: a clinical cohort study during epidemic seasons 2009-2010 and 2010-2011 in finland. *PLoS One*, 9(9):e108538, 2014.

[76] M. G. Thompson, D. K. Li, P. Shifflett, L. Z. Sokolow, J. R. Ferber, S. Kurosky, S. Bozeman, S. B. Reynolds, R. Odouli, M. L. Henninger, T. L. Kauffman, L. A. Avalos, S. Ball, J. L. Williams, S. A. Irving, D. K. Shay, A. L. Naleway, Pregnancy, and Workgroup Influenza Project. Effectiveness of seasonal trivalent influenza vaccine for preventing influenza virus illness among pregnant women: a population-based case-control study during the 2010-2011 and 2011-2012 influenza seasons. *Clin Infect Dis*, 58(4):449–57, 2014.

[77] M. G. Thompson, A. Naleway, A. M. Fry, S. Ball, S. M. Spencer, S. Reynolds, S. Bozeman, M. Levine, J. M. Katz, and M. Gaglani. Effects of repeated annual inactivated influenza vaccination among healthcare personnel on serum hemagglutinin inhibition antibody response to a/perth/16/2009 (h3n2)-like virus during 2010-11. *Vaccine*, 34(7):981–8, 2016.

[78] M. Valenciano, E. Kissling, A. Reuss, C. Rizzo, A. Gherasim, J. K. Horvath, L. Domegan, D. Pitigoi, A. Machado, I. A. Paradowska-Stankiewicz, A. Bella, A. Larrauri, A. Ferenczi, O. Donell Joan, M. Lazar, P. Pechirra, M. R. Korczynska, F. Pozo, A. Moren, and I. Move multicentre case-control team. Vaccine effectiveness in preventing laboratory-confirmed influenza in primary care patients in a season of co-circulation of influenza a(h1n1)pdm09, b and drifted a(h3n2), i-move multicentre case-control study, europe 2014/15. *Euro Surveill*, 21(7):pii=30139, 2016.

[79] M. Valenciano, E. Kissling, A. Larrauri, B. Nunes, D. Pitigoi, J. O'Donnell, A. Reuss, J. K. Horvath, I. Paradowska-Stankiewicz, C. Rizzo, A. Falchi, I. Daviaud, M. Brytting, A. Meijer, B. Kaic, A. Gherasim, A. Machado, A. Ivanciuc, L. Domegan, B. Schweiger, A. Ferenczi, M. Korczynska, A. Bella, A. M. Vilcu, A. Mosnier, K. Zakikhany, M. de Lange, S. Kurecic Filipovicovic, K. Johansen, A. Moren, and I. Move primary care multicentre case-control team. Exploring the effect of previous inactivated influenza vaccination on seasonal influenza vaccine effectiveness against medically attended influenza: Results of the european i-move multicentre test-negative case-control study, 2011/2012-2016/2017. *Influenza Other Respir Viruses*, 12(5):567–581, 2018.

[80] L. Zhang, P. Yang, M. G. Thompson, Y. Pan, C. Ma, S. Wu, Y. Sun, M. Zhang, W. Duan, and Q. Wang. Influenza vaccine effectiveness in preventing influenza illness among children during school-based outbreaks in the 2014-2015 season in beijing, china. *Pediatr Infect Dis J*, 36(3):e69–e75, 2017.

[81] L. Zhang, Y. Pan, V. Hackert, W. van der Hoek, A. Meijer, T. Krafft, P. Yang, and Q. Wang. The 2015-2016 influenza epidemic in beijing, china: Unlike elsewhere, circulation of influenza a(h3n2) with moderate vaccine effectiveness. *Vaccine*, 36(33):4993–5001, 2018.

[82] L. Zhang, W. van der Hoek, T. Krafft, E. Pilot, L. V. Asten, G. Lin, S. Wu, W. Duan, P. Yang, and Q. Wang. Influenza vaccine effectiveness estimates against influenza a(h3n2) and a(h1n1) pdm09 among children during school-based outbreaks in the 2016-2017 season in beijing, china. *Hum Vaccin Immunother*, 16(4):816–822, 2020.

[83] R. K. Zimmerman, M. P. Nowalk, J. Chung, M. L. Jackson, L. A. Jackson, J. G. Petrie, A. S. Monto, H. Q. McLean, E. A. Belongia, M. Gaglani, K. Murthy, A. M. Fry, B. Flannery, U. S. Flu VE Investigators, and U. S. Flu VE Investigators. 2014-2015 influenza vaccine effectiveness in the united states by vaccine type. *Clin Infect Dis*, 63(12):1564–1573, 2016.
